# Improved Synchronous Characterization Theory for Surface and Interface Mechanical Properties of Thin-Film/Substrate Systems: A Theoretical Study on Shaft-Loaded Blister Test Technique

**DOI:** 10.3390/ma17205054

**Published:** 2024-10-16

**Authors:** Xiao-Ting He, Xiang Li, He-Hao Feng, Jun-Yi Sun

**Affiliations:** 1 School of Civil Engineering, Chongqing University, Chongqing 400045, China; 202216131356@stu.cqu.edu.cn (X.L.); 202216131289t@stu.cqu.edu.cn (H.-H.F.); sunjunyi@cqu.edu.cn (J.-Y.S.); 2 Key Laboratory of New Technology for Construction of Cities in Mountain Area (Chongqing University), Ministry of Education, Chongqing 400045, China

**Keywords:** thin-film/substrate system, shaft-loaded blister test, thin-film/substrate delamination, large deflection, analytical solution, synchronous characterization

## Abstract

In this paper, the previously proposed shaft-loaded blister test technique for the synchronous characterization of the surface and interface mechanical properties of a thin-film/substrate system is further studied theoretically. The large deflection problem of the steady shaft-loaded blistering thin film is reformulated by surrendering the small-rotation-angle assumption of the membrane, which was previously adopted in the out-of-plane and in-plane equilibrium and radial geometric equations. A new and more accurate analytical solution to this large deflection problem is presented and is used to improve the previously presented synchronous characterization theory. The new analytical solution is numerically compared with the previous analytical solution to confirm the superiority of the new analytical solution over the previous analytical solution. An experiment is conducted to verify the beneficial effect of the improved synchronous characterization theory on improving the characterization accuracy.

## 1. Introduction

Thin films bonded to rigid substrates, usually called thin-film/substrates, are commonly encountered in a variety of applications [1,2,3,4,5]. The reliability of a thin-film/substrate system depends strongly on the adhesive property of its surface thin film, so mechanical characterization for the interfacial adhesion strength of the thin-film/substrate system (to quantify the adhesive strength) is often found to be necessary [6,7,8,9,10]. The interface characterization needs to delaminate the surface thin film attached to the substrate from the substrate, where the work done by the external force that causes delamination to the delamination system minus the elastic energy stored in the steady thin film after delamination is the interfacial energy of adhesion released on the delamination area (expended in the creation of the delamination area). Since the calculation of the elastic energy stored in the steady thin film after delamination requires prior knowledge of the Poisson’s ratio *v* and Young’s modulus of elasticity *E* of the surface thin film, and the mechanical properties of the surface thin film may have large differences in them due to variations in processing conditions such as the temperature, humidity, or the order of fabrication procedures [11,12,13,14,15], so it is desirable that the Poisson’s ratio *v* and Young’s modulus of elasticity *E* of the surface thin film is also able to be measured at the same time as the measurement of the interfacial energy of adhesion. In other words, it is expected that the surface and interface mechanical properties (Poisson’s ratio *v*, Young’s modulus of elasticity *E*, and interfacial adhesion energy) of a thin-film/substrate can be simultaneously determined by the experimental data from the same thin-film/substrate delamination experiment, which is the so-called synchronous characterization technique for the surface and interface mechanical properties of thin-film/substrate systems [16,17,18,19,20].

There are two ways to achieve thin-film/substrate delamination: one is the conventional peeling test [21,22,23,24,25], and the other is the well-known blister test [26,27,28,29,30]. The disadvantage of the peeling test is that the unavoidable plastic deformation consumes most of the energy dissipation and obscures the small interfacial energy of adhesion, which is the required quantity [31,32,33,34,35]. The blister test is considered to be the more advantageous method and is usually classified into two major variants by the interfacial crack driving force: one is the pressurized blister test [36,37,38,39,40], and the other is the shaft-loaded blister test [41,42,43,44,45]. The main attractions of the pressurized blister test are the axisymmetric blistering geometry and the small rotation angle of the blistering thin film at the interfacial crack front [46,47,48,49,50], and its main disadvantages are the difficulty in controlling the pressure to avoid catastrophic debonding and the difficulty to monitor the simultaneous change in pressure and blister dimension [51,52,53,54,55]. The shaft-loaded blister test was proposed originally by Malyshev and Salganik [56] and can overcome these difficulties [57,58,59,60,61]. The main disadvantage of the conventional shaft-loaded blister test is that the theoretical simplification of the shaft-loading as a model of a central point load requires that the loading shaft used must be very slender [62,63,64,65,66], which can easily cause plastic yielding and piercing of the shaft-loaded blistering thin film. So, in the previous study [67], we suggested using a cylinder (or a cylindrical punch) with a finite radius and frictionless flat end as the loading shaft, where the surface thin film of a thin-film/substrate system specimen is delaminated from the substrate by the cylindrical loading-shaft that is driven by a screw rod.

However, in our previous study [67], we failed to present the exact analytical solution for the large deflection problem of the shaft-loaded blistering thin film in the shaft-loaded blister test. The exact analytical solution is essential to ensure the accuracy of the synchronous characterization theory for the surface and interface mechanical properties of thin-film/substrate systems. But the analytical solution in [67] follows the assumption of the small-rotation-angle of the membrane, which affects not only its accuracy but also its application in large deflection situations (e.g., soft, very thin polymer coatings with low modulus of elasticity). In this study, the small-rotation-angle assumption adopted in [67] is completely surrendered, and a new and more accurate analytical solution is obtained, thus improving the previously presented synchronous characterization theory for the surface and interface mechanical properties of thin-film/substrate systems.

The paper is organized as follows. The materials and methods are shown in the following section, where the exact analytical solution to the large deflection problem of the shaft-loaded blistering thin-film in the shaft-loaded blister test is detailed in Section 2.1, how to simultaneously determine the Poisson’s ratios and Young’s modulus of elasticity of the surface thin-film of a thin-film/substrate system specimen is shown in Section 2.2, how to determine the elastic energy stored in the steady shaft-loaded blistering thin-film is shown in Section 2.3, and the operation process of experiment and synchronous characterization are summarized in Section 2.4. The results and discussion are shown in Section 3, where the newly and previously presented analytical solutions are compared in Section 3.1, an experiment for demonstrating the simultaneous determination of Poisson’s ratios and Young’s modulus of elasticity is conducted in Section 3.2, and a numerical example to illustrate how to calculate the elastic energy stored in the steady shaft-loaded blistering thin-film is provided in Section 3.3. The concluding remarks are provided in Section 4. It can be concluded from this study that the synchronous characterization theory after improvement has higher characterization precision than the one before improvement.

## 2. Materials and Methods

**Figure 1:**
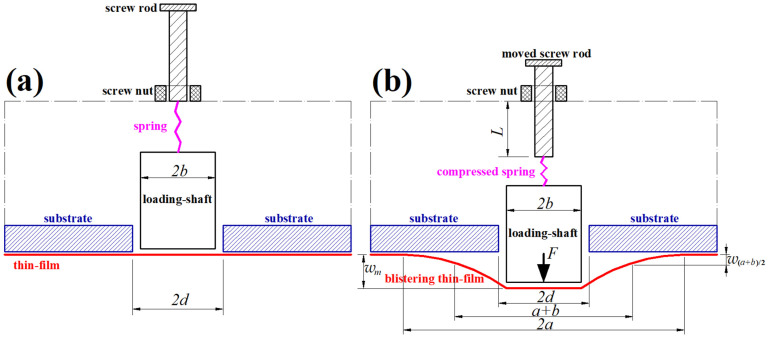
Schematic illustration of a shaft-loaded blister test for the synchronous characterization of the surface and interface mechanical properties of a thin-film/substrate system specimen: (**a**) A small circular hole with radius *d* runs through the substrate of the thin-film/substrate system specimen and leaves the surface thin film completely exposed and suspended; (**b**) a cylindrical loading shaft with radius *b* delaminates the surface thin film that still adheres to the substrate from the substrate.

Figure 1 shows the previously proposed synchronous characterization of the surface and interface mechanical properties of a thin-film/substrate system based on the shaft-loaded blister test [67]. On the substrate of a thin-film/substrate system specimen, a small circular hole with radius *d* is drilled (or is chemically etched) until it reaches the thin-film/substrate interface and leaves the surface thin film completely exposed, as shown in Figure 1a, where the exposed thin film is initially flat and suspended, the loading-shaft with radius *b* (*b* is less than but closed to *d*) is a cylinder (or a cylindrical punch) with a circular frictionless flat end that is parallel to the initially flat, suspended thin film and is just in contact with the suspended thin-film at this time, the other end of the cylinder is connected to a spring (which is in its initial undeformed natural state at this time), the other end of the spring is connected to a screw rod (the screw nut serving as the support point of the loading system should be fixed to rigid support), and the cylinder is assumed to be so light that its weight can be negligible.

The screw rod is rotated and moved by a displacement *L*, as shown in Figure 1b. This makes the spring compressed and produces a compressive force *F*, which first acts on the cylindrical loading shaft and eventually acts on the suspended thin film through the cylindrical loading shaft and delaminates the surface thin film that still adheres to the substrate from the substrate. When the delamination stops, that is, when the shaft-loaded blistering thin-film reaches static equilibrium (steady, no change in size), the maximum thin film deflection of the steady shaft-loaded blistering thin film will reach *w_m_*_,_ and its delamination radius will reach *a*, as shown in Figure 1b. Therefore, the delamination area *S_d_* reaches π(*a*^2^ − *d*^2^), the compressed length of the spring reaches *L* − *w_m_*, and the compressive force *F* of the spring reaches *k_x_*(*L* − *w_m_*), where *k_x_* is the linear elastic stiffness coefficient of the spring. Then, the work *U_F_* done by the spring force *F* (increasing from zero to *k_x_*(*L* − *w_m_*)) to the delamination system may be written as

(1) $$U_{F}=\int_{0}^{L-w_{m}}F(l)dl=\frac{1}{2}k_{x}{\left(L-w_{m}\right)}^{2},$$

where the maximum thin-film deflection *w*_m_ of the steady shaft-loaded blistering thin film and the distance *L* by which the screw rod is moved are two quantities to be measured. The other two quantities to be measured are the delamination radius *a* of the steady shaft-loaded blistering thin film and its thin-film deflection *w*_(*a*+*b*)/2_ (i.e., the thin-film deflection at the middle of the annular region, see Figure 1b).

On the one hand, with the above four quantities to be measured, the Poisson’s ratio *v* and Young’s modulus of elasticity *E* of the surface thin film of the thin-film/substrate system specimen can be simultaneously determined, as long as the analytical solution for the large deflection elastic behavior of the steady shaft-loaded blistering thin film (i.e., the shaft-loaded blistering thin film that reaches static equilibrium and produces the steady delamination radius *a* and maximum deflection *w_m_*, see Figure 1b) is available. The specific details for the simultaneous determination of *v* and *E* are shown in Section 2.2.

On the other hand, obviously, the interfacial energy of adhesion, *U_i_*, released as the interface crack extends a distance (*a* − *d*) along the thin-film/substrate interface should be equal to the work *U_F_* (done by the spring force *F* to the delamination system) minus the elastic energy *U_e_* (stored in the steady shaft-loaded blistering thin film). Therefore, the energy release rate *G* may be written as

(2) $$G=\frac{U_{i}}{S_{d}}=\frac{U_{F}-U_{e}}{\pi (a^{2}-d^{2})}=\frac{k_{x}{\left(L-w_{m}\right)}^{2}-2U_{e}}{2\pi (a^{2}-d^{2})},$$

where the elastic energy *U_e_* can be determined as long as the analytical solution for the large deflection elastic behavior of the steady shaft-loaded blistering thin film is available.

Therefore, it is particularly important here to be able to analytically solve the large deflection elastic behavior of the steady shaft-loaded blistering thin film accurately.

### 2.1. Analytical Solution to the Elastic Behavior of the Steady Shaft-Loaded Blistering Thin Film

**Figure 2:**
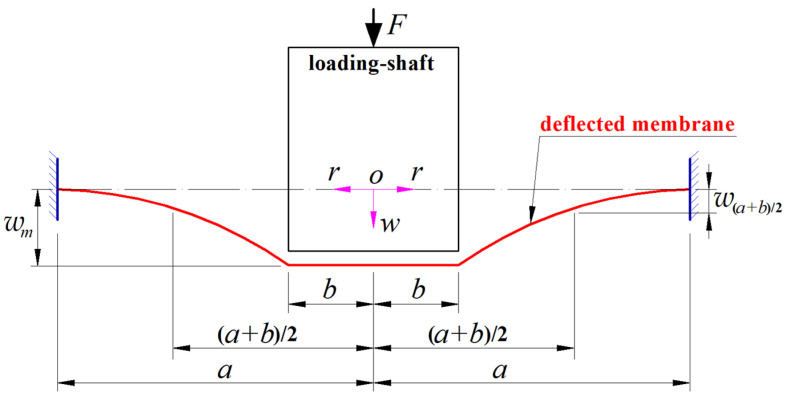
A profile along a diameter of the initially flat, peripherally fixed, deflected circular membrane under the action of the loading shaft.

Since the loading shaft in Figure 1 is a cylinder (or a cylindrical punch) whose flat end of radius *b* is in frictionless parallel contact with the initially flat, suspended thin film, the shaft-loading spring force *F* is, in fact, uniformly distributed along a circle with radius *b*. Therefore, the elastic behavior of the steady shaft-loaded blistering thin film in Figure 1b can be modeled, in mechanics, as a large deflection problem of axisymmetric deformation of an initially flat, peripherally fixed circular membrane subjected to the action of a uniformly distributed axisymmetric line loads, as shown in Figure 2, where the circular membrane inside the circle with radius *b* is always in a state of being radially and horizontally stretched. Such a large deflection problem of axisymmetric deformation can be analytically solved by establishing the mathematical and physical equations for force equilibriums, strain–displacement geometric relationships, and stress–strain physical relationships, as detailed in any general theory of plates and shells. This large deflection problem of axisymmetric deformation has been dealt with analytically in [67], where the established out-of-plane equilibrium equation, in-plane equilibrium equation, and geometric equations adopt the condition that the rotation-angle of the membrane, *θ*, is assumed to be very small, i.e., the usually so-called small-rotation-angle assumption of the membrane. Therefore, the closed-form solution of the large deflection problem, which was presented in [67], is suitable only for applications with small membrane rotation angles (usually *θ* is less than five degrees). However, the rotation-angle *θ* of the blistering thin film in the shaft-loaded blister test proposed in [67] is often much larger than five degrees. Therefore, the closed-form solution presented in [67] limits the use of the proposed shaft-loaded blister test technique. In this study, the used out-of-plane equilibrium equation, in-plane equilibrium equation, and geometric equations are all established under the condition of surrendering the small-rotation-angle assumption of the membrane, so the application of the closed-form solution presented here is not limited by the size of the membrane rotation angle.

As shown in Figure 2, a cylindrical coordinate system (*r*, *φ*, *w*) is introduced, where *r* is the radial coordinate variable, *φ* is the angle coordinate variable (not represented in Figure 1 due to the rotational symmetry), *w* is the cylindrical coordinate variable (*w* also represents the membrane deflection), *a* represents the radius of the initially flat, peripherally fixed circular membrane with Young’s modulus of elasticity *E*, Poisson’s ratio *v* and thickness *h*, *b* represents the radius of the cylindrical loading shaft (a cylindrical punch), *F* represents the external load applied to the loading shaft (the spring force *F* = *k_x_*(*L* − *w_m_*) in Figure 1b), *w*_(*a*+*b*)/2_ represents the membrane deflection at *r* = (*a* + *b*)/2, *w_m_* represents the maximum membrane deflection at *r* = *b*, the polar plane (*r*, *φ*) passes through the geometric middle plane (the dash–dotted line in Figure 2) of the exposed initially flat, suspended thin film (see Figure 1a), and the coordinate origin *o* of the introduced cylindrical coordinate system (*r*, *φ*, *w*) coincides with the centroid of the geometric middle plane. This large deflection problem of axisymmetric deformation of the initially flat, peripherally fixed circular membrane subjected to the action of a uniformly distributed axisymmetric line loads is divided into the large deflection problem within *b* ≤ *r* ≤ *a* and the radial horizontal stretching problem within *0* ≤ *r* ≤ *b*, and it is analytically solved as follows.

**Figure 3:**
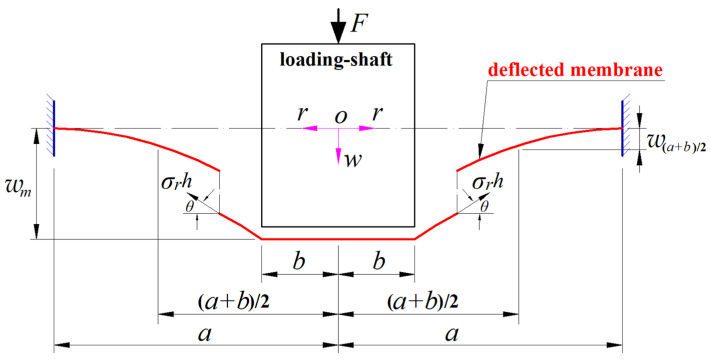
A free body with radius *r* (*b* ≤ *r* ≤ *a*) under the joint action of the external force *F* and the membrane force *σ_r_h*, which is taken from the central region of the deflected circular membrane in Figure 2.

The out-of-plane equilibrium equation can be established by taking a free body with radius *b* ≤ *r* ≤ *a* from the central region of the deflected circular membrane in Figure 2, as shown in Figure 3, where *σ_r_* is the abbreviation for *σ_r_*(*r*) that denotes the radial stress of the circular membrane at *r*, and *θ* is the abbreviation for *θ*(*r*) that denotes the rotation angle of the circular membrane at *r*. In the vertical direction perpendicular to the polar plane (*r*, *φ*), there is the force *F* and the vertical component *σ_r_h*sin*θ* of the membrane force *σ_r_h*. Therefore, the condition of the resultant force being equal to zero in the vertical direction provides the out-of-plane equilibrium equation

(3) $$F-2\pi r\sigma_{r}h\sin\theta =0.$$

Since the deflection of the membrane at *r* is *w*(*r*) (abbreviated to *w*), then

(4) $$\sin\theta =1/\sqrt{1+1/\tan^{2}\theta }=1/\sqrt{1+1/{\left(-dw/dr\right)}^{2}}.$$

After substituting Equation (4) into Equation (3), the out-of-plane equilibrium equation without the small-rotation-angle assumption may be written as

(5) $$F-2\pi r\sigma_{r}h/\sqrt{1+1/{\left(-dw/dr\right)}^{2}}=0.$$

The in-plane equilibrium equation without the small-rotation-angle assumption can be established by taking a micro area element (formed by the radial coordinate increment Δ*r* and circumferential coordinate increment Δ*φ*, see [68] for details) on the deflected circular membrane, and is provided by [68]

(6) $$\frac{d}{dr}(\frac{r\sigma_{r}}{\sqrt{1+{\left(-dw/dr\right)}^{2}}})-\sigma_{t}\sqrt{1+{\left(-dw/dr\right)}^{2}}=0,$$

where *σ_t_* is the abbreviation for *σ_t_*(*r*) that denotes the circumferential stress of the circular membrane at *r*.

The radial and circumferential geometric equations without the small-rotation-angle assumption can be established by taking radial and circumferential micro line elements on the deflected circular membrane and is provided by [69]

(7) $$e_{r}=\sqrt{{\left(1+\frac{du}{dr}\right)}^{2}+{\left(\frac{dw}{dr}\right)}^{2}}-1$$

and

(8) $$e_{t}=\frac{u}{r},$$

where *u*, *e_r,_* and *e_t_* are the abbreviations for *u*(*r*), *e_r_*(*r*), and *e_t_*(*r*) that respectively denote the radial displacement, radial strain, and circumferential strain of the circular membrane at *r*.

The membrane used is assumed to be Hooke-type linear elastic material, so the relationships between stresses and strains, i.e., the so-called physical equations, are provided by the generalized Hooke’s law

(9) $$\sigma_{r}=\frac{E}{1-v^{2}}(e_{r}+ve_{t})\;\mathrm{and}\;\sigma_{t}=\frac{E}{1-v^{2}}(e_{t}+ve_{r})$$

or

(10) $$e_{r}=\frac{1}{E}(\sigma_{r}+v\sigma_{t})\;\mathrm{and}\;e_{t}=\frac{1}{E}(\sigma_{t}+v\sigma_{r}).$$

where *v* and *E,* respectively, denote the Poisson’s ratio and Young’s modulus of elasticity of the used membrane.

Since Equations (5)–(9), or Equations (5)–(8) and (10), contain only six physical quantities and one independent variable, i.e., the radial stress *σ_r_*, circumferential stress *σ_t_*, radial strain *e_r_*, circumferential strain *e_t_*, radial displacement *u*, transverse displacement (deflection) *w*, and the radial coordinate (as independent variable) *r*, each of the six physical quantities can be expressed as the function with respect to the radial coordinate *r* by simultaneously solving Equations (5)–(9), or Equations (5)–(8) and (10). To this end, let us first eliminate the radial strain *e_r_*, circumferential strain *e_t,_* and radial displacement *u* from Equations (7)–(10). By substituting Equations (7)–(8) into Equation (9) to eliminate *e_r_* and *e_t_*, it is found that

(11) $$\sigma_{r}=\frac{E}{1-v^{2}}[\sqrt{{\left(1+\frac{du}{dr}\right)}^{2}+{\left(\frac{dw}{dr}\right)}^{2}}-1+v\frac{u}{r}]$$

and

(12) $$\sigma_{t}=\frac{E}{1-v^{2}}[\frac{u}{r}+v\sqrt{{\left(1+\frac{du}{dr}\right)}^{2}+{\left(\frac{dw}{dr}\right)}^{2}}-v].$$

From Equation (8) and the second expression of Equation (10), it can be found that

(13) $$e_{t}=\frac{u}{r}=\frac{1}{E}(\sigma_{t}-v\sigma_{r}).$$

By substituting *u* in Equation (13) into Equation (11), it is found that

(14) $${\left(\sigma_{r}-\nu \sigma_{t}+E\right)}^{2}-{\left[\sigma_{t}-v\sigma_{r}+E+r\frac{d}{dr}(\sigma_{t})-r\frac{d}{dr}(v\sigma_{r})\right]}^{2}-E^{2}{\left(\frac{dw}{dr}\right)}^{2}=0.$$

Therefore, the general solutions of the radial stress *σ_r_*, circumferential stress *σ_t_*, and deflection *w* can be obtained by simultaneously solving Equations (5), (6), and (14). In addition, after the expressions of *σ_r_*, *σ_t,_* and *w* are known, the expressions of the radial strain *e_r_*, circumferential strain *e_t,_* and radial displacement *u* can easily be derived from Equations (10) and (13).

However, usually the particular solutions of the physical quantities of *σ_r_*, *σ_t_*, *e_r_*, *e_t_*, *u*, and *w* are necessary, which need boundary conditions and continuity conditions. The boundary conditions at *r* = *a* are

(15) $$e_{t}=\frac{u}{r}=\frac{1}{E}(\sigma_{t}-v\sigma_{r})=0\;\mathrm{at}\;r=a$$

and

(16) w=0 at r=a.

The continuity conditions at *r* = *b* are

(17) $${\left(\sigma_{r}\right)}_{A}={\left(\sigma_{r}\right)}_{B}\;\mathrm{at}\;r=b$$

and

(18) $${\left(\sigma_{t}\right)}_{A}={\left(\sigma_{t}\right)}_{B}\;\mathrm{at}\;r=b,$$

where the two symbols ( )*_A_* and ( )*_B_* represent the two sides of the interconnecting circle with radius *b*, and the subscript *A* refers to the side inside the interconnecting circle with radius *b*, while the subscript *B* refers to the side outside the interconnecting circle with radius *b*. So, (*σ_r_*)*_A_* and (*σ_t_*)*_A_* refer to the values of the radial stress *σ_r_* and circumferential stress *σ_t_* at *r* = *b* for the large deflection problem within *b* ≤ *r* ≤ *a*, while (*σ_r_*)*_B_* and (*σ_t_*)*_B_* refer to the values of the radial stress *σ_r_* and circumferential stress *σ_t_* at *r* = *b* for the radial horizontal stretching problem within *0* ≤ *r* ≤ *b*.

Now, we further deal with the radial horizontal stretching problem within *0* ≤ *r* ≤ *b*. It is obvious that d*w*/d*r* = 0 within *0* ≤ *r* ≤ *b*. Therefore, substituting d*w*/d*r* = 0 into Equations (6), (7), (11), and (12) yields

(19) $$\frac{d}{dr}(r\sigma_{r})-\sigma_{t}=0,$$

(20) $$e_{r}=\frac{du}{dr},$$

(21) $$\sigma_{r}=\frac{E}{1-v^{2}}(\frac{du}{dr}+v\frac{u}{r})$$

and

(22) $$\sigma_{t}=\frac{E}{1-v^{2}}(v\frac{du}{dr}+\frac{u}{r}).$$

Substituting Equations (21) and (22) into Equation (19) yields

(23) $$r^{2}\frac{d^{2}u}{dr^{2}}+r\frac{du}{dr}-u=0.$$

Equation (23) can be solved under the conditions that *u* = 0 at *r* = 0 and *u* = *u*_B_ at *r* = *b*, where *u*_B_ is a value to be determined. Since Equation (23) satisfies the form of Euler’s equation, its general solution can be written as

(24) $$u=C_{1}r+\frac{C_{2}}{r},$$

where *C*_1_ and *C*_2_ are two arbitrary constants. Obviously, in order to satisfy the condition that *u* = 0 at *r* = 0, *C*_2_ must be equal to zero. And then, from the condition that *u* = *u*_B_ at *r* = *b*, it can be found that *C*_1_ = *u*_B_/*b*. Therefore, the particular solution of Equation (23) may be written as

(25) $$u=\frac{u_{B}}{b}r.$$

Further, substituting Equation (25) into Equations (20)–(22) and (8) yields

(26) $$\sigma_{r}=\frac{Eu_{B}}{(1-v)b},$$

(27) $$\sigma_{t}=\frac{Eu_{B}}{(1-v)b},$$

(28) $$e_{r}=\frac{u_{B}}{b}$$

and

(29) $$e_{t}=\frac{u_{B}}{b}.$$

Therefore, as long as *u*_B_ can be determined, the radial horizontal stretching problem within 0≤ *r* ≤ *b* can be solved analytically.

For the large deflection problem within *b* ≤ *r* ≤ *a*, from Equations (26) and (27), the continuity conditions at *r* = *b*, i.e., Equations (17) and (18), can be rewritten as

(30) $${\left(\sigma_{r}\right)}_{A}={\left(\sigma_{r}\right)}_{B}=\frac{Eu_{B}}{(1-v)b}\;\mathrm{at}\;r=b$$

and

(31) $${\left(\sigma_{t}\right)}_{A}={\left(\sigma_{t}\right)}_{B}=\frac{Eu_{B}}{(1-v)b}\;\mathrm{at}\;r=b.$$

We introduce the following dimensionless variables

(32) $$P=\frac{F}{a\pi hE},S_{r}=\frac{\sigma_{r}}{E},S_{t}=\frac{\sigma_{t}}{E},W=\frac{w}{a},x=\frac{r}{a},\alpha =\frac{b}{a},$$

and transform Equations (5), (6), (14)–(16), (30), and (31) into

(33) $$(4x^{2}S_{r}^{2}-P^{2}){\left(-\frac{dW}{dx}\right)}^{2}-P^{2}=0,$$

(34) $$\frac{d(xS_{r})}{dx}[1+{\left(-\frac{dW}{dx}\right)}^{2}]-xS_{r}\frac{dW}{dx}\frac{d^{2}W}{dx^{2}}-S_{t}{\left[1+{\left(-\frac{dW}{dx}\right)}^{2}\right]}^{2}=0,$$

(35) $${\left(S_{r}-\nu S_{t}+1\right)}^{2}-{\left[S_{t}-vS_{r}+1+x\frac{d}{dx}(S_{t})-x\frac{d}{dx}(vS_{r})\right]}^{2}-{\left(\frac{dW}{dx}\right)}^{2}=0,$$

(36) W=0 at x=1,

(37) $$S_{t}-vS_{r}=0\;\mathrm{at}\;x=1,$$

(38) $$S_{r}=\frac{u_{B}}{b(1-v)}\;\mathrm{at}\;x=\alpha$$

and

(39) $$S_{t}=\frac{u_{B}}{b(1-v)}\;\mathrm{at}\;x=\alpha .$$

It can easily be found, from Equations (38) and (39), that

(40) $$S_{r}=S_{t}\;\mathrm{at}\;x=\alpha .$$

In order to use the power series method to solve Equations (33)–(35), *S_r_*(*x*), *S_t_*(*x*), and *W*(*x*) must be expanded into the power series of the *x* − (1 + *α*)/2 (in which *α* ≤ *x* ≤ 1); that is,

(41) $$S_{r}(x)=\sum_{i=0}^{\infty }b_{i}X^{i},$$

(42) $$S_{t}(x)=\sum_{i=0}^{\infty }c_{i}X^{i}$$

and

(43) $$W(x)=\sum_{i=0}^{\infty }d_{i}X^{i},$$

where *X* = *x* − *β*, *β* = (1 + *α*)/2, and *β* − 1 ≤ *X* ≤ 1 − *β*. Since *x* = *X* + *β*, then Equations (33)–(37) and (40) can be transformed into

(44) $$[4{\left(X+\beta \right)}^{2}S_{r}^{2}-P^{2}]{\left(-\frac{dW}{dX}\right)}^{2}-P^{2}=0,$$

(45) $$[1+{\left(-\frac{dW}{dX}\right)}^{2}]\frac{d}{dX}[(X+\beta )S_{r}]-(X+\beta )S_{r}\frac{dW}{dX}\frac{d^{2}W}{dX^{2}}-S_{t}{\left[1+{\left(-\frac{dW}{dX}\right)}^{2}\right]}^{2}=0,$$

(46) $${\left(S_{r}-vS_{t}+1\right)}^{2}-{\left[S_{t}-vS_{r}+1+(X+\beta )\frac{d}{dX}(S_{t})-(X+\beta )\frac{d}{dX}(vS_{r})\right]}^{2}-{\left(\frac{dW}{dX}\right)}^{2}=0,$$

(47) W=0 at X=1−β,

(48) $$S_{t}-vS_{r}=0\;\mathrm{at}\;X=1-\beta ,$$

and

(49) $$S_{r}=S_{t}\;\mathrm{at}\;X=\beta -1.$$

After substituting Equations (41)–(43) into Equations (44)–(46), the recursion formulas for the power series coefficients *b_i_*, *c_i,_* and *d_i_* can be determined, where the coefficients *b_i_*, *c_i_* and *d_i_* (*i* = 1, 2, 3,…) can be expressed into the polynomials with respect to the coefficients *b*_0_ and *c*_0_, as shown in Appendix A.

The coefficients *b*_0_ and *c*_0_ are usually called undetermined constants and can be determined by using the boundary conditions Equations (48) and (49). By substituting Equations (41) and (42) into Equations (48) and (49), it is found that

(50) $$\sum_{i=0}^{\infty }c_{i}{\left(1-\beta \right)}^{i}-v\sum_{i=0}^{\infty }b_{i}{\left(1-\beta \right)}^{i}=0,$$

and

(51) $$\sum_{i=0}^{\infty }b_{i}{\left(\beta -1\right)}^{i}=\sum_{i=0}^{\infty }c_{i}{\left(\beta -1\right)}^{i}.$$

Since the coefficients *b_i_* and *c_i_* (*i* = 1, 2, 3,…) can be expressed into the polynomials with respect to the coefficients *b*_0_ and *c*_0_ (see Appendix A), the undetermined constants *b*_0_ and *c*_0_ can be determined by simultaneously solving Equations (50) and (51). The remaining coefficient *d*_0_ is a dependent undetermined constant that depends on the undetermined constants *b*_0_ and *c*_0_, and it can be determined by using the boundary condition Equation (47). By substituting Equation (43) into Equation (47), it is found that

(52) $$d_{0}=-\sum_{i=1}^{\infty }d_{i}{\left(1-\beta \right)}^{i}.$$

Since the coefficients *d_i_* (*i* = 1, 2, 3,…) can be expressed into the polynomials with respect to the coefficients *b*_0_ and *c*_0_ (see Appendix A), with the known undetermined constants *b*_0_ and *c*_0_, the undetermined constant *d*_0_ can be determined by Equation (52).

Therefore, for a concrete problem in which the values of the parameter variables *a*, *b*, *h*, *E*, *ν,* and *F* are known beforehand, the undetermined constants *b*_0_, *c*_0,_ and *d*_0_ can be determined by Equations (50)–(52), all the coefficients *b_i_*, *c_i_*, and *d_i_* (*i* = 1, 2, 3,…) can be determined, and all the expressions of *S_r_*(*x*), *S_t_*(*x*) and *W*(*x*) can also be determined. The large deflection problem within *b* ≤ *r* ≤ *a* can thus be solved analytically. Further, with the known *S_r_*(*x*) or *S_t_*(*x*), *u_B_* can be determined by Equation (38) or (39), the radial horizontal stretching problem within 0 ≤ *r* ≤ *b* can thus be solved analytically. The large deflection problem of axisymmetric deformation of the initially flat, peripherally fixed circular membrane subjected to the action of uniformly distributed axisymmetric line loads (i.e., the action of the cylindrical loading shaft in Figure 1) is thus solved analytically.

Finally, from Equations (32) and (41)–(43), the dimensional forms of the radial stress *σ_r_*, circumferential stress *σ_t,_* and transverse displacement (deflection) *w* within *b* ≤ *r* ≤ *a* can be written as

(53) $$\sigma_{r}(r)=E\sum_{i=0}^{\infty }b_{i}{\left(\frac{r}{a}-\frac{a+b}{2a}\right)}^{i},$$

(54) $$\sigma_{t}(r)=E\sum_{i=0}^{\infty }c_{i}{\left(\frac{r}{a}-\frac{a+b}{2a}\right)}^{i}$$

and

(55) $$w(r)=a\sum_{i=0}^{\infty }d_{i}{\left(\frac{r}{a}-\frac{a+b}{2a}\right)}^{i}.$$

As for the dimensional expressions of the radial strain *e_r_*, circumferential strain *e_t_*, and radial displacement *u*, with the known expressions of *σ_r_* and *σ_t_*, they can easily be derived from Equations (10) and (13). It is not necessary to derive them here.

### 2.2. Simultaneous Determination of Poisson’s Ratios and Young’s Modulus of Elasticity

As mentioned earlier, as long as the analytical solution for the elastic behavior of the steady shaft-loaded blistering thin film in Figure 1b is available, the Poisson’s ratio *v* and Young’s modulus of elasticity *E* of the surface thin film of the thin-film/substrate system specimen can be simultaneously determined by measuring the distance *L* of the screw rod being moved, the steady delamination radius *a*, the steady maximum thin-film deflection *w*_m_ at *r* = *b*, and the steady thin-film deflection *w*_(*a*+*b*)/2_ at *r* = (*a* + *b*)/2; see Figure 1 and Figure 2. The basic principle of the simultaneous determination of Poisson’s ratio *v* and Young’s modulus of elasticity *E* is detailed as follows.

It can be seen from Section 2.1 that six parameter variables (*a*, *b*, *h*, *E*, *ν*, and *F*) and three undetermined constants (*b*_0_, *c*_0,_ and *d*_0_) are involved during the derivation of the analytical solution. In which, the radius *b* of the cylindrical loading shaft and the thickness *h* of the surface thin film of the thin-film/substrate system specimen are two parameter variables as known, the steady delamination radius *a* of the steady shaft-loaded blistering thin-film can be determined by measurement, the spring force *F* acting on the steady shaft-loaded blistering thin film can be determined by *k_x_*(*L*–*w_m_*) (where the linear elastic stiffness coefficient *k_x_* of the spring is a parameter variable as known, the distance *L* of the screw rod being moved can be determined by measurement, the steady maximum thin film deflection *w_m_* at *r* = *b* can also be determined by measurement), and the undetermined constant *d*_0_ can be determined from Equation (55) by the measured value of the steady thin-film deflection *w*_(*a*+*b*)/2_ at *r* = (*a* + *b*)/2, i.e., *w*_(*a*+*b*)/2_ = *ad*_0_.

The simultaneous determination of the remaining two parameter variables *ν* and *E* and two undetermined constants *b*_0_ and *c*_0_ requires four equations containing only *ν*, *E*, *b*_0,_ and *c*_0_. Obviously, Equations (50)–(52) are the three available equations, since they exactly contain only *ν*, *E*, *b*_0,_ and *c*_0_, see Equation (32) and the recursion formulas for the power series coefficients *b_i_*, *c_i,_* and *d_i_* in Appendix A. So, we still need another equation containing only *ν*, *E*, *b*_0,_ and *c*_0_. This equation can be achieved from Equation (55) by the measured value of the steady maximum thin-film deflection *w_m_* at *r* = *b*, i.e.,

(56) $$w_{m}=a\sum_{i=0}^{\infty }d_{i}{\left(\frac{b}{a}-\frac{a+b}{2a}\right)}^{i}=ad_{0}+a\sum_{i=1}^{\infty }d_{i}{\left(\frac{b-a}{2a}\right)}^{i}=w_{(a+b)/2}+a\sum_{i=1}^{\infty }d_{i}{\left(\frac{b-a}{2a}\right)}^{i}.$$

In fact, for the shaft-loaded blister test illustrated in Figure 1, the radius *b* of the cylindrical loading shaft, the thickness *h* of the surface thin film of the thin-film/substrate system specimen, the distance *L* of the screw rod being moved, the delamination radius *a* of the steady shaft-loaded blistering thin film, the steady maximum thin-film deflection *w_m_* at *r* = *b*, and the steady thin-film deflection *w*_(*a*+*b*)/2_ at *r* = (*a* + *b*)/2 are parameters whose values can all be determined by measurement. The parameter variables *β* and *P*, which are contained in the power series coefficients *b_i_*, *c_i,_* and *d_i_* (*i* = 1, 2, 3, …, see Appendix A), are provided by *β* = (1 + *α*)/2 = (*a* + *b*)/*a/*2 and *P* = *F*/π/*a*/*h*/*E* = *k_x_*(*L* − *w_m_*)/π/*a*/*h*/*E*; see Equation (32). Therefore, in Equations (50)–(52) and (56), only *ν*, *E*, *b*_0,_ and *c*_0_ are unknown. This means that *ν*, *E*, *b*_0,_ and *c*_0_ can be determined by simultaneously solving Equations (50)–(52) and (56), which will be detailed in Section 3.2 with a demonstration experiment.

### 2.3. Determination of the Elastic Energy U_e_

It can be seen from Equation (2) that the energy release rate *G* can be determined as long as the elastic energy *U_e_* stored in the steady shaft-loaded blistering thin film in Figure 1 can be determined. However, the obtained analytical solution for the elastic behavior of the steady shaft-loaded blistering thin film in Figure 1 can, in fact, only be used indirectly, but not directly, to determine the elastic energy *U_e_*. The so-called indirect determination is detailed as follows.

If the steady shaft-loaded blistering thin film in Figure 1b (i.e., the shaft-loaded blistering thin film reaches static equilibrium under the shaft-loading force *F* = *k_x_*(*L* − *w_m_*) and produces the steady delamination radius *a* and maximum deflection *w_m_*; see Figure 1b) is still linearly elastic, then, after unloading, it can return to its initially flat position before delamination. This is equivalent to the case where the initially flat, peripherally fixed circular membrane with radius *a* in Figure 2 can produce a maximum elastic deflection *w*_m_ when it is subjected to the shaft-loading force *F* = *k_x_*(*L* − *w_m_*). Suppose that a shaft-loading dynamic force *F*, which varies from zero to *k_x_*(*L* − *w_m_*), is applied onto the initially flat, peripherally fixed circular membrane with radius *a* in Figure 2 and makes the circular membrane eventually produce a maximum elastic deflection *w_m_*, and that the work done by the dynamic force *F* to the circular membrane is all converted into the elastic energy that is stored in the deflected circular membrane. Then, the elastic energy *U_e_* stored in the steady shaft-loaded blistering thin film in Figure 1b can be calculated by

(57) $$U_{e}=\int_{0}^{w_{m}}F(w_{m})dw_{m},$$

where *F*(*w_m_*) is the load-deflection analytical relationship between the shaft-loading dynamic force *F* as a dependent variable and the maximum thin-film deflection *w_m_* as an independent variable; see Figure 2.

It can be seen from Equation (55) or (56) that the shaft-loading force *F* is contained in the power series coefficients *d_i_* (*i* = 1, 2, 3, …); see Equation (32) and Appendix A. So, Equation (55) or (56) is in fact the deflection-load analytical relationship *w_m_*(*F*) between the maximum thin-film deflection *w_m_* as a dependent variable and the shaft-loading dynamic force *F* as an independent variable. Obviously, due to the complicated power series coefficients *d_i_* (*i* = 1, 2, 3,…), the desired load-deflection analytical relationship *F*(*w_m_*) in Equation (57) cannot be derived directly from Equation (55) or (56). But it can be obtained by least square data fitting based on a large number of numerical calculations, which will be detailed in Section 3.3 with a numerical example. After the load-deflection analytical relationship *F*(*w_m_*) is available, the elastic energy *U_e_* stored in the steady shaft-loaded blistering thin film in Figure 1b can easily be determined by Equation (57), and the energy release rate *G* can thus be determined with Equation (2).

### 2.4. Operation Process of Experiment and Synchronous Characterization

The steps of the experimental operation and synchronous characterization can be summarized as follows:

(1) Prepare a thin-film/substrate system specimen, drill (or chemically etch) a small circular hole with radius *d* on the substrate of the specimen, and make the surface thin film within radius *d* fully exposed and suspended, as shown in Figure 1a;
(2) Slowly rotate the screw rod until it has moved a displacement *L* and the delamination radius *a* of the shaft-loaded blistering thin film has been exceeded by about two times *d*, as shown in Figure 1b;
(3) Observe the shaft-loaded blistering thin film until it reaches static equilibrium (steady, no change in size);
(4) Measure the displacement *L* by which the screw rod is moved, the steady delamination radius *a*, and the steady thin-film deflection *w_m_* and *w*_(*a*+*b*)/2_ (see Figure 1b) of the steady shaft-loaded blistering thin film, where the displacement *L* can be measured with a micrometer screw, the deflection *w*_m_ and *w*_(*a*+*b*)/2_ can be measured with a laser displacement sensor (see Figure 5 for details), and the delamination radius *a* is exactly located at the point where the thin-film deflection is zero and thus can be measured with the laser displacement sensor and micrometer screw;
(5) Slowly rotate the screw in the opposite direction until the displacement *L* just disappears completely (unloading);
(6) Observe whether the steady shaft-loaded blistering thin film can completely return to its position of no delamination after unloading. If it can, measure the thin-film thickness *h* and the radius *b* of the cylindrical loading shaft. Otherwise, go back to Step 1 and repeat Step 1–Step 6 until it can;
(7) Measure the radius *d* of the small circular hole on the substrate, the thin-film thickness *h,* and the radius *b* of the cylindrical loading shaft;
(8) Analytically solve the elastic behavior of the steady shaft-loaded blistering thin film (whose steady delamination radius reaches *a*, and steady maximum thin-film deflection reaches *w_m_* when the shaft-loading spring force *F* reaches *k_x_*(*L*−*w_m_*); see Figure 1b and Figure 2);
(9) Use Equation (1) and the measured values of the displacement *L* and the steady thin-film deflection *w_m_* to calculate the work *U_F_* done by the shaft-loading spring force *F* to the delamination system;
(10) Use Equations (50)–(52) and (56) and the measured values of the steady delamination radius *a*, the steady thin-film deflection *w_m_* and *w*_(*a*+*b*)/2_, the thin-film thickness *h*, and the radius *b* of the cylindrical loading shaft to simultaneously determine the Poisson’s ratio *v* and Young’s modulus of elasticity *E* of the surface thin film of the thin-film/substrate system specimen;
(11) Use Equation (57) to calculate the elastic energy *U_e_* stored in the steady shaft-loaded blistering thin film;
(12) Use Equation (2) to calculate the interface energy release rate *G*.

It can be seen from the above statement that the so-called synchronous characterization for the surface and interface mechanical properties of a thin-film/substrate system specimen refers to the surface mechanical properties (the Poisson’s ratio *v* and Young’s modulus of elasticity *E* of the surface thin film of the thin-film/substrate system specimen) and the interface mechanical property (the interface energy release rate *G* of the thin-film/substrate system specimen) are simultaneously determined by using the measured data from one shaft-loaded blister test.

## 3. Results and Discussion

In this section, the work presented here is compared with the one previously presented in [67] to show the difference between the two works and the importance of the work presented here.

**Figure 4:**
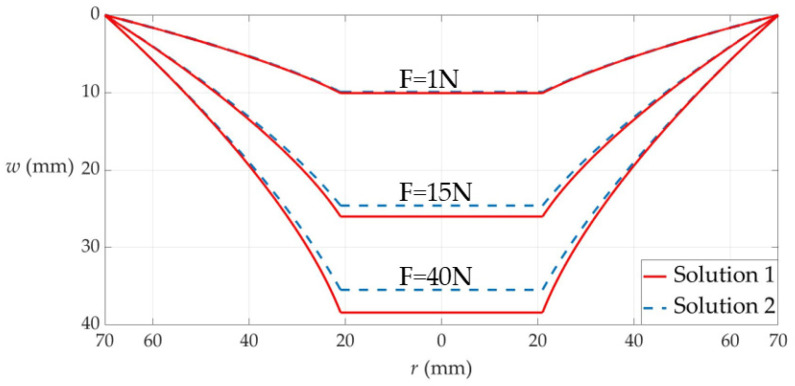
The variation of the membrane deflection *w* with the radial coordinate *r*, where “Solution 1” refers to the results calculated by the analytical solution newly presented here, and “Solution 2” refers to the results calculated by the analytical solution previously presented in [67].

A comparison is first provided in Section 3.1 between the previous and new analytical solutions for the elastic behavior of the steady shaft-loaded blistering thin film in Figure 1b. The comparison between the previous and new governing equations used during the derivation of the two analytical solutions is shown in Table 1, and the comparison between the deflection curves calculated by the previous and new analytical solutions is shown in Figure 4. The results show that the use of the new governing equations makes the difference between the previous and new analytical solutions increase with the increase of loading, thus greatly improving the previous analytical solution.

In Section 3.2, the previous and new synchronous characterization theories for surface and interface mechanical properties of thin-film/substrate systems are used for an experiment of simultaneously determining the Poisson’s ratio *v* and Young’s modulus of elasticity *E* of a free-standing natural latex circular thin-film specimen, where the well-established bulge test technique is also used. The result of the well-established bulge test technique agrees quite closely with the result of the new synchronous characterization theory, but it is quite different from the result of the previous synchronous characterization theory, suggesting that the new synchronous characterization theory is more reliable than the previous one and that the new analytical solution underpinning the new synchronous characterization theory is also more reliable than the previous analytical solution.

In Section 3.3, the elastic energy *U_e_* of the steady shaft-loaded blistering natural latex thin film in Section 3.2 is calculated by the previous and new synchronous characterization theories, and the relative error between the calculation results by the previous and new theories is about 15%, suggesting that the improvement work done in this paper has a positive effect and is necessary.

### 3.1. Comparison between New and Previous Analytical Solutions

The analytical solution for the elastic behavior of the steady shaft-loaded blistering thin film in Figure 1b, which is newly presented in Section 2.1 in this paper, differs from the analytical solution previously presented in [67], mainly in that the membrane governing equations used in [67] are derived under the assumption of the small rotation angle of the membrane, resulting in a limit to applications of larger membrane deflections. The differences between old and new governing equations are shown in Table 1.

It can be seen from Table 1 that with the exception of the circumferential geometric equation, the out-of-plane and in-plane equilibrium equations and radial geometric equation are all improved in this paper, and that the contribution of the deflection to the equilibrium and geometric relationships are fully considered here. Therefore, the new analytical solution can be adapted to applications with larger membrane deflections, in comparison with the previous analytical solution. Here, we consider a numerical example to show the difference between the membrane deflections calculated by the previous and new analytical solutions, as shown in Figure 4, where a circular film with radius *a* = 70 mm, thickness *h* = 0.8 mm, Poisson’s ratio *v* = 0.431613, and Young’s modulus of elasticity *E* = 0.939645 MPa is subjected to the action of a cylindrical loading shaft with the radius *b* = 38 mm. The shaft-loading force *F* varies from 1 N to 15 N and then 40 N, “Solution 1” refers to the results calculated by the new analytical solution presented here, and “Solution 2” refers to the results calculated by the previous analytical solution presented in [67]. It can be seen from Figure 4 that the two solutions agree quite closely when the shaft-loading force *F* is equal to 1N and diverges as the shaft-loading force *F* intensifies. The increasing deflection difference with loading in Figure 4 suggests that the improved out-of-plane and in-plane equilibrium and radial geometric equations, used in this paper, play a positive improvement role.

### 3.2. A Demonstration Experiment for Simultaneously Determining v and E

It can be seen from Section 2.4 that the synchronous characterization based on the shaft-loaded blister test in Figure 1 is actually to first determine the Poisson’s ratio *v* and Young’s modulus of elasticity *E* of the surface thin film (the surface mechanical properties of the thin-film/substrate system specimen) synchronously. Then, use the determined *v* and *E* to further determine the elastic energy *U_e_* and, finally, determine the interface mechanical property of the thin-film/substrate system specimen, i.e., the energy-release rate *G*. Therefore, the basic principle of the synchronous determination of Poisson’s ratio *v* and Young’s modulus of elasticity *E* by the shaft-loaded blister test in Figure 1 is the same as that of the simultaneous determination of Poisson’s ratio *v* and Young’s modulus of elasticity *E* by bulge test technique [70]. So, an initially flat, peripherally fixed, shaft-loaded circular membrane (as shown in Figure 2) is used here to simulate the steady shaft-loaded blistering thin film with the steady delamination radius *a* and thin-film deflections *w*_m_ and *w*_(*a*+*b*)/2_ (see Figure 1b) to conduct an experiment demonstrating the simultaneous determination of Poisson’s ratio *v* and Young’s modulus of elasticity *E*. And the natural latex circular thin film with radius *a* = 70 mm and thickness *h* = 0.8 mm used in the bulge test in [71] (see Figure 10 in [71]) is used to conduct such a demonstration experiment so as to make a comparison between the results by the shaft-loaded blister test technique and by the bulge test technique.

**Figure 5:**
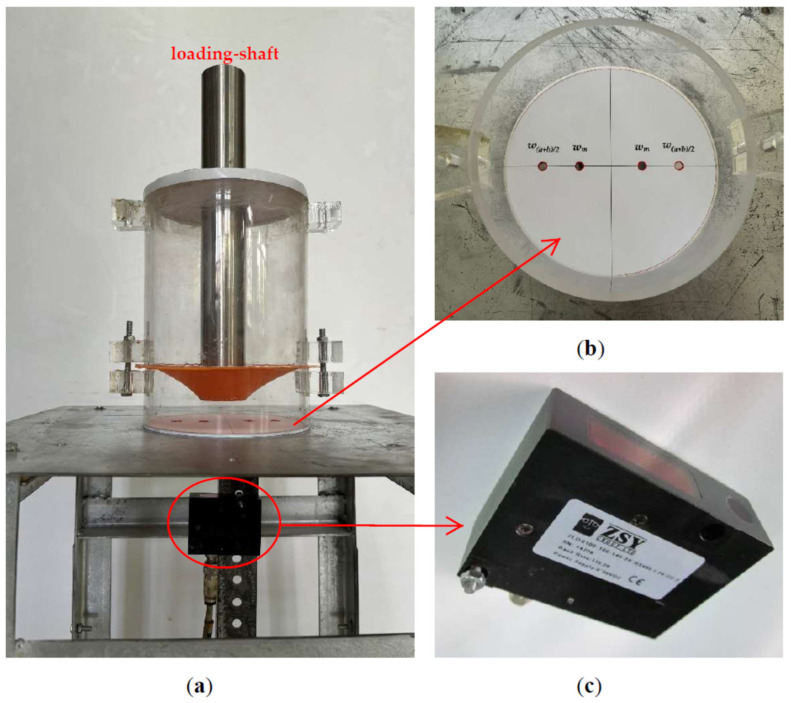
The shaft-loaded experiment of the natural latex circular thin film (used in the bulge test in [71], see Figure 10 in [71]) with radius *a* = 70 mm and thickness *h* = 0.8 mm: (**a**) the experimental setup; (**b**) a detailed view of the points for measuring the thin-film deflections *w_m_* and *w*_(*a*+*b*)/2_; and (**c**) a detailed view of the laser-displacement sensor (ZSY Group Ltd., London, UK) used for measuring *w_m_* and *w*_(*a*+*b*)/2_.

As shown in Figure 5, the natural latex circular thin film with radius *a* = 70 mm and thickness *h* = 0.8 mm is initially flat and fixed at its perimeter of radius *a* = 70 mm. It is then loaded by a cylindrical loading shaft with radius *b* = 21 mm and weight 3.3 kg (*F* = 3.3 × 9.8 = 32.34 N) and, finally, becomes the initially flat, peripherally fixed, shaft-loaded circular membrane with the deflections *w_m_* at *r* = *b* and *w*_(*a*+*b*)/2_ at *r* = (*a* + *b*)/2 (as shown in Figure 2). The thin-film deflections measured at *r* = *b* = 21 mm and at *r* = (*a* + *b*)/2 = (70 + 21)/2 = 45.5 mm are *w_m_* = 35.013 mm and *w*_(*a*+*b*)/2_ = 14.986 mm, respectively. Poisson’s ratio *v* and Young’s modulus of elasticity *E* of the used natural latex thin film can be synchronously determined by either the improved synchronous characterization theory here or the previous synchronous characterization theory presented in [67]. But the two synchronous characterization theories before and after improvement are both used here in order to make a comparison between the two.

The use of the improved synchronous characterization theory is detailed as follows. From the measured value of *w*_(*a*+*b*)/2_ =14.986 mm at *r* = (*a* + *b*)/2 = 45.5 mm, Equation (55) provides *d*_0_ = *w*_(*a*+*b*)/2_/*a* = 14.986 mm/70 mm = 0.214086. Therefore, after considering the measured value of *w_m_* = 35.013 mm at *r* = 21 mm, it can be found from Equations (50)–(52) and (56) that

(58) $$\sum_{i=0}^{\infty }c_{i}{\left(1-0.65\right)}^{i}-v\sum_{i=0}^{\infty }b_{i}{\left(1-0.65\right)}^{i}=0,$$

(59) $$\sum_{i=0}^{\infty }b_{i}{\left(0.65-1\right)}^{i}-\sum_{i=0}^{\infty }c_{i}{\left(0.65-1\right)}^{i}=0,$$

(60) $$0.214086+\sum_{i=1}^{\infty }d_{i}{\left(1-0.65\right)}^{i}=0$$

and

(61) $$\frac{35.013\;\mathrm{mm}-14.986\;\mathrm{mm}}{70\;\mathrm{mm}}-\sum_{i=1}^{\infty }d_{i}{\left(\frac{21\;\mathrm{mm}-70\;\mathrm{mm}}{2\times 70\;\mathrm{mm}}\right)}^{i}=0,$$

where the power-series coefficients *b_i_*, *c_i_*, and *d_i_* (*i* = 1, 2, 3,…) contain only *ν*, *E*, *b*_0_, and *c*_0_, in addition to the parameter variables *β* and *P* (where *β* = (1 + *α*)/2 = (1 + *b*/*a*)/2 = (1 + 21 mm/70 mm)/2 = 0.65) and *P* = *F*/(π*ahE*) (see Equation (32)), *F* = 32.34 N, *a* = 70 mm, and *h* = 0.8 mm). Therefore, it can be found from the simultaneous solution of Equations (58)–(61) that *b*_0_ = 0.268982, *c*_0_ = 0.0971923, *v* = 0.431613, and *E* = 0.939645 MPa.

The use of the synchronous characterization theory previously presented in [67] is detailed as follows. Since *w_m_*/*w*_(*a*+*b*)/2_ = 35.013 mm/14.986 mm = 2.33638 and *α* = *b*/*a* = 21 mm/70 mm = 0.3, then it can be found from Figure 11 in [67] (the variation of *w_m_*/*w*_(*a*+*b*)/2_ with *v* when *α* takes different values) that Poisson’s ratio *v* should be equal to 0.405. Further, it can be found from Figure 6 in [67] (the variation of *w_m_*/(2*P*)^1/3^ with *v* when *α* takes different values) that *W_m_*/(2*P*)^1/3^ should be equal to 1.07 when v = 0.4 and *α* = 0.3, where *P* is the dimensionless form of the force *F* and is provided by *P* = *a*^2^*F*/(4π*h*^4^*E*); see Equation (16) in [67]. Therefore, it can be found from *W_m_*/(2*P*)^1/3^ = 1.06, *W_m_* = *w_m_*/*h*, *P* = *a*^2^*F*/(4π*h*^4^*E*), *w_m_* = 35.013 mm, *F* = 32.34 N, *a* = 70 mm, and *h* = 0.8 mm that the Young’s modulus of elasticity *E* is equal to 0.874774 MPa.

On the other hand, this natural latex circular thin film with radius *a* = 70 mm and thickness *h* = 0.8 mm has already been used in the bulge test in [71], where the natural latex circular thin film is loaded by a gas pressure of *q* = 0.008 MPa, the thin-film deflections *w_r_*_=0_ at *r* = 0 mm and *w_r_*_=30_ at *r* = 40 mm are measured to be *w_r_*_=0_ = 39.52 mm and *w_r_*_=40_ = 31.49 mm, respectively; see Figure 10 and Table 3 in [71] for details. So far, there have been three analytical solutions for the large deflection problem of the initially flat, peripherally fixed, gas-pressure-loaded circular membranes [71,72,73,74], among which the analytical solution in [74] is the latest and most accurate one. Therefore, the analytical solution in [74] is used here to analyze (explain) the large deflection elastic behavior of the natural latex thin-film circular thin film with radius *a* = 70 mm and thickness *h* = 0.8 mm in this bulge test so as to synchronously determine its Poisson’s ratio *v* and Young’s modulus of elasticity *E*, which is detailed as follows. From the measured value of *w_r_*_=0_ = 39.52 mm at *r* = 0 mm, Equation (35) in [74] provides *d*_0_ = *w_r_*_=0_/*a* = 39.52 mm/70 mm = 0.564571. Therefore, after considering the measured value of *w_r_*_=40_ = 31.49 mm at *r* = 40 mm, it can be found from Equations (31), (32), and (35) in [74] that

(62) $$\sum_{i=0}^{\infty }c_{i}-v\sum_{i=0}^{\infty }b_{i}=0,$$

(63) $$\frac{39.52\;\mathrm{mm}}{70\;\mathrm{mm}}+\sum_{i=1}^{\infty }d_{i}=0$$

and

(64) $$\frac{31.49\;\mathrm{mm}-39.52\;\mathrm{mm}}{70\;\mathrm{mm}}-\sum_{i=1}^{\infty }d_{i}{\left(\frac{40\;\mathrm{mm}}{70\;\mathrm{mm}}\right)}^{i}=0,$$

where the power-series coefficients *b_i_*, *c_i_*, and *d_i_* (*i* = 2, 4, 6,…) contain only *ν*, *E*, and *b*_0_ (*c*_0_ ≡ *b*_0_, see Appendix A in [74]), in addition to the dimensionless variables *Q* (*Q* is the dimensionless form of the gas pressure *q*, *Q* = *aq*/*h/E*, see Equation (21) in [74], *q* = 0.008 MPa, *a* = 70 mm, and *h* = 0.8 mm). Therefore, it can be found from the simultaneous solution of Equations (62)–(64) that *b*_0_ = 0.598028, *v* = 0.431947, and *E* = 0.940735 MPa.

The results of the above three calculations are summarized in Table 2, showing that the values of Poisson’s ratio *v* and Young’s modulus of elasticity *E*, which are determined by the bulge test technique and the shaft-loaded blister test technique using the improved synchronous characterization theory, agree quite closely, while the *v* and *E* values determined by the shaft-loaded blister test technique using the improved synchronous characterization theory significantly differ from the *v* and *E* values determined by the shaft-loaded blister test technique using the synchronous characterization theory previously presented in [67]. This suggests that the improved out-of-plane and in-plane equilibrium and radial geometric equations play a positive improvement role in the improved synchronous characterization theory.

### 3.3. Calculation of the Elastic Energy U_e_ of Shaft-Loaded Blistering Thin-Film

According to the operation process of experiment and synchronous characterization in Section 2.4, once the Poisson’s ratio *v* and Young’s modulus of elasticity *E* of the steady shaft-loaded blistering thin film are determined, the elastic energy *U_e_* stored in this steady shaft-loaded blistering thin film should be calculated so as to further determine the energy-release rate *G* in the next step. As described in Section 2.3, the elastic energy *U_e_* can be calculated using Equation (57) as long as the load-deflection analytical relationship *F*(*w_m_*) between the shaft-loading dynamic force *F* as a dependent variable and the maximum thin-film deflection *w_m_* as an independent variable is available. However, the load-deflection analytical relationship *F*(*w_m_*) cannot be derived directly from Equation (55) or (56), because the shaft-loading force *F* is contained in the complicated power series coefficients *d_i_* (*i* = 1, 2, 3, …) of Equation (55) or (56) (see Equation (32) and Appendix A). Therefore, the required load-deflection analytical relationship *F*(*w_m_*) can only be obtained by least square data fitting based on a large number of numerical calculations, which are detailed as follows.

**Figure 6:**
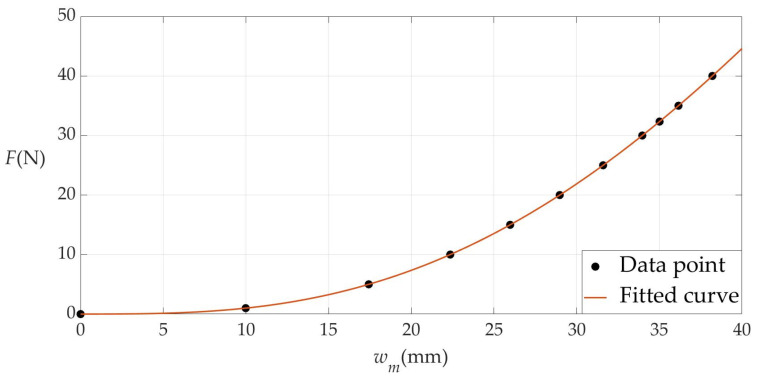
The load-deflection analytical relationship *F*(*w_m_*) between the shaft-loading force *F* and maximum deflection *w_m_*.

As described in Section 3.2, the steady shaft-loaded blistering thin film with the steady delamination radius *a* = 70 mm, thickness *h* = 0.8 mm, Poisson’s ratio *v* = 0.431613, and Young’s modulus of elasticity *E* = 0.939645 MPa reaches static equilibrium under the shaft-loading force *F* = 32.34 N (the radius of the cylindrical loading-shaft is *b* = 21 mm) and produces the steady maximum deflection *w_m_* = 35.013 mm. Therefore, the shaft-loading dynamic force *F* needs to take a series of different values in the interval [0, 40 N] for numerical calculations, as listed in Table 3. The numerical values of the shaft-loading dynamic force *F* and maximum deflection *w_m_* in Table 3 are used for the least square data fitting, where the maximum deflection *w_m_* is used as an independent variable, and the shaft-loading dynamic force *F* is used as a dependent variable, as shown in Figure 6.

The fitted analytical function for the load-deflection analytical relationship *F*(*w_m_*) is provided by

(65) $$\begin{array}{cc} F= & -5.0448\times 10^{-12}w_{m}^{7}+8.3501\times 10^{-10}w_{m}^{6}-3.9148\times 10^{-9}w_{m}^{5}-1.4965\times 10^{-5}w_{m}^{4}\\ & +1.3187\times 10^{-3}w_{m}^{3}-2.5133\times 10^{-3}w_{m}^{2}+8.6333\times 10^{-3}w_{m}-1.2032\times 10^{-8} \end{array}.$$

Therefore, from Equations (57) and (58), the elastic energy *U_e_* stored in the steady shaft-loaded blistering thin film can be calculated to be

(66) $$\begin{array}{cc} U_{e}=\int_{0}^{35.013} & (-5.0448\times 10^{-12}w_{m}^{7}+8.3501\times 10^{-10}w_{m}^{6}-3.9148\times 10^{-9}w_{m}^{5}\\ & -1.4965\times 10^{-5}w_{m}^{4}+1.3187\times 10^{-3}w_{m}^{3}-2.5133\times 10^{-3}w_{m}^{2}\\ & +8.6333\times 10^{-3}w_{m}-1.2032\times 10^{-8})dw_{m}\\ & =0.312365\;(J) \end{array}.$$

On the other hand, the synchronous characterization theory previously presented in [67] can also be used to determine the elastic energy *U_e_* stored in this steady shaft-loaded blistering thin film. It can be found from Equation (28) in [67] that since (1 + *v*)[3 − (*b*/*a*)^2^] = (1 + 0.405)[3 − (21 mm/70 mm)^2^] = 4.08855 > 4, then *B* < 0. Further, from *v* = 0.405, *α* = *b*/*a* = 21 mm/70 mm = 0.3, and the simultaneous solution of Equations (A52) and (A54) in [67], it can be found that *φ*_1_ = 1.25 and *φ_α_* = 1.441148. Therefore, from *w_m_* = 35.013 mm, *a* = 70 mm, *b* = 21 mm, *h* = 0.8 mm, *α* = *b*/*a* = 0.3, *v* = 0.405, *E* = 0.874774 MPa, *φ*_1_ = 1.25, and *φ_α_* = 1.441148, the elastic energy *U_e_* stored in this steady shaft-loaded blistering thin film can be calculated directly by Equation (36) in [67] and is equal to 0.271556 (J).

The relative error between the result 0.312365 J calculated by the improved theory here and the result 0.271556 J calculated by the previous theory in [67] is about 15%, and this 15% relative error will undoubtedly affect the accurate determination of the interface energy release rate *G*; see Equation (2).

## 4. Concluding Remarks

In this paper, the large deflection problem of the steady shaft-loaded blistering thin film in the previously proposed shaft-loaded blister test technique is reformulated, where the out-of-plane and in-plane equilibrium and radial geometric equations, which are previously established under the small-rotation-angle assumption of the membrane, are replaced by the new equations without the small-rotation-angle assumption of the membrane. The resulting new and more accurate analytical solution of the large deflection problem is used to improve the previously presented synchronous characterization theory for the surface and interface mechanical properties of thin-film/substrate systems. The new and previous analytical solutions are numerically compared. An experiment is conducted to compare the well-established bulge test technique with the new and previous synchronous characterization theories (for determining Poisson’s ratio *v* and Young’s modulus of elasticity *E*) and compare the new synchronous characterization theory with the previous synchronous characterization theory (for determining the elastic energy *U_e_* stored in the steady shaft-loaded blistering thin film). From this study, the following conclusions can be drawn.

Due to the use of the new out-of-plane and in-plane equilibrium and radial geometric equations, the new analytical solution has a clear superiority over the previous analytical solution in adapting to applications with larger deflections (or larger rotation angle of membrane).

Due to the improvement by the new and more accurate analytical solution, the new synchronous characterization theory can simultaneously determine the values of Poisson’s ratio *v*, Young’s modulus of elasticity *E*, and elastic energy *U_e_* of the steady shaft-loaded blistering thin-film more accurately, in comparison with the previous synchronous characterization theory.

However, due to the limitation of experimental conditions, we failed to conduct the delamination experiment as shown in Figure 1 to achieve the synchronous characterization for the surface and interface mechanical properties of a thin-film/substrate system specimen. So, further research is still necessary, which needs to develop and fabricate the delamination experimental devices as shown in Figure 1.

## Figures and Tables

**Table 1:** Comparison between the new and previous governing equations.

	Out-of-Plane Equilibrium Equation	In-Plane Equilibrium Equation	Geometric Equation
New:	$F-\frac{2\pi r\sigma_{r}h}{\sqrt{1+1/{\left(-dw/dr\right)}^{2}}}=0$	$\frac{d}{dr}(\frac{r\sigma_{r}}{\sqrt{1+{\left(-dw/dr\right)}^{2}}})-\sigma_{t}\sqrt{1+{\left(-dw/dr\right)}^{2}}=0$	$\left\{\begin{array}{l} e_{r}=\sqrt{{\left(1+\frac{du}{dr}\right)}^{2}+{\left(\frac{dw}{dr}\right)}^{2}}-1\\ e_{t}=\frac{u}{r} \end{array}\right.$
Previous:	$F+2\pi rh\sigma_{r}\frac{dw}{dr}=0$	$\frac{d}{dr}(rh\sigma_{r})-h\sigma_{t}=0$	$\left\{\begin{array}{l} e_{r}=\frac{du}{dr}+\frac{1}{2}{\left(\frac{dw}{dr}\right)}^{2}\\ e_{t}=\frac{u}{r} \end{array}\right.$

**Table 2:** The calculation results of Poisson’s ratio *v* and Young’s modulus of elasticity *E*.

	Bulge Test Technique	Shaft-Loaded Blister Test Technique	
		Improved Theory	Previous Theory
***v*:**	0.431947	0.431613	0.405
***E*:**	0.940735 MPa	0.939645 MPa	0.874774 MPa

**Table 3:** The results of numerical calculation when the shaft-loading force *F* takes different values.

*F*(N)	*b_0_*	*c_0_*	*d_0_*	*w_m_* (mm)
0	0	0	0	0
1	0.0227810	0.0047836	0.0644515	9.9849
5	0.0684675	0.0162326	0.1110413	17.4260
10	0.1112971	0.0284219	0.1408425	22.3550
15	0.1486694	0.0396269	0.1621667	25.9771
20	0.1830992	0.0500740	0.1794796	28.9759
25	0.2155924	0.0598293	0.1944061	31.5994
30	0.2466721	0.0689271	0.2077465	33.9696
32.34	0.2608252	0.0724748	0.2140860	35.0130
35	0.2766521	0.0773970	0.2199606	36.1573
40	0.3057394	0.0852687	0.2313390	38.2074

## Data Availability

The original contributions presented in the study are included in the article, further inquiries can be directed to the corresponding author.

## Appendix A

$$d_{1}=-\frac{P}{\sqrt{4\beta^{2}b_{0}^{2}-P^{2}}}$$

$$d_{2}=\frac{2\beta b_{0}P\sqrt{4\beta^{2}b_{0}^{2}-P^{2}}c_{0}}{16\beta^{4}b_{0}^{4}-8P^{2}\beta^{2}b_{0}^{2}+P^{4}}$$

$$\begin{array}{l} d_{3}=-\frac{2}{3d_{1}(-8\beta^{2}b_{0}^{2}d_{1}^{2}+P^{2}d_{1}^{2}-4\beta^{2}b_{0}^{2}+P^{2})}(-8\beta b_{0}c_{0}d_{1}^{5}d_{2}-\beta b_{0}c_{1}d_{1}^{6}-8\beta^{2}b_{0}^{2}d_{1}^{2}d_{2}^{2}\\ -6\beta^{2}b_{0}b_{1}d_{1}^{3}d_{2}-\beta^{2}b_{1}^{2}d_{1}^{4}-6\beta b_{0}^{2}d_{1}^{3}d_{2}-2\beta b_{0}b_{1}d_{1}^{4}-8\beta b_{0}c_{0}d_{1}^{3}d_{2}-2\beta b_{0}c_{1}d_{1}^{4}+P^{2}d_{1}^{2}d_{2}^{2}\\ -4\beta^{2}b_{0}^{2}d_{2}^{2}-8\beta^{2}b_{0}b_{1}d_{1}d_{2}-\beta^{2}b_{1}^{2}d_{1}^{2}-b_{0}^{2}d_{1}^{4}-8\beta b_{0}^{2}d_{1}d_{2}-2\beta b_{0}b_{1}d_{1}^{2}-\beta b_{0}c_{1}d_{1}^{2}+P^{2}d_{2}^{2}-b_{0}^{2}d_{1}^{2}) \end{array}$$

$$\begin{array}{l} d_{4}=-\frac{1}{6d_{1}(-8\beta^{2}b_{0}^{2}d_{1}^{2}+P^{2}d_{1}^{2}-4\beta^{2}b_{0}^{2}+P^{2})}(-24\beta b_{0}c_{0}d_{1}^{5}d_{3}-48\beta b_{0}c_{0}d_{1}^{4}d_{2}^{2}-16\beta b_{0}c_{1}d_{1}^{5}d_{2}\\ -2\beta b_{0}c_{2}d_{1}^{6}-72\beta^{2}b_{0}^{2}d_{1}^{2}d_{2}d_{3}-36\beta^{2}b_{0}b_{1}d_{1}^{3}d_{3}-24\beta^{2}b_{0}b_{1}d_{1}^{2}d_{2}^{2}-12\beta^{2}b_{0}b_{2}d_{1}^{3}d_{2}-12\beta^{2}b_{1}^{2}d_{1}^{3}d_{2}\\ -6\beta^{2}b_{1}b_{2}d_{1}^{4}-36\beta b_{0}^{2}d_{1}^{3}d_{3}-24\beta b_{0}^{2}d_{1}^{2}d_{2}^{2}-36\beta b_{0}b_{1}d_{1}^{3}d_{2}-6\beta b_{0}b_{2}d_{1}^{4}-24\beta b_{0}c_{0}d_{1}^{3}d_{3}\\ -16\beta b_{0}c_{0}d_{1}^{2}d_{2}^{2}-16\beta b_{0}c_{1}d_{1}^{3}d_{2}-4\beta b_{0}c_{2}d_{1}^{4}-6\beta b_{1}^{2}d_{1}^{4}+9P^{2}d_{1}^{2}d_{2}d_{3}-36\beta^{2}b_{0}^{2}d_{2}d_{3}\\ -36\beta^{2}b_{0}b_{1}d_{1}d_{3}-24\beta^{2}b_{0}b_{1}d_{2}^{2}-24\beta^{2}b_{0}b_{2}d_{1}d_{2}-12\beta^{2}b_{1}^{2}d_{1}d_{2}-6\beta^{2}b_{1}b_{2}d_{1}^{2}-12b_{0}^{2}d_{1}^{3}d_{2}\\ -6b_{0}b_{1}d_{1}^{4}-36\beta b_{0}^{2}d_{1}d_{3}-24\beta b_{0}^{2}d_{2}^{2}-48\beta b_{0}b_{1}d_{1}d_{2}-6\beta b_{0}b_{2}d_{1}^{2}-2\beta b_{0}c_{2}d_{1}^{2}-6\beta b_{1}^{2}d_{1}^{2}\\ +9P^{2}d_{2}d_{3}-12b_{0}^{2}d_{1}d_{2}-6b_{0}b_{1}d_{1}^{2}) \end{array}$$

$$\begin{array}{l} d_{5}=-\frac{1}{10d_{1}(-8\beta^{2}b_{0}^{2}d_{1}^{2}+P^{2}d_{1}^{2}-4\beta^{2}b_{0}^{2}+P^{2})}(-32\beta b_{0}c_{0}d_{1}^{5}d_{4}-144\beta b_{0}c_{0}d_{1}^{4}d_{2}d_{3}-64\beta b_{0}c_{0}d_{1}^{3}d_{2}^{3}\\ -24\beta b_{0}c_{1}d_{1}^{5}d_{3}-48\beta b_{0}c_{1}d_{1}^{4}d_{2}^{2}-16\beta b_{0}c_{2}d_{1}^{5}d_{2}-2\beta b_{0}c_{3}d_{1}^{6}-128\beta^{2}b_{0}^{2}d_{1}^{2}d_{2}d_{4}-72\beta^{2}b_{0}^{2}d_{1}^{2}d_{3}^{2}\\ -72\beta^{2}b_{0}b_{1}d_{1}^{3}d_{4}-108\beta^{2}b_{0}b_{1}d_{1}^{2}d_{2}d_{3}-36\beta^{2}b_{0}b_{2}d_{1}^{3}d_{3}-24\beta^{2}b_{0}b_{2}d_{1}^{2}d_{2}^{2}-12\beta^{2}b_{0}b_{3}d_{1}^{3}d_{2}\\ -24\beta^{2}b_{1}^{2}d_{1}^{3}d_{3}-16\beta^{2}b_{1}^{2}d_{1}^{2}d_{2}^{2}-32\beta^{2}b_{1}b_{2}d_{1}^{3}d_{2}-8\beta^{2}b_{1}b_{3}d_{1}^{4}-4\beta^{2}b_{2}^{2}d_{1}^{4}-72\beta b_{0}^{2}d_{1}^{3}d_{4}\\ -108\beta b_{0}^{2}d_{1}^{2}d_{2}d_{3}-84\beta b_{0}b_{1}d_{1}^{3}d_{3}-56\beta b_{0}b_{1}d_{1}^{2}d_{2}^{2}-44\beta b_{0}b_{2}d_{1}^{3}d_{2}-8\beta b_{0}b_{3}d_{1}^{4}-32\beta b_{0}c_{0}d_{1}^{3}d_{4}\\ -48\beta b_{0}c_{0}d_{1}^{2}d_{2}d_{3}-24\beta b_{0}c_{1}d_{1}^{3}d_{3}-16\beta b_{0}c_{1}d_{1}^{2}d_{2}^{2}-16\beta b_{0}c_{2}d_{1}^{3}d_{2}-4\beta b_{0}c_{3}d_{1}^{4}-32\beta b_{1}^{2}d_{1}^{3}d_{2}\\ -16\beta b_{1}b_{2}d_{1}^{4}+16P^{2}d_{1}^{2}d_{2}d_{4}+9P^{2}d_{1}^{2}d_{3}^{2}-64\beta^{2}b_{0}^{2}d_{2}d_{4}-36\beta^{2}b_{0}^{2}d_{3}^{2}-64\beta^{2}b_{0}b_{1}d_{1}d_{4}-4b_{1}^{2}d_{1}^{4}\\ -96\beta^{2}b_{0}b_{1}d_{2}d_{3}-48\beta^{2}b_{0}b_{2}d_{1}d_{3}-32\beta^{2}b_{0}b_{2}d_{2}^{2}-32\beta^{2}b_{0}b_{3}d_{1}d_{2}-24\beta^{2}b_{1}^{2}d_{1}d_{3}-16\beta^{2}b_{1}^{2}d_{2}^{2}\\ -32\beta^{2}b_{1}b_{2}d_{1}d_{2}-8\beta^{2}b_{1}b_{3}d_{1}^{2}-4\beta^{2}b_{2}^{2}d_{1}^{2}-24b_{0}^{2}d_{1}^{3}d_{3}-16b_{0}^{2}d_{1}^{2}d_{2}^{2}-32b_{0}b_{1}d_{1}^{3}d_{2}-8b_{0}b_{2}d_{1}^{4}\\ -64\beta b_{0}^{2}d_{1}d_{4}-96\beta b_{0}^{2}d_{2}d_{3}-96\beta b_{0}b_{1}d_{1}d_{3}-64\beta b_{0}b_{1}d_{2}^{2}-64\beta b_{0}b_{2}d_{1}d_{2}-8\beta b_{0}b_{3}d_{1}^{2}-2\beta b_{0}c_{3}d_{1}^{2}\\ -32\beta b_{1}^{2}d_{1}d_{2}-16\beta b_{1}b_{2}d_{1}^{2}+16P^{2}d_{2}d_{4}+9P^{2}d_{3}^{2}-24b_{0}^{2}d_{1}d_{3}-16b_{0}^{2}d_{2}^{2}-32b_{0}b_{1}d_{1}d_{2}-8b_{0}b_{2}d_{1}^{2}-4b_{1}^{2}d_{1}^{2}) \end{array}$$

$$\begin{array}{l} d_{6}=-\frac{1}{15d_{1}(-8\beta^{2}b_{0}^{2}d_{1}^{2}+P^{2}d_{1}^{2}-4\beta^{2}b_{0}^{2}+P^{2})}(-40\beta b_{0}c_{0}d_{1}^{5}d_{5}-192\beta b_{0}c_{0}d_{1}^{4}d_{2}d_{4}\\ -108\beta b_{0}c_{0}d_{1}^{4}d_{3}^{2}-288\beta b_{0}c_{0}d_{1}^{3}d_{2}^{2}d_{3}-32\beta b_{0}c_{0}d_{1}^{2}d_{2}^{4}-32\beta b_{0}c_{1}d_{1}^{5}d_{4}-144\beta b_{0}c_{1}d_{1}^{4}d_{2}d_{3}\\ -64\beta b_{0}c_{1}d_{1}^{3}d_{2}^{3}-24\beta b_{0}c_{2}d_{1}^{5}d_{3}-48\beta b_{0}c_{2}d_{1}^{4}d_{2}^{2}-16\beta b_{0}c_{3}d_{1}^{5}d_{2}-2\beta b_{0}c_{4}d_{1}^{6}-200\beta^{2}b_{0}^{2}d_{1}^{2}d_{2}d_{5}\\ -240\beta^{2}b_{0}^{2}d_{1}^{2}d_{3}d_{4}-120\beta^{2}b_{0}b_{1}d_{1}^{3}d_{5}-192\beta^{2}b_{0}b_{1}d_{1}^{2}d_{2}d_{4}-108\beta^{2}b_{0}b_{1}d_{1}^{2}d_{3}^{2}-72\beta^{2}b_{0}b_{2}d_{1}^{3}d_{4}\\ -108\beta^{2}b_{0}b_{2}d_{1}^{2}d_{2}d_{3}-36\beta^{2}b_{0}b_{3}d_{1}^{3}d_{3}-24\beta^{2}b_{0}b_{3}d_{1}^{2}d_{2}^{2}-12\beta^{2}b_{0}b_{4}d_{1}^{3}d_{2}-40\beta^{2}b_{1}^{2}d_{1}^{3}d_{4}\\ -60\beta^{2}b_{1}^{2}d_{1}^{2}d_{2}d_{3}-60\beta^{2}b_{1}b_{2}d_{1}^{3}d_{3}-40\beta^{2}b_{1}b_{2}d_{1}^{2}d_{2}^{2}-40\beta^{2}b_{1}b_{3}d_{1}^{3}d_{2}-10\beta^{2}b_{1}b_{4}d_{1}^{4}-20\beta^{2}b_{2}^{2}d_{1}^{3}d_{2}\\ -10\beta^{2}b_{2}b_{3}d_{1}^{4}-120\beta b_{0}^{2}d_{1}^{3}d_{5}-192\beta b_{0}^{2}d_{1}^{2}d_{2}d_{4}-108\beta b_{0}^{2}d_{1}^{2}d_{3}^{2}-152\beta b_{0}b_{1}d_{1}^{3}d_{4}-228\beta b_{0}b_{1}d_{1}^{2}d_{2}d_{3}\\ -96\beta b_{0}b_{2}d_{1}^{3}d_{3}-64\beta b_{0}b_{2}d_{1}^{2}d_{2}^{2}-52\beta b_{0}b_{3}d_{1}^{3}d_{2}-10\beta b_{0}b_{4}d_{1}^{4}-40\beta b_{0}c_{0}d_{1}^{3}d_{5}-64\beta b_{0}c_{0}d_{1}^{2}d_{2}d_{4}\\ -36\beta b_{0}c_{0}d_{1}^{2}d_{3}^{2}-32\beta b_{0}c_{1}d_{1}^{3}d_{4}-48\beta b_{0}c_{1}d_{1}^{2}d_{2}d_{3}-24\beta b_{0}c_{2}d_{1}^{3}d_{3}-16\beta b_{0}c_{2}d_{1}^{2}d_{2}^{2}-16\beta b_{0}c_{3}d_{1}^{3}d_{2}\\ -4\beta b_{0}c_{4}d_{1}^{4}-60\beta b_{1}^{2}d_{1}^{3}d_{3}-40\beta b_{1}^{2}d_{1}^{2}d_{2}^{2}-80\beta b_{1}b_{2}d_{1}^{3}d_{2}-20\beta b_{1}b_{3}d_{1}^{4}-10\beta b_{2}^{2}d_{1}^{4}+25P^{2}d_{1}^{2}d_{2}d_{5}\\ +30P^{2}d_{1}^{2}d_{3}d_{4}100\beta^{2}b_{0}^{2}d_{2}d_{5}-120\beta^{2}b_{0}^{2}d_{3}d_{4}-100\beta^{2}b_{0}b_{1}d_{1}d_{5}-160\beta^{2}b_{0}b_{1}d_{2}d_{4}-90\beta^{2}b_{0}b_{1}d_{3}^{2}\\ -80\beta^{2}b_{0}b_{2}d_{1}d_{4}-120\beta^{2}b_{0}b_{2}d_{2}d_{3}-60\beta^{2}b_{0}b_{3}d_{1}d_{3}-40\beta^{2}b_{0}b_{3}d_{2}^{2}-40\beta^{2}b_{0}b_{4}d_{1}d_{2}-40\beta^{2}b_{1}^{2}d_{1}d_{4}\\ -60\beta^{2}b_{1}^{2}d_{2}d_{3}-60\beta^{2}b_{1}b_{2}d_{1}d_{3}-40\beta^{2}b_{1}b_{2}d_{2}^{2}-40\beta^{2}b_{1}b_{3}d_{1}d_{2}-10\beta^{2}b_{1}b_{4}d_{1}^{2}-20\beta^{2}b_{2}^{2}d_{1}d_{2}\\ -10\beta^{2}b_{2}b_{3}d_{1}^{2}-40b_{0}^{2}d_{1}^{3}d_{4}-60b_{0}^{2}d_{1}^{2}d_{2}d_{3}-60b_{0}b_{1}d_{1}^{3}d_{3}-40b_{0}b_{1}d_{1}^{2}d_{2}^{2}-40b_{0}b_{2}d_{1}^{3}d_{2}-10b_{0}b_{3}d_{1}^{4}\\ -20b_{1}^{2}d_{1}^{3}d_{2}-10b_{1}b_{2}d_{1}^{4}-100\beta b_{0}^{2}d_{1}d_{5}-160\beta b_{0}^{2}d_{2}d_{4}-90\beta b_{0}^{2}d_{3}^{2}-160\beta b_{0}b_{1}d_{1}d_{4}-240\beta b_{0}b_{1}d_{2}d_{3}\\ -120\beta b_{0}b_{2}d_{1}d_{3}-80\beta b_{0}b_{2}d_{2}^{2}-80\beta b_{0}b_{3}d_{1}d_{2}-10\beta b_{0}b_{4}d_{1}^{2}-2\beta b_{0}c_{4}d_{1}^{2}-60\beta b_{1}^{2}d_{1}d_{3}-40\beta b_{1}^{2}d_{2}^{2}\\ -80\beta b_{1}b_{2}d_{1}d_{2}-20\beta b_{1}b_{3}d_{1}^{2}-10\beta b_{2}^{2}d_{1}^{2}+25P^{2}d_{2}d_{5}+30P^{2}d_{3}d_{4}-40b_{0}^{2}d_{1}d_{4}-60b_{0}^{2}d_{2}d_{3}\\ -60b_{0}b_{1}d_{1}d_{3}-40b_{0}b_{1}d_{2}^{2}-40b_{0}b_{2}d_{1}d_{2}-10b_{0}b_{3}d_{1}^{2}-20b_{1}^{2}d_{1}d_{2}-10b_{1}b_{2}d_{1}^{2}) \end{array}$$

$$\begin{array}{l} d_{7}=-\frac{1}{21d_{1}(-8\beta^{2}b_{0}^{2}d_{1}^{2}+P^{2}d_{1}^{2}-4\beta^{2}b_{0}^{2}+P^{2})}(-48\beta b_{0}c_{0}d_{1}^{5}d_{6}-240\beta b_{0}c_{0}d_{1}^{4}d_{2}d_{5}\\ -288\beta b_{0}c_{0}d_{1}^{4}d_{3}d_{4}-384\beta b_{0}c_{0}d_{1}^{3}d_{2}^{2}d_{4}-432\beta b_{0}c_{0}d_{1}^{3}d_{2}d_{3}^{2}-192\beta b_{0}c_{0}d_{1}^{2}d_{2}^{3}d_{3}-40\beta b_{0}c_{1}d_{1}^{5}d_{5}\\ -192\beta b_{0}c_{1}d_{1}^{4}d_{2}d_{4}-108\beta b_{0}c_{1}d_{1}^{4}d_{3}^{2}-288\beta b_{0}c_{1}d_{1}^{3}d_{2}^{2}d_{3}-32\beta b_{0}c_{1}d_{1}^{2}d_{2}^{4}-32\beta b_{0}c_{2}d_{1}^{5}d_{4}\\ -144\beta b_{0}c_{2}d_{1}^{4}d_{2}d_{3}-64\beta b_{0}c_{2}d_{1}^{3}d_{2}^{3}-24\beta b_{0}c_{3}d_{1}^{5}d_{3}-48\beta b_{0}c_{3}d_{1}^{4}d_{2}^{2}-16\beta b_{0}c_{4}d_{1}^{5}d_{2}-2\beta b_{0}c_{5}d_{1}^{6}\\ -288\beta^{2}b_{0}^{2}d_{1}^{2}d_{2}d_{6}-360\beta^{2}b_{0}^{2}d_{1}^{2}d_{3}d_{5}-192\beta^{2}b_{0}^{2}d_{1}^{2}d_{4}^{2}-180\beta^{2}b_{0}b_{1}d_{1}^{3}d_{6}-300\beta^{2}b_{0}b_{1}d_{1}^{2}d_{2}d_{5}\\ -360\beta^{2}b_{0}b_{1}d_{1}^{2}d_{3}d_{4}-120\beta^{2}b_{0}b_{2}d_{1}^{3}d_{5}-192\beta^{2}b_{0}b_{2}d_{1}^{2}d_{2}d_{4}-108\beta^{2}b_{0}b_{2}d_{1}^{2}d_{3}^{2}-72\beta^{2}b_{0}b_{3}d_{1}^{3}d_{4}\\ -108\beta^{2}b_{0}b_{3}d_{1}^{2}d_{2}d_{3}-36\beta^{2}b_{0}b_{4}d_{1}^{3}d_{3}-24\beta^{2}b_{0}b_{4}d_{1}^{2}d_{2}^{2}-12\beta^{2}b_{0}b_{5}d_{1}^{3}d_{2}-60\beta^{2}b_{1}^{2}d_{1}^{3}d_{5}\\ -96\beta^{2}b_{1}^{2}d_{1}^{2}d_{2}d_{4}-54\beta^{2}b_{1}^{2}d_{1}^{2}d_{3}^{2}-96\beta^{2}b_{1}b_{2}d_{1}^{3}d_{4}-144\beta^{2}b_{1}b_{2}d_{1}^{2}d_{2}d_{3}-72\beta^{2}b_{1}b_{3}d_{1}^{3}d_{3}\\ -48\beta^{2}b_{1}b_{3}d_{1}^{2}d_{2}^{2}-48\beta^{2}b_{1}b_{4}d_{1}^{3}d_{2}-12\beta^{2}b_{1}b_{5}d_{1}^{4}-36\beta^{2}b_{2}^{2}d_{1}^{3}d_{3}-24\beta^{2}b_{2}^{2}d_{1}^{2}d_{2}^{2}-48\beta^{2}b_{2}b_{3}d_{1}^{3}d_{2}\\ -12\beta^{2}b_{2}b_{4}d_{1}^{4}-6\beta^{2}b_{3}^{2}d_{1}^{4}-180\beta b_{0}^{2}d_{1}^{3}d_{6}-300\beta b_{0}^{2}d_{1}^{2}d_{2}d_{5}-360\beta b_{0}^{2}d_{1}^{2}d_{3}d_{4}-240\beta b_{0}b_{1}d_{1}^{3}d_{5}\\ -384\beta b_{0}b_{1}d_{1}^{2}d_{2}d_{4}-216\beta b_{0}b_{1}d_{1}^{2}d_{3}^{2}-168\beta b_{0}b_{2}d_{1}^{3}d_{4}-32\beta b_{0}c_{2}d_{1}^{3}d_{4}-108\beta^{2}b_{0}b_{2}d_{3}^{2}-96\beta^{2}b_{0}^{2}d_{4}^{2}\\ -252\beta b_{0}b_{2}d_{1}^{2}d_{2}d_{3}-108\beta b_{0}b_{3}d_{1}^{3}d_{3}-72\beta b_{0}b_{3}d_{1}^{2}d_{2}^{2}-60\beta b_{0}b_{4}d_{1}^{3}d_{2}-12\beta b_{0}b_{5}d_{1}^{4}-48\beta b_{0}c_{0}d_{1}^{3}d_{6}\\ -80\beta b_{0}c_{0}d_{1}^{2}d_{2}d_{5}-96\beta b_{0}c_{0}d_{1}^{2}d_{3}d_{4}-40\beta b_{0}c_{1}d_{1}^{3}d_{5}-64\beta b_{0}c_{1}d_{1}^{2}d_{2}d_{4}-36\beta b_{0}c_{1}d_{1}^{2}d_{3}^{2}\\ -48\beta b_{0}c_{2}d_{1}^{2}d_{2}d_{3}-24\beta b_{0}c_{3}d_{1}^{3}d_{3}-16\beta b_{0}c_{3}d_{1}^{2}d_{2}^{2}-16\beta b_{0}c_{4}d_{1}^{3}d_{2}-4\beta b_{0}c_{5}d_{1}^{4}-96\beta b_{1}^{2}d_{1}^{3}d_{4}\\ -144\beta b_{1}^{2}d_{1}^{2}d_{2}d_{3}-144\beta b_{1}b_{2}d_{1}^{3}d_{3}-96\beta b_{1}b_{2}d_{1}^{2}d_{2}^{2}-96\beta b_{1}b_{3}d_{1}^{3}d_{2}-24\beta b_{1}b_{4}d_{1}^{4}-48\beta b_{2}^{2}d_{1}^{3}d_{2}\\ -24\beta b_{2}b_{3}d_{1}^{4}+36P^{2}d_{1}^{2}d_{2}d_{6}+45P^{2}d_{1}^{2}d_{3}d_{5}+24P^{2}d_{1}^{2}d_{4}^{2}-144\beta^{2}b_{0}^{2}d_{2}d_{6}-180\beta^{2}b_{0}^{2}d_{3}d_{5}\\ -144\beta^{2}b_{0}b_{1}d_{1}d_{6}-240\beta^{2}b_{0}b_{1}d_{2}d_{5}-288\beta^{2}b_{0}b_{1}d_{3}d_{4}-120\beta^{2}b_{0}b_{2}d_{1}d_{5}-192\beta^{2}b_{0}b_{2}d_{2}d_{4}\\ -96\beta^{2}b_{0}b_{3}d_{1}d_{4}-144\beta^{2}b_{0}b_{3}d_{2}d_{3}-72\beta^{2}b_{0}b_{4}d_{1}d_{3}-48\beta^{2}b_{0}b_{4}d_{2}^{2}-48\beta^{2}b_{0}b_{5}d_{1}d_{2}-60\beta^{2}b_{1}^{2}d_{1}d_{5}\\ -96\beta^{2}b_{1}^{2}d_{2}d_{4}-54\beta^{2}b_{1}^{2}d_{3}^{2}-96\beta^{2}b_{1}b_{2}d_{1}d_{4}-144\beta^{2}b_{1}b_{2}d_{2}d_{3}-72\beta^{2}b_{1}b_{3}d_{1}d_{3}-48\beta^{2}b_{1}b_{3}d_{2}^{2}\\ -48\beta^{2}b_{1}b_{4}d_{1}d_{2}-12\beta^{2}b_{1}b_{5}d_{1}^{2}-36\beta^{2}b_{2}^{2}d_{1}d_{3}-24\beta^{2}b_{2}^{2}d_{2}^{2}-48\beta^{2}b_{2}b_{3}d_{1}d_{2}-12\beta^{2}b_{2}b_{4}d_{1}^{2}\\ -60b_{0}^{2}d_{1}^{3}d_{5}-96b_{0}^{2}d_{1}^{2}d_{2}d_{4}-54b_{0}^{2}d_{1}^{2}d_{3}^{2}-96b_{0}b_{1}d_{1}^{3}d_{4}-144b_{0}b_{1}d_{1}^{2}d_{2}d_{3}-72b_{0}b_{2}d_{1}^{3}d_{3}-48b_{0}b_{2}d_{1}^{2}d_{2}^{2}\\ -48b_{0}b_{3}d_{1}^{3}d_{2}-12b_{0}b_{4}d_{1}^{4}-36b_{1}^{2}d_{1}^{3}d_{3}-24b_{1}^{2}d_{1}^{2}d_{2}^{2}-48b_{1}b_{2}d_{1}^{3}d_{2}-12b_{1}b_{3}d_{1}^{4}-6b_{2}^{2}d_{1}^{4}-144\beta b_{0}^{2}d_{1}d_{6}\\ -240\beta b_{0}^{2}d_{2}d_{5}-288\beta b_{0}^{2}d_{3}d_{4}-240\beta b_{0}b_{1}d_{1}d_{5}-384\beta b_{0}b_{1}d_{2}d_{4}-216\beta b_{0}b_{1}d_{3}^{2}-192\beta b_{0}b_{2}d_{1}d_{4}\\ -288\beta b_{0}b_{2}d_{2}d_{3}-144\beta b_{0}b_{3}d_{1}d_{3}-96\beta b_{0}b_{3}d_{2}^{2}-96\beta b_{0}b_{4}d_{1}d_{2}-12\beta b_{0}b_{5}d_{1}^{2}-2\beta b_{0}c_{5}d_{1}^{2}\\ -144\beta b_{1}^{2}d_{2}d_{3}-144\beta b_{1}b_{2}d_{1}d_{3}-96\beta b_{1}b_{2}d_{2}^{2}-96\beta b_{1}b_{3}d_{1}d_{2}-24\beta b_{1}b_{4}d_{1}^{2}-48\beta b_{2}^{2}d_{1}d_{2}\\ +36P^{2}d_{2}d_{6}+45P^{2}d_{3}d_{5}+24P^{2}d_{4}^{2}-60b_{0}^{2}d_{1}d_{5}-96b_{0}^{2}d_{2}d_{4}-54b_{0}^{2}d_{3}^{2}-96b_{0}b_{1}d_{1}d_{4}-144b_{0}b_{1}d_{2}d_{3}\\ -72b_{0}b_{2}d_{1}d_{3}-48b_{0}b_{2}d_{2}^{2}-48b_{0}b_{3}d_{1}d_{2}-12b_{0}b_{4}d_{1}^{2}-36b_{1}^{2}d_{1}d_{3}-24b_{1}^{2}d_{2}^{2}-48b_{1}b_{2}d_{1}d_{2}-12b_{1}b_{3}d_{1}^{2}\\ -24\beta b_{2}b_{3}d_{1}^{2}-96\beta b_{1}^{2}d_{1}d_{4}-6\beta^{2}b_{3}^{2}d_{1}^{2}-6b_{2}^{2}d_{1}^{2}) \end{array}$$

$$\begin{array}{l} d_{8}=-\frac{1}{28d_{1}(-8\beta^{2}b_{0}^{2}d_{1}^{2}+P^{2}d_{1}^{2}-4\beta^{2}b_{0}^{2}+P^{2})}(-56\beta^{2}b_{2}b_{3}d_{2}^{2}-84\beta^{2}b_{2}^{2}d_{2}d_{3}-56\beta^{2}b_{2}^{2}d_{1}d_{4}\\ -14\beta^{2}b_{1}b_{6}d_{1}^{2}-56\beta^{2}b_{1}b_{4}d_{2}^{2}+70P^{2}d_{4}d_{5}+49P^{2}d_{2}d_{7}+63P^{2}d_{3}d_{6}-56\beta b_{2}^{2}d_{2}^{2}-14\beta b_{3}^{2}d_{1}^{2}\\ -140b_{0}^{2}d_{2}d_{5}-224\beta b_{0}^{2}d_{4}^{2}-56\beta^{2}b_{0}b_{6}d_{1}d_{2}-84\beta^{2}b_{0}b_{5}d_{1}d_{3}-168\beta^{2}b_{0}b_{4}d_{2}d_{3}-112\beta^{2}b_{0}b_{4}d_{1}d_{4}\\ -126\beta b_{1}^{2}d_{3}^{2}-336\beta b_{0}^{2}d_{2}d_{6}-420\beta b_{0}^{2}d_{3}d_{5}-196\beta b_{0}^{2}d_{1}d_{7}-14\beta^{2}b_{3}b_{4}d_{1}^{2}-28\beta^{2}b_{3}^{2}d_{1}d_{2}\\ -140\beta^{2}b_{0}b_{3}d_{1}d_{5}-224\beta^{2}b_{0}b_{3}d_{2}d_{4}-56b_{0}b_{4}d_{1}d_{2}-84b_{0}b_{3}d_{1}d_{3}-168\beta^{2}b_{0}b_{2}d_{1}d_{6}-280\beta^{2}b_{0}b_{2}d_{2}d_{5}\\ -336\beta^{2}b_{0}b_{2}d_{3}d_{4}-196\beta^{2}b_{0}b_{1}d_{1}d_{7}-336\beta^{2}b_{0}b_{1}d_{2}d_{6}-420\beta^{2}b_{0}b_{1}d_{3}d_{5}-336\beta b_{0}b_{3}d_{2}d_{3}\\ -256\beta b_{0}d_{1}^{2}c_{0}d_{2}^{3}d_{4}-192\beta b_{0}d_{1}^{4}c_{2}d_{2}d_{4}-144\beta b_{0}d_{1}^{4}c_{3}d_{2}d_{3}-288\beta b_{0}d_{1}^{3}c_{2}d_{2}^{2}d_{3}-288\beta b_{0}d_{1}^{4}c_{0}d_{2}d_{6}\\ -360\beta b_{0}d_{1}^{4}c_{0}d_{3}d_{5}-432\beta b_{0}d_{1}^{3}c_{1}d_{2}d_{3}^{2}-384\beta b_{0}d_{1}^{3}c_{1}d_{2}^{2}d_{4}-288\beta b_{0}d_{1}^{4}c_{1}d_{3}d_{4}-480\beta b_{0}d_{1}^{3}c_{0}d_{2}^{2}d_{5}\\ -240\beta b_{0}d_{1}^{4}c_{1}d_{2}d_{5}-432\beta^{2}b_{0}b_{1}d_{1}^{2}d_{2}d_{6}-540\beta^{2}b_{0}b_{1}d_{1}^{2}d_{3}d_{5}-300\beta^{2}b_{0}b_{2}d_{1}^{2}d_{2}d_{5}\\ -192\beta^{2}b_{0}b_{3}d_{1}^{2}d_{2}d_{4}-108\beta^{2}b_{0}b_{4}d_{1}^{2}d_{2}d_{3}-224\beta^{2}b_{1}b_{2}d_{1}^{2}d_{2}d_{4}-168\beta^{2}b_{1}b_{3}d_{1}^{2}d_{2}d_{3}\\ -696\beta b_{0}b_{1}d_{1}^{2}d_{3}d_{4}-416\beta b_{0}b_{2}d_{1}^{2}d_{2}d_{4}-276\beta b_{0}b_{3}d_{1}^{2}d_{2}d_{3}-336\beta b_{1}b_{2}d_{1}^{2}d_{2}d_{3}-64\beta b_{0}d_{1}^{2}c_{2}d_{2}d_{4}\\ -48\beta b_{0}d_{1}^{2}c_{3}d_{2}d_{3}-120\beta b_{0}d_{1}^{2}c_{0}d_{3}d_{5}-96\beta b_{0}d_{1}^{2}c_{1}d_{3}d_{4}-96\beta b_{0}d_{1}^{2}c_{0}d_{2}d_{6}-80\beta b_{0}d_{1}^{2}c_{1}d_{2}d_{5} \end{array}$$

$$\begin{array}{l} -192\beta b_{0}d_{1}^{2}c_{1}d_{2}^{3}d_{3}-432\beta b_{0}d_{1}^{2}c_{0}d_{2}^{2}d_{3}^{2}-14\beta b_{3}^{2}d_{1}^{4}-84b_{0}^{2}d_{1}^{3}d_{6}-14b_{0}b_{5}d_{1}^{4}-56b_{1}^{2}d_{1}^{3}d_{4}-14b_{1}b_{4}d_{1}^{4}\\ -28b_{2}^{2}d_{1}^{3}d_{2}-14b_{2}b_{3}d_{1}^{4}-1152\beta b_{0}d_{1}^{3}c_{0}d_{2}d_{3}d_{4}-672\beta b_{0}b_{1}d_{3}d_{4}-168b_{0}b_{2}d_{2}d_{3}-112b_{0}b_{2}d_{1}d_{4}\\ -224b_{0}b_{1}d_{2}d_{4}-140b_{0}b_{1}d_{1}d_{5}-32\beta b_{0}d_{1}^{2}c_{2}d_{2}^{4}-64\beta b_{0}d_{1}^{2}c_{0}d_{4}^{2}-24\beta b_{0}d_{1}^{3}c_{4}d_{3}-16\beta b_{0}d_{1}^{3}c_{5}d_{2}\\ -32\beta b_{0}d_{1}^{3}c_{3}d_{4}-40\beta b_{0}d_{1}^{3}c_{2}d_{5}-56\beta b_{0}d_{1}^{3}c_{0}d_{7}-48\beta b_{0}d_{1}^{3}c_{1}d_{6}-40\beta b_{0}d_{1}^{5}c_{2}d_{5}-108\beta b_{0}d_{1}^{4}c_{2}d_{3}^{2}\\ -64\beta b_{0}d_{1}^{3}c_{3}d_{2}^{3}-32\beta b_{0}d_{1}^{5}c_{3}d_{4}-24\beta b_{0}d_{1}^{5}c_{4}d_{3}-48\beta b_{0}d_{1}^{4}c_{4}d_{2}^{2}-16\beta b_{0}d_{1}^{5}c_{5}d_{2}-56\beta b_{0}d_{1}^{5}c_{0}d_{7}\\ -48\beta b_{0}d_{1}^{5}c_{1}d_{6}-192\beta b_{0}d_{1}^{4}c_{0}d_{4}^{2}-216\beta b_{0}d_{1}^{3}c_{0}d_{3}^{3}-224\beta b_{1}^{2}d_{1}^{2}d_{2}d_{4}-224\beta b_{1}b_{2}d_{1}^{3}d_{4}\\ -112\beta b_{1}b_{3}d_{1}^{2}d_{2}^{2}-112\beta b_{1}b_{4}d_{1}^{3}d_{2}-112\beta b_{2}b_{3}d_{1}^{3}d_{2}-224b_{0}b_{1}d_{1}^{2}d_{2}d_{4}-168b_{0}b_{2}d_{1}^{2}d_{2}d_{3}\\ -16\beta b_{0}d_{1}^{2}c_{4}d_{2}^{2}-392\beta^{2}b_{0}^{2}d_{1}^{2}d_{2}d_{7}-504\beta^{2}b_{0}^{2}d_{1}^{2}d_{3}d_{6}-560\beta^{2}b_{0}^{2}d_{1}^{2}d_{4}d_{5}-252\beta^{2}b_{0}b_{1}d_{1}^{3}d_{7}\\ -288\beta^{2}b_{0}b_{1}d_{1}^{2}d_{4}^{2}-180\beta^{2}b_{0}b_{2}d_{1}^{3}d_{6}-120\beta^{2}b_{0}b_{3}d_{1}^{3}d_{5}-108\beta^{2}b_{0}b_{3}d_{1}^{2}d_{3}^{2}-72\beta^{2}b_{0}b_{4}d_{1}^{3}d_{4}\\ -36\beta^{2}b_{0}b_{5}d_{1}^{3}d_{3}-24\beta^{2}b_{0}b_{5}d_{1}^{2}d_{2}^{2}-12\beta^{2}b_{0}b_{6}d_{1}^{3}d_{2}-140\beta^{2}b_{1}^{2}d_{1}^{2}d_{2}d_{5}-168\beta^{2}b_{1}^{2}d_{1}^{2}d_{3}d_{4}\\ -140\beta^{2}b_{1}b_{2}d_{1}^{3}d_{5}-126\beta^{2}b_{1}b_{2}d_{1}^{2}d_{3}^{2}-112\beta^{2}b_{1}b_{3}d_{1}^{3}d_{4}-84\beta^{2}b_{1}b_{4}d_{1}^{3}d_{3}-56\beta^{2}b_{1}b_{4}d_{1}^{2}d_{2}^{2}\\ -84\beta^{2}b_{2}^{2}d_{1}^{2}d_{2}d_{3}-84\beta^{2}b_{2}b_{3}d_{1}^{3}d_{3}-56\beta^{2}b_{2}b_{3}d_{1}^{2}d_{2}^{2}-56\beta^{2}b_{2}b_{4}d_{1}^{3}d_{2}-432\beta b_{0}^{2}d_{1}^{2}d_{2}d_{6}\\ -348\beta b_{0}b_{1}d_{1}^{3}d_{6}-260\beta b_{0}b_{2}d_{1}^{3}d_{5}-234\beta b_{0}b_{2}d_{1}^{2}d_{3}^{2}-184\beta b_{0}b_{3}d_{1}^{3}d_{4}-120\beta b_{0}b_{4}d_{1}^{3}d_{3}\\ -68\beta b_{0}b_{5}d_{1}^{3}d_{2}-56\beta^{2}b_{2}b_{4}d_{1}d_{2}-84\beta^{2}b_{2}b_{3}d_{1}d_{3}-56b_{1}b_{3}d_{1}d_{2}-84b_{1}b_{2}d_{1}d_{3}-280\beta b_{0}b_{2}d_{1}d_{5}\\ -448\beta b_{0}b_{2}d_{2}d_{4}-56\beta^{2}b_{1}b_{5}d_{1}d_{2}-84\beta^{2}b_{1}b_{4}d_{1}d_{3}-168\beta^{2}b_{1}b_{3}d_{2}d_{3}-224\beta b_{1}^{2}d_{2}d_{4}-14\beta b_{0}b_{6}d_{1}^{2}\\ -140\beta b_{1}^{2}d_{1}d_{5}-112\beta b_{0}b_{4}d_{2}^{2}-252\beta b_{0}b_{2}d_{3}^{2}-112\beta^{2}b_{1}b_{3}d_{1}d_{4}-14b_{2}b_{3}d_{1}^{2}-14b_{1}b_{4}d_{1}^{2}-56b_{1}^{2}d_{1}d_{4}\\ -84\beta b_{2}^{2}d_{1}d_{3}-28\beta b_{1}b_{5}d_{1}^{2}-112\beta b_{1}b_{3}d_{2}^{2}-224\beta^{2}b_{1}b_{2}d_{2}d_{4}-140\beta^{2}b_{1}b_{2}d_{1}d_{5}-56b_{1}b_{2}d_{2}^{2}\\ -28b_{2}^{2}d_{1}d_{2}-84b_{1}^{2}d_{2}d_{3}-14b_{0}b_{5}d_{1}^{2}-126b_{0}b_{1}d_{3}^{2}-56b_{0}b_{3}d_{2}^{2}-84b_{0}^{2}d_{1}d_{6}-224\beta b_{1}b_{2}d_{1}d_{4}\\ -126b_{0}b_{1}d_{1}^{2}d_{3}^{2}-112b_{0}b_{2}d_{1}^{3}d_{4}-84b_{0}b_{3}d_{1}^{3}d_{3}-56b_{0}b_{3}d_{1}^{2}d_{2}^{2}-56b_{0}b_{4}d_{1}^{3}d_{2}84b_{1}^{2}d_{1}^{2}d_{2}d_{3}-84b_{1}b_{2}d_{1}^{3}d_{3}\\ -56b_{1}b_{2}d_{1}^{2}d_{2}^{2}-56b_{1}b_{3}d_{1}^{3}d_{2}-4\beta b_{0}d_{1}^{4}c_{6}-2\beta b_{0}d_{1}^{6}c_{6}-2\beta b_{0}d_{1}^{2}c_{6}-84\beta^{2}b_{1}^{2}d_{1}^{3}d_{6}-14\beta^{2}b_{1}b_{6}d_{1}^{4}\\ -56\beta^{2}b_{2}^{2}d_{1}^{3}d_{4}-14\beta^{2}b_{2}b_{5}d_{1}^{4}-28\beta^{2}b_{3}^{2}d_{1}^{3}d_{2}-14\beta^{2}b_{3}b_{4}d_{1}^{4}-252\beta b_{0}^{2}d_{1}^{3}d_{7}-288\beta b_{0}^{2}d_{1}^{2}d_{4}^{2}\\ -140\beta b_{1}^{2}d_{1}^{3}d_{5}-126\beta b_{1}^{2}d_{1}^{2}d_{3}^{2}-28\beta b_{1}b_{5}d_{1}^{4}-84\beta b_{2}^{2}d_{1}^{3}d_{3}-56\beta b_{2}^{2}d_{1}^{2}d_{2}^{2}-28\beta b_{2}b_{4}d_{1}^{4}\\ +63P^{2}d_{1}^{2}d_{3}d_{6}+70P^{2}d_{1}^{2}d_{4}d_{5}-140b_{0}^{2}d_{1}^{2}d_{2}d_{5}-168b_{0}^{2}d_{1}^{2}d_{3}d_{4}-168\beta b_{0}b_{4}d_{1}d_{3}-112\beta b_{0}b_{5}d_{1}d_{2}\\ -196\beta^{2}b_{0}^{2}d_{2}d_{7}-252\beta^{2}b_{0}^{2}d_{3}d_{6}-280\beta^{2}b_{0}^{2}d_{4}d_{5}-224\beta^{2}b_{0}b_{1}d_{4}^{2}-126\beta^{2}b_{0}b_{3}d_{3}^{2}-56\beta^{2}b_{0}b_{5}d_{2}^{2}\\ -84\beta^{2}b_{1}^{2}d_{1}d_{6}-140\beta^{2}b_{1}^{2}d_{2}d_{5}-168\beta^{2}b_{1}^{2}d_{3}d_{4}-126\beta^{2}b_{1}b_{2}d_{3}^{2}-560\beta b_{0}b_{1}d_{2}d_{5}-336\beta b_{0}b_{1}d_{1}d_{6}\\ -168\beta b_{1}b_{3}d_{1}^{3}d_{3}-580\beta b_{0}b_{1}d_{1}^{2}d_{2}d_{5}-360\beta^{2}b_{0}b_{2}d_{1}^{2}d_{3}d_{4}-14\beta^{2}b_{2}b_{5}d_{1}^{2}-168b_{0}^{2}d_{3}d_{4}-28\beta b_{2}b_{4}d_{1}^{2}\\ -80\beta b_{0}b_{4}d_{1}^{2}d_{2}^{2}-540\beta b_{0}^{2}d_{1}^{2}d_{3}d_{5}-56\beta^{2}b_{1}b_{5}d_{1}^{3}d_{2}-36\beta b_{0}d_{1}^{2}c_{2}d_{3}^{2}+49P^{2}d_{1}^{2}d_{2}d_{7}-14\beta b_{0}b_{6}d_{1}^{4}\\ -112\beta b_{1}b_{4}d_{1}d_{2}-336\beta b_{1}b_{2}d_{2}d_{3}-168\beta b_{1}b_{3}d_{1}d_{3}-224\beta b_{0}b_{3}d_{1}d_{4}-112\beta b_{2}b_{3}d_{1}d_{2}\\ -140b_{0}b_{1}d_{1}^{3}d_{5}) \end{array}$$

$$\begin{array}{l} d_{9}=-\frac{1}{18d_{1}(-8\beta^{2}b_{0}^{2}d_{1}^{2}+P^{2}d_{1}^{2}-4\beta^{2}b_{0}^{2}+P^{2})}(-32b_{0}b_{5}d_{1}d_{2}-256\beta b_{0}b_{1}d_{4}^{2}-64\beta b_{0}b_{6}d_{1}d_{2}\\ -480\beta b_{0}b_{1}d_{3}d_{5}-96b_{1}b_{2}d_{2}d_{3}-64b_{1}b_{2}d_{1}d_{4}-128\beta^{2}b_{0}^{2}d_{2}d_{8}-168\beta^{2}b_{0}^{2}d_{3}d_{7}-192\beta^{2}b_{0}^{2}d_{4}d_{6}\\ -32\beta^{2}b_{2}b_{4}d_{2}^{2}-96b_{1}b_{2}d_{1}^{2}d_{2}d_{3}-8\beta b_{0}d_{1}^{3}c_{6}d_{2}-16\beta b_{0}d_{1}^{3}c_{4}d_{4}-20\beta b_{0}d_{1}^{3}c_{3}d_{5}-24\beta b_{0}d_{1}^{3}c_{2}d_{6}\\ -32\beta b_{0}d_{1}^{3}c_{0}d_{8}-12\beta b_{0}d_{1}^{3}c_{5}d_{3}-28\beta b_{0}d_{1}^{3}c_{1}d_{7}-8\beta b_{0}d_{1}^{5}c_{6}d_{2}-24\beta b_{0}d_{1}^{4}c_{5}d_{2}^{2}-32\beta b_{0}d_{1}^{3}c_{4}d_{2}^{3}\\ -12\beta b_{0}d_{1}^{5}c_{5}d_{3}-54\beta b_{0}d_{1}^{4}c_{3}d_{3}^{2}-16\beta b_{0}d_{1}^{5}c_{4}d_{4}-20\beta b_{0}d_{1}^{5}c_{3}d_{5}-108\beta b_{0}d_{1}^{3}c_{1}d_{3}^{3}-96\beta b_{0}d_{1}^{4}c_{1}d_{4}^{2}\\ -24\beta b_{0}d_{1}^{5}c_{2}d_{6}-32\beta b_{0}d_{1}^{5}c_{0}d_{8}-28\beta b_{0}d_{1}^{5}c_{1}d_{7}-18\beta b_{0}d_{1}^{2}c_{3}d_{3}^{2}-8\beta b_{0}d_{1}^{2}c_{5}d_{2}^{2}-16\beta b_{0}d_{1}^{2}c_{3}d_{2}^{4}\\ -32\beta b_{0}d_{1}^{2}c_{1}d_{4}^{2}-4b_{3}^{2}d_{1}^{4}-16\beta b_{1}b_{6}d_{1}^{2}-8b_{2}b_{4}d_{1}^{2}-24b_{2}^{2}d_{1}d_{3}-96\beta b_{0}b_{5}d_{1}d_{3}-160b_{0}b_{1}d_{2}d_{5}\\ -160\beta b_{1}b_{2}d_{1}d_{5}-384\beta b_{0}b_{2}d_{3}d_{4}-160\beta b_{0}b_{3}d_{1}d_{5}-256\beta b_{0}b_{3}d_{2}d_{4}-96\beta b_{2}b_{3}d_{1}d_{3}-48b_{0}b_{4}d_{1}d_{3}\\ -128\beta b_{0}b_{4}d_{1}d_{4}-320\beta b_{0}^{2}d_{4}d_{5}-224\beta b_{0}^{2}d_{2}d_{7}-288\beta b_{0}^{2}d_{3}d_{6}-128\beta b_{0}^{2}d_{1}d_{8}-18\beta^{2}b_{0}b_{6}d_{1}^{3}d_{3}\\ -12\beta^{2}b_{0}b_{6}d_{1}^{2}d_{2}^{2}-6\beta^{2}b_{0}b_{7}d_{1}^{3}d_{2}-96\beta^{2}b_{1}^{2}d_{1}^{2}d_{2}d_{6}-120\beta^{2}b_{1}^{2}d_{1}^{2}d_{3}d_{5}-96\beta^{2}b_{1}b_{2}d_{1}^{3}d_{6}\\ -72\beta^{2}b_{1}b_{3}d_{1}^{2}d_{3}^{2}-64\beta^{2}b_{1}b_{4}d_{1}^{3}d_{4}-48\beta^{2}b_{1}b_{5}d_{1}^{3}d_{3}-32\beta^{2}b_{1}b_{5}d_{1}^{2}d_{2}^{2}-32\beta^{2}b_{1}b_{6}d_{1}^{3}d_{2}\\ -64\beta^{2}b_{2}b_{3}d_{1}^{3}d_{4}-48\beta^{2}b_{2}b_{4}d_{1}^{3}d_{3}-32\beta^{2}b_{2}b_{4}d_{1}^{2}d_{2}^{2}-32\beta^{2}b_{2}b_{5}d_{1}^{3}d_{2}-32\beta^{2}b_{3}b_{4}d_{1}^{3}d_{2}\\ -378\beta b_{0}^{2}d_{1}^{2}d_{3}d_{6}-420\beta b_{0}^{2}d_{1}^{2}d_{4}d_{5}-238\beta b_{0}b_{1}d_{1}^{3}d_{7}-272\beta b_{0}b_{1}d_{1}^{2}d_{4}^{2}-186\beta b_{0}b_{2}d_{1}^{3}d_{6} \end{array}$$

$$\begin{array}{l} -126\beta b_{0}b_{3}d_{1}^{2}d_{3}^{2}-100\beta b_{0}b_{4}d_{1}^{3}d_{4}-66\beta b_{0}b_{5}d_{1}^{3}d_{3}-44\beta b_{0}b_{5}d_{1}^{2}d_{2}^{2}-38\beta b_{0}b_{6}d_{1}^{3}d_{2}-36\beta^{2}b_{2}^{2}d_{3}^{2}\\ -192\beta b_{1}^{2}d_{1}^{2}d_{3}d_{4}-160\beta b_{1}b_{2}d_{1}^{3}d_{5}-144\beta b_{1}b_{2}d_{1}^{2}d_{3}^{2}-128\beta b_{1}b_{3}d_{1}^{3}d_{4}-96\beta b_{1}b_{4}d_{1}^{3}d_{3}\\ -64\beta b_{1}b_{5}d_{1}^{3}d_{2}-96\beta b_{2}^{2}d_{1}^{2}d_{2}d_{3}-96\beta b_{2}b_{3}d_{1}^{3}d_{3}-64\beta b_{2}b_{3}d_{1}^{2}d_{2}^{2}-64\beta b_{2}b_{4}d_{1}^{3}d_{2}-160b_{0}b_{1}d_{1}^{2}d_{2}d_{5}\\ -192b_{0}b_{1}d_{1}^{2}d_{3}d_{4}-128b_{0}b_{2}d_{1}^{2}d_{2}d_{4}-96b_{0}b_{3}d_{1}^{2}d_{2}d_{3}-96b_{0}b_{3}d_{2}d_{3}-64b_{0}b_{3}d_{1}d_{4}-120b_{0}^{2}d_{3}d_{5}\\ +48P^{2}d_{4}d_{6}-96b_{0}^{2}d_{2}d_{6}+32P^{2}d_{2}d_{8}-56b_{0}^{2}d_{1}d_{7}+42P^{2}d_{3}d_{7}-96\beta b_{2}^{2}d_{2}d_{3}-72\beta^{2}b_{1}b_{3}d_{3}^{2}\\ -720\beta b_{0}d_{1}^{3}c_{0}d_{2}d_{3}d_{5}-576\beta b_{0}d_{1}^{2}c_{0}d_{2}^{2}d_{3}d_{4}-576\beta b_{0}d_{1}^{3}c_{1}d_{2}d_{3}d_{4}-4\beta^{2}b_{4}^{2}d_{1}^{2}-16\beta^{2}b_{3}^{2}d_{2}^{2}\\ -100\beta^{2}b_{0}^{2}d_{5}^{2}-64\beta^{2}b_{1}^{2}d_{4}^{2}-64\beta b_{2}b_{4}d_{1}d_{2}-8\beta^{2}b_{2}b_{6}d_{1}^{2}-24\beta^{2}b_{3}^{2}d_{1}d_{3}-8\beta^{2}b_{3}b_{5}d_{1}^{2}\\ -64\beta b_{2}^{2}d_{1}d_{4}-32b_{1}b_{4}d_{1}d_{2}-48b_{1}b_{3}d_{1}d_{3}-4b_{3}^{2}d_{1}^{2}-16b_{2}^{2}d_{2}^{2}-36b_{1}^{2}d_{3}^{2}-384\beta b_{0}b_{1}d_{2}d_{6}\\ -32b_{2}b_{3}d_{1}d_{2}-64b_{0}^{2}d_{4}^{2}+25P^{2}d_{5}^{2}-256\beta^{2}b_{0}^{2}d_{1}^{2}d_{2}d_{8}-336\beta^{2}b_{0}^{2}d_{1}^{2}d_{3}d_{7}-384\beta^{2}b_{0}^{2}d_{1}^{2}d_{4}d_{6}\\ -168\beta^{2}b_{0}b_{1}d_{1}^{3}d_{8}-126\beta^{2}b_{0}b_{2}d_{1}^{3}d_{7}-144\beta^{2}b_{0}b_{2}d_{1}^{2}d_{4}^{2}-90\beta^{2}b_{0}b_{3}d_{1}^{3}d_{6}-60\beta^{2}b_{0}b_{4}d_{1}^{3}d_{5}\\ -36\beta^{2}b_{0}b_{5}d_{1}^{3}d_{4}-32\beta^{2}b_{1}b_{5}d_{2}^{2}-8\beta^{2}b_{1}b_{7}d_{1}^{2}-40\beta^{2}b_{2}^{2}d_{1}d_{5}-64\beta^{2}b_{2}^{2}d_{2}d_{4}-240\beta b_{0}d_{1}^{3}c_{1}d_{2}^{2}d_{5}\\ -180\beta b_{0}d_{1}^{4}c_{1}d_{3}d_{5}-144\beta b_{0}d_{1}^{4}c_{1}d_{2}d_{6}-432\beta b_{0}d_{1}^{3}c_{0}d_{3}^{2}d_{4}-384\beta b_{0}d_{1}^{3}c_{0}d_{2}d_{4}^{2}-288\beta b_{0}d_{1}^{3}c_{0}d_{2}^{2}d_{6}\\ -240\beta b_{0}d_{1}^{4}c_{0}d_{4}d_{5}-216\beta b_{0}d_{1}^{4}c_{0}d_{3}d_{6}-168\beta b_{0}d_{1}^{4}c_{0}d_{2}d_{7}-510\beta b_{0}b_{1}d_{1}^{2}d_{3}d_{5}-310\beta b_{0}b_{2}d_{1}^{2}d_{2}d_{5}\\ -372\beta b_{0}b_{2}d_{1}^{2}d_{3}d_{4}-224\beta b_{0}b_{3}d_{1}^{2}d_{2}d_{4}-150\beta b_{0}b_{4}d_{1}^{2}d_{2}d_{3}-256\beta b_{1}b_{2}d_{1}^{2}d_{2}d_{4}-192\beta b_{1}b_{3}d_{1}^{2}d_{2}d_{3}\\ -40\beta b_{0}d_{1}^{2}c_{2}d_{2}d_{5}-72\beta b_{0}d_{1}^{2}c_{0}d_{3}d_{6}-80\beta b_{0}d_{1}^{2}c_{0}d_{4}d_{5}-48\beta b_{0}d_{1}^{2}c_{2}d_{3}d_{4}-60\beta b_{0}d_{1}^{2}c_{1}d_{3}d_{5}\\ -56\beta b_{0}d_{1}^{2}c_{0}d_{2}d_{7}-48\beta b_{0}d_{1}^{2}c_{1}d_{2}d_{6}-24\beta b_{0}d_{1}^{2}c_{4}d_{2}d_{3}-32\beta b_{0}d_{1}^{2}c_{3}d_{2}d_{4}-96\beta b_{0}d_{1}^{2}c_{2}d_{2}^{3}d_{3}\\ -216\beta b_{0}d_{1}^{2}c_{1}d_{2}^{2}d_{3}^{2}-128\beta b_{0}d_{1}^{2}c_{1}d_{2}^{3}d_{4}-216\beta b_{0}d_{1}^{2}c_{0}d_{2}d_{3}^{3}-160\beta b_{0}d_{1}^{2}c_{0}d_{2}^{3}d_{5}-294\beta^{2}b_{0}b_{1}d_{1}^{2}d_{2}d_{7}\\ -378\beta^{2}b_{0}b_{1}d_{1}^{2}d_{3}d_{6}-420\beta^{2}b_{0}b_{1}d_{1}^{2}d_{4}d_{5}-216\beta^{2}b_{0}b_{2}d_{1}^{2}d_{2}d_{6}-270\beta^{2}b_{0}b_{2}d_{1}^{2}d_{3}d_{5}\\ -180\beta^{2}b_{0}b_{3}d_{1}^{2}d_{3}d_{4}-96\beta^{2}b_{0}b_{4}d_{1}^{2}d_{2}d_{4}-54\beta^{2}b_{0}b_{5}d_{1}^{2}d_{2}d_{3}-160\beta^{2}b_{1}b_{2}d_{1}^{2}d_{2}d_{5}-192\beta^{2}b_{1}b_{2}d_{1}^{2}d_{3}d_{4}\\ -216\beta b_{0}d_{1}^{3}c_{2}d_{2}d_{3}^{2}-96\beta b_{0}d_{1}^{4}c_{3}d_{2}d_{4}-144\beta b_{0}d_{1}^{3}c_{3}d_{2}^{2}d_{3}-72\beta b_{0}d_{1}^{4}c_{4}d_{2}d_{3}-192\beta b_{0}d_{1}^{3}c_{2}d_{2}^{2}d_{4}\\ -144\beta b_{0}d_{1}^{4}c_{2}d_{3}d_{4}-120\beta b_{0}d_{1}^{4}c_{2}d_{2}d_{5}-128\beta^{2}b_{1}b_{3}d_{1}^{2}d_{2}d_{4}-96\beta^{2}b_{1}b_{4}d_{1}^{2}d_{2}d_{3}-96\beta^{2}b_{2}b_{3}d_{1}^{2}d_{2}d_{3}\\ -408\beta b_{0}b_{1}d_{1}^{2}d_{2}d_{6}-144\beta b_{1}b_{2}d_{3}^{2}-64\beta b_{1}b_{5}d_{1}d_{2}-16\beta b_{3}b_{4}d_{1}^{2}-32\beta b_{3}^{2}d_{1}d_{2}-16\beta b_{2}b_{5}d_{1}^{2}\\ -192\beta b_{0}b_{2}d_{1}d_{6}-320\beta b_{0}b_{2}d_{2}d_{5}-32b_{1}b_{3}d_{2}^{2}-8b_{1}b_{5}d_{1}^{2}-256\beta b_{1}b_{2}d_{2}d_{4}-4\beta^{2}b_{4}^{2}d_{1}^{4}+25P^{2}d_{1}^{2}d_{5}^{2}\\ -56b_{0}^{2}d_{1}^{3}d_{7}-64b_{0}^{2}d_{1}^{2}d_{4}^{2}-8b_{0}b_{6}d_{1}^{4}-40b_{1}^{2}d_{1}^{3}d_{5}-36b_{1}^{2}d_{1}^{2}d_{3}^{2}-8b_{1}b_{5}d_{1}^{4}-24b_{2}^{2}d_{1}^{3}d_{3}-16b_{2}^{2}d_{1}^{2}d_{2}^{2}\\ -8b_{2}b_{4}d_{1}^{4}-64\beta b_{2}b_{3}d_{2}^{2}-128\beta^{2}b_{0}b_{2}d_{4}^{2}-72\beta^{2}b_{0}b_{4}d_{3}^{2}-32\beta^{2}b_{0}b_{6}d_{2}^{2}-56\beta^{2}b_{1}^{2}d_{1}d_{7}-96\beta^{2}b_{1}^{2}d_{2}d_{6}\\ -140\beta b_{0}b_{3}d_{1}^{3}d_{5}-294\beta b_{0}^{2}d_{1}^{2}d_{2}d_{7}-64\beta^{2}b_{2}^{2}d_{1}^{2}d_{2}d_{4}-80\beta^{2}b_{1}b_{3}d_{1}^{3}d_{5}-96b_{0}b_{1}d_{1}d_{6}-64\beta b_{1}b_{4}d_{1}^{2}d_{2}^{2}\\ -150\beta^{2}b_{0}b_{3}d_{1}^{2}d_{2}d_{5}-54\beta^{2}b_{0}b_{4}d_{1}^{2}d_{3}^{2}-224\beta b_{0}b_{1}d_{1}d_{7}-96\beta^{2}b_{1}b_{2}d_{1}d_{6}-160\beta b_{1}^{2}d_{1}^{2}d_{2}d_{5}\\ -120\beta^{2}b_{1}^{2}d_{3}d_{5}-8b_{0}b_{6}d_{1}^{2}-40b_{1}^{2}d_{1}d_{5}-64b_{1}^{2}d_{2}d_{4}-32b_{0}b_{4}d_{2}^{2}-80b_{0}b_{2}d_{1}d_{5}-192b_{0}b_{1}d_{3}d_{4}\\ -32b_{0}b_{4}d_{1}^{2}d_{2}^{2}-32b_{0}b_{5}d_{1}^{3}d_{2}-64b_{1}^{2}d_{1}^{2}d_{2}d_{4}-64b_{1}b_{2}d_{1}^{3}d_{4}-48b_{1}b_{3}d_{1}^{3}d_{3}-32b_{1}b_{3}d_{1}^{2}d_{2}^{2}-32b_{1}b_{4}d_{1}^{3}d_{2}\\ -32b_{2}b_{3}d_{1}^{3}d_{2}-\beta b_{0}d_{1}^{2}c_{7}-2\beta b_{0}d_{1}^{4}c_{7}-\beta b_{0}d_{1}^{6}c_{7}-200\beta^{2}b_{0}^{2}d_{1}^{2}d_{5}^{2}-56\beta^{2}b_{1}^{2}d_{1}^{3}d_{7}-64\beta^{2}b_{1}^{2}d_{1}^{2}d_{4}^{2}\\ -8\beta^{2}b_{1}b_{7}d_{1}^{4}-40\beta^{2}b_{2}^{2}d_{1}^{3}d_{5}-36\beta^{2}b_{2}^{2}d_{1}^{2}d_{3}^{2}-8\beta^{2}b_{2}b_{6}d_{1}^{4}-24\beta^{2}b_{3}^{2}d_{1}^{3}d_{3}-16\beta^{2}b_{3}^{2}d_{1}^{2}d_{2}^{2}\\ -168\beta b_{0}^{2}d_{1}^{3}d_{8}-8\beta b_{0}b_{7}d_{1}^{4}-96\beta b_{1}^{2}d_{1}^{3}d_{6}-16\beta b_{1}b_{6}d_{1}^{4}-64\beta b_{2}^{2}d_{1}^{3}d_{4}-16\beta b_{2}b_{5}d_{1}^{4}-32\beta b_{3}^{2}d_{1}^{3}d_{2}\\ -16\beta b_{3}b_{4}d_{1}^{4}+32P^{2}d_{1}^{2}d_{2}d_{8}+42P^{2}d_{1}^{2}d_{3}d_{7}+48P^{2}d_{1}^{2}d_{4}d_{6}-96b_{0}^{2}d_{1}^{2}d_{2}d_{6}-120b_{0}^{2}d_{1}^{2}d_{3}d_{5}\\ -80b_{0}b_{2}d_{1}^{3}d_{5}-72b_{0}b_{2}d_{1}^{2}d_{3}^{2}-64b_{0}b_{3}d_{1}^{3}d_{4}-48b_{0}b_{4}d_{1}^{3}d_{3}-64\beta b_{1}b_{4}d_{2}^{2}-96\beta b_{1}b_{4}d_{1}d_{3}\\ -192\beta b_{1}^{2}d_{3}d_{4}-96\beta b_{1}^{2}d_{1}d_{6}-8\beta b_{0}b_{7}d_{1}^{2}-128b_{0}b_{2}d_{2}d_{4}-64\beta b_{0}b_{5}d_{2}^{2}-32\beta^{2}b_{3}b_{4}d_{1}d_{2}\\ -128\beta b_{1}b_{3}d_{1}d_{4}-192\beta b_{1}b_{3}d_{2}d_{3}-192\beta b_{0}b_{4}d_{2}d_{3}-32\beta^{2}b_{2}b_{5}d_{1}d_{2}-48\beta^{2}b_{2}b_{4}d_{1}d_{3}\\ -64\beta^{2}b_{2}b_{3}d_{1}d_{4}-32\beta^{2}b_{1}b_{6}d_{1}d_{2}-48\beta^{2}b_{1}b_{5}d_{1}d_{3}-96\beta^{2}b_{1}b_{4}d_{2}d_{3}-64\beta^{2}b_{1}b_{4}d_{1}d_{4}\\ -80\beta^{2}b_{1}b_{3}d_{1}d_{5}-160\beta^{2}b_{1}b_{2}d_{2}d_{5}-192\beta^{2}b_{1}b_{2}d_{3}d_{4}-32\beta^{2}b_{0}b_{7}d_{1}d_{2}-48\beta^{2}b_{0}b_{6}d_{1}d_{3}\\ -96\beta^{2}b_{0}b_{5}d_{2}d_{3}-80\beta^{2}b_{0}b_{4}d_{1}d_{5}-128\beta^{2}b_{0}b_{4}d_{2}d_{4}-160\beta^{2}b_{0}b_{3}d_{2}d_{5}-192\beta^{2}b_{0}b_{3}d_{3}d_{4}\\ -240\beta^{2}b_{0}b_{2}d_{3}d_{5}-96\beta^{2}b_{0}b_{3}d_{1}d_{6}-128\beta^{2}b_{0}b_{1}d_{1}d_{8}-224\beta^{2}b_{0}b_{1}d_{2}d_{7}-288\beta^{2}b_{0}b_{1}d_{3}d_{6}\\ -320\beta^{2}b_{0}b_{1}d_{4}d_{5}-192\beta^{2}b_{0}b_{2}d_{2}d_{6}-128\beta^{2}b_{1}b_{3}d_{2}d_{4}-96\beta^{2}b_{2}b_{3}d_{2}d_{3}-64\beta^{2}b_{0}b_{5}d_{1}d_{4}\\ -112\beta^{2}b_{0}b_{2}d_{1}d_{7}-144\beta b_{0}b_{3}d_{3}^{2}-160\beta b_{1}^{2}d_{2}d_{5}-96b_{0}b_{1}d_{1}^{3}d_{6}-8\beta^{2}b_{3}b_{5}d_{1}^{4}-72b_{0}b_{2}d_{3}^{2}) \end{array}$$

$$\begin{array}{l} d_{10}=-\frac{1}{45d_{1}(-8\beta^{2}b_{0}^{2}d_{1}^{2}+P^{2}d_{1}^{2}-4\beta^{2}b_{0}^{2}+P^{2})}(-108b_{0}b_{5}d_{1}^{3}d_{3}-72b_{0}b_{5}d_{1}^{2}d_{2}^{2}-72b_{0}b_{6}d_{1}^{3}d_{2}\\ -180b_{1}^{2}d_{1}^{2}d_{2}d_{5}-216b_{1}^{2}d_{1}^{2}d_{3}d_{4}-180b_{1}b_{2}d_{1}^{3}d_{5}-162b_{1}b_{2}d_{1}^{2}d_{3}^{2}-144b_{1}b_{3}d_{1}^{3}d_{4}-108b_{1}b_{4}d_{1}^{3}d_{3}\\ -72b_{1}b_{4}d_{1}^{2}d_{2}^{2}-72b_{1}b_{5}d_{1}^{3}d_{2}-108b_{2}^{2}d_{1}^{2}d_{2}d_{3}-108b_{2}b_{3}d_{1}^{3}d_{3}-72b_{2}b_{3}d_{1}^{2}d_{2}^{2}-72b_{2}b_{4}d_{1}^{3}d_{2}\\ -18\beta^{2}b_{4}b_{5}d_{1}^{4}-432\beta b_{0}^{2}d_{1}^{3}d_{9}-600\beta b_{0}^{2}d_{1}^{2}d_{5}^{2}-18\beta b_{0}b_{8}d_{1}^{4}-252\beta b_{1}^{2}d_{1}^{3}d_{7}-288\beta b_{1}^{2}d_{1}^{2}d_{4}^{2}\\ -36\beta b_{1}b_{7}d_{1}^{4}-180\beta b_{2}^{2}d_{1}^{3}d_{5}-162\beta b_{2}^{2}d_{1}^{2}d_{3}^{2}-36\beta b_{2}b_{6}d_{1}^{4}-108\beta b_{3}^{2}d_{1}^{3}d_{3}-72\beta b_{3}^{2}d_{1}^{2}d_{2}^{2}\\ +81P^{2}d_{1}^{2}d_{2}d_{9}+108P^{2}d_{1}^{2}d_{3}d_{8}+126P^{2}d_{1}^{2}d_{4}d_{7}+135P^{2}d_{1}^{2}d_{5}d_{6}-252b_{0}^{2}d_{1}^{2}d_{2}d_{7}-324b_{0}^{2}d_{1}^{2}d_{3}d_{6}\\ -360b_{0}^{2}d_{1}^{2}d_{4}d_{5}-252b_{0}b_{1}d_{1}^{3}d_{7}-288b_{0}b_{1}d_{1}^{2}d_{4}^{2}-216b_{0}b_{2}d_{1}^{3}d_{6}-180b_{0}b_{3}d_{1}^{3}d_{5}-162b_{0}b_{3}d_{1}^{2}d_{3}^{2}\\ -144b_{0}b_{4}d_{1}^{3}d_{4}-144\beta^{2}b_{1}^{2}d_{1}^{3}d_{8}-18\beta^{2}b_{1}b_{8}d_{1}^{4}-108\beta^{2}b_{2}^{2}d_{1}^{3}d_{6}-18\beta^{2}b_{2}b_{7}d_{1}^{4}-72\beta^{2}b_{3}^{2}d_{1}^{3}d_{4}\\ -18\beta^{2}b_{3}b_{6}d_{1}^{4}-2\beta b_{0}d_{1}^{2}c_{8}-4\beta b_{0}d_{1}^{4}c_{8}-2\beta b_{0}d_{1}^{6}c_{8}-48\beta b_{0}d_{1}^{5}c_{3}d_{6}-64\beta b_{0}d_{1}^{3}c_{5}d_{2}^{3}\\ -192\beta b_{0}d_{1}^{4}c_{2}d_{4}^{2}-56\beta b_{0}d_{1}^{5}c_{2}d_{7}-64\beta b_{0}d_{1}^{5}c_{1}d_{8}-300\beta b_{0}d_{1}^{4}c_{0}d_{5}^{2}-72\beta b_{0}d_{1}^{5}c_{0}d_{9}-16\beta b_{0}d_{1}^{3}c_{7}d_{2}\\ -24\beta b_{0}d_{1}^{3}c_{6}d_{3}-72\beta b_{0}d_{1}^{3}c_{0}d_{9}-32\beta b_{0}d_{1}^{3}c_{5}d_{4}-64\beta b_{0}d_{1}^{3}c_{1}d_{8}-56\beta b_{0}d_{1}^{3}c_{2}d_{7}-64\beta b_{0}d_{1}^{2}c_{2}d_{4}^{2}\\ -36\beta b_{0}d_{1}^{2}c_{4}d_{3}^{2}-16\beta b_{0}d_{1}^{2}c_{6}d_{2}^{2}-32\beta b_{0}d_{1}^{2}c_{4}d_{2}^{4}-100\beta b_{0}d_{1}^{2}c_{0}d_{5}^{2}-162\beta b_{0}d_{1}^{2}c_{0}d_{3}^{4}\\ -216b_{1}b_{3}d_{1}^{2}d_{2}d_{3}-16\beta b_{0}d_{1}^{5}c_{7}d_{2}-48\beta b_{0}d_{1}^{4}c_{6}d_{2}^{2}-24\beta b_{0}d_{1}^{5}c_{6}d_{3}-32\beta b_{0}d_{1}^{5}c_{5}d_{4}-108\beta b_{0}d_{1}^{4}c_{4}d_{3}^{2}\\ -48\beta b_{0}d_{1}^{3}c_{3}d_{6}-40\beta b_{0}d_{1}^{3}c_{4}d_{5}-40\beta b_{0}d_{1}^{5}c_{4}d_{5}-432b_{0}b_{1}d_{1}^{2}d_{2}d_{6}-540b_{0}b_{1}d_{1}^{2}d_{3}d_{5}\\ -432b_{0}b_{2}d_{1}^{2}d_{3}d_{4}-288b_{0}b_{3}d_{1}^{2}d_{2}d_{4}-216b_{0}b_{4}d_{1}^{2}d_{2}d_{3}-180\beta^{2}b_{1}b_{4}d_{1}^{3}d_{5}-162\beta^{2}b_{1}b_{4}d_{1}^{2}d_{3}^{2}\\ -144\beta^{2}b_{1}b_{5}d_{1}^{3}d_{4}-108\beta^{2}b_{1}b_{6}d_{1}^{3}d_{3}-72\beta^{2}b_{1}b_{6}d_{1}^{2}d_{2}^{2}-72\beta^{2}b_{1}b_{7}d_{1}^{3}d_{2}-180\beta^{2}b_{2}^{2}d_{1}^{2}d_{2}d_{5}\\ -216\beta^{2}b_{2}^{2}d_{1}^{2}d_{3}d_{4}-180\beta^{2}b_{2}b_{3}d_{1}^{3}d_{5}-162\beta^{2}b_{2}b_{3}d_{1}^{2}d_{3}^{2}-144\beta^{2}b_{2}b_{4}d_{1}^{3}d_{4}-108\beta^{2}b_{2}b_{5}d_{1}^{3}d_{3}\\ -72\beta^{2}b_{2}b_{5}d_{1}^{2}d_{2}^{2}-72\beta^{2}b_{2}b_{6}d_{1}^{3}d_{2}-108\beta^{2}b_{3}^{2}d_{1}^{2}d_{2}d_{3}-108\beta^{2}b_{3}b_{4}d_{1}^{3}d_{3}-72\beta^{2}b_{3}b_{4}d_{1}^{2}d_{2}^{2}\\ -768\beta b_{0}^{2}d_{1}^{2}d_{2}d_{8}-1008\beta b_{0}^{2}d_{1}^{2}d_{3}d_{7}-1152\beta b_{0}^{2}d_{1}^{2}d_{4}d_{6}-624\beta b_{0}b_{1}d_{1}^{3}d_{8}-504\beta b_{0}b_{2}d_{1}^{3}d_{7}\\ -576\beta b_{0}b_{2}d_{1}^{2}d_{4}^{2}-396\beta b_{0}b_{3}d_{1}^{3}d_{6}-300\beta b_{0}b_{4}d_{1}^{3}d_{5}-270\beta b_{0}b_{4}d_{1}^{2}d_{3}^{2}-216\beta b_{0}b_{5}d_{1}^{3}d_{4}\\ -96\beta b_{0}b_{6}d_{1}^{2}d_{2}^{2}-84\beta b_{0}b_{7}d_{1}^{3}d_{2}-432\beta b_{1}^{2}d_{1}^{2}d_{2}d_{6}-540\beta b_{1}^{2}d_{1}^{2}d_{3}d_{5}-432\beta b_{1}b_{2}d_{1}^{3}d_{6}\\ -324\beta b_{1}b_{3}d_{1}^{2}d_{3}^{2}-288\beta b_{1}b_{4}d_{1}^{3}d_{4}-216\beta b_{1}b_{5}d_{1}^{3}d_{3}-144\beta b_{1}b_{5}d_{1}^{2}d_{2}^{2}-144\beta b_{1}b_{6}d_{1}^{3}d_{2}\\ -288\beta b_{2}b_{3}d_{1}^{3}d_{4}-216\beta b_{2}b_{4}d_{1}^{3}d_{3}-144\beta b_{2}b_{4}d_{1}^{2}d_{2}^{2}-144\beta b_{2}b_{5}d_{1}^{3}d_{2}-144\beta b_{3}b_{4}d_{1}^{3}d_{2}\\ -864\beta^{2}b_{0}^{2}d_{1}^{2}d_{3}d_{8}-1008\beta^{2}b_{0}^{2}d_{1}^{2}d_{4}d_{7}-1080\beta^{2}b_{0}^{2}d_{1}^{2}d_{5}d_{6}-432\beta^{2}b_{0}b_{1}d_{1}^{3}d_{9}-600\beta^{2}b_{0}b_{1}d_{1}^{2}d_{5}^{2}\\ -336\beta^{2}b_{0}b_{2}d_{1}^{3}d_{8}-252\beta^{2}b_{0}b_{3}d_{1}^{3}d_{7}-288\beta^{2}b_{0}b_{3}d_{1}^{2}d_{4}^{2}-180\beta^{2}b_{0}b_{4}d_{1}^{3}d_{6}-120\beta^{2}b_{0}b_{5}d_{1}^{3}d_{5}\\ -108\beta^{2}b_{0}b_{5}d_{1}^{2}d_{3}^{2}-72\beta^{2}b_{0}b_{6}d_{1}^{3}d_{4}-36\beta^{2}b_{0}b_{7}d_{1}^{3}d_{3}-24\beta^{2}b_{0}b_{7}d_{1}^{2}d_{2}^{2}-12\beta^{2}b_{0}b_{8}d_{1}^{3}d_{2}\\ -324\beta^{2}b_{1}^{2}d_{1}^{2}d_{3}d_{6}-360\beta^{2}b_{1}^{2}d_{1}^{2}d_{4}d_{5}-252\beta^{2}b_{1}b_{2}d_{1}^{3}d_{7}-288\beta^{2}b_{1}b_{2}d_{1}^{2}d_{4}^{2}-216\beta^{2}b_{1}b_{3}d_{1}^{3}d_{6}\\ -144b_{0}^{2}d_{1}^{3}d_{8}-18b_{0}b_{7}d_{1}^{4}-108b_{1}^{2}d_{1}^{3}d_{6}-18b_{1}b_{6}d_{1}^{4}-72b_{2}^{2}d_{1}^{3}d_{4}-18b_{2}b_{5}d_{1}^{4}-36b_{3}^{2}d_{1}^{3}d_{2}\\ -432\beta b_{0}b_{3}d_{1}d_{6}-720\beta b_{0}b_{3}d_{2}d_{5}-864\beta b_{0}b_{3}d_{3}d_{4}-360\beta b_{0}b_{4}d_{1}d_{5}-576\beta b_{0}b_{4}d_{2}d_{4}\\ -432\beta b_{0}b_{5}d_{2}d_{3}-216\beta b_{0}b_{6}d_{1}d_{3}-144\beta b_{0}b_{7}d_{1}d_{2}-432\beta b_{1}b_{2}d_{1}d_{6}-720\beta b_{1}b_{2}d_{2}d_{5}\\ -360\beta b_{1}b_{3}d_{1}d_{5}-576\beta b_{1}b_{3}d_{2}d_{4}-288\beta b_{1}b_{4}d_{1}d_{4}-432\beta b_{1}b_{4}d_{2}d_{3}-216\beta b_{1}b_{5}d_{1}d_{3}\\ -288\beta b_{2}b_{3}d_{1}d_{4}-432\beta b_{2}b_{3}d_{2}d_{3}-216\beta b_{2}b_{4}d_{1}d_{3}-144\beta b_{2}b_{5}d_{1}d_{2}-144\beta b_{3}b_{4}d_{1}d_{2}\\ -756\beta^{2}b_{0}b_{1}d_{3}d_{7}-864\beta^{2}b_{0}b_{1}d_{4}d_{6}-288\beta^{2}b_{0}b_{2}d_{1}d_{8}-504\beta^{2}b_{0}b_{2}d_{2}d_{7}-648\beta^{2}b_{0}b_{2}d_{3}d_{6}\\ -720\beta^{2}b_{0}b_{2}d_{4}d_{5}-252\beta^{2}b_{0}b_{3}d_{1}d_{7}-432\beta^{2}b_{0}b_{3}d_{2}d_{6}-540\beta^{2}b_{0}b_{3}d_{3}d_{5}-216\beta^{2}b_{0}b_{4}d_{1}d_{6}\\ -360\beta^{2}b_{0}b_{4}d_{2}d_{5}-432\beta^{2}b_{0}b_{4}d_{3}d_{4}-180\beta^{2}b_{0}b_{5}d_{1}d_{5}-288\beta^{2}b_{0}b_{5}d_{2}d_{4}-144\beta^{2}b_{0}b_{6}d_{1}d_{4}\\ -216\beta^{2}b_{0}b_{6}d_{2}d_{3}-108\beta^{2}b_{0}b_{7}d_{1}d_{3}-72\beta^{2}b_{0}b_{8}d_{1}d_{2}-252\beta^{2}b_{1}b_{2}d_{1}d_{7}-432\beta^{2}b_{1}b_{2}d_{2}d_{6}\\ -540\beta^{2}b_{1}b_{2}d_{3}d_{5}-216\beta^{2}b_{1}b_{3}d_{1}d_{6}-360\beta^{2}b_{1}b_{3}d_{2}d_{5}-432\beta^{2}b_{1}b_{3}d_{3}d_{4}-180\beta^{2}b_{1}b_{4}d_{1}d_{5}\\ -288\beta^{2}b_{1}b_{4}d_{2}d_{4}-144\beta^{2}b_{1}b_{5}d_{1}d_{4}-216\beta^{2}b_{1}b_{5}d_{2}d_{3}-108\beta^{2}b_{1}b_{6}d_{1}d_{3}-72\beta^{2}b_{1}b_{7}d_{1}d_{2}\\ -360b_{0}b_{2}d_{1}^{2}d_{2}d_{5}-288b_{1}b_{2}d_{1}^{2}d_{2}d_{4}-216\beta b_{0}d_{1}^{3}c_{2}d_{3}^{3}-36\beta b_{3}b_{5}d_{1}^{4}-36\beta^{2}b_{4}^{2}d_{1}^{3}d_{2}\\ -648\beta^{2}b_{0}^{2}d_{1}^{2}d_{2}d_{9}-288\beta b_{2}^{2}d_{1}^{2}d_{2}d_{4}-360\beta b_{1}b_{3}d_{1}^{3}d_{5}-144\beta b_{0}b_{6}d_{1}^{3}d_{3}-72\beta^{2}b_{3}b_{5}d_{1}^{3}d_{2}\\ -576\beta^{2}b_{0}b_{1}d_{2}d_{8}-144\beta b_{1}b_{6}d_{1}d_{2}-864\beta b_{1}b_{2}d_{3}d_{4}-288\beta b_{0}b_{5}d_{1}d_{4}-252\beta^{2}b_{1}^{2}d_{1}^{2}d_{2}d_{7}\\ -180\beta^{2}b_{2}b_{3}d_{1}d_{5}-18b_{3}b_{4}d_{1}^{4}-18\beta b_{4}^{2}d_{1}^{4}-216b_{1}^{2}d_{3}d_{4}-1440\beta b_{0}b_{1}d_{4}d_{5}-108\beta b_{3}^{2}d_{1}d_{3} \end{array}$$

$$\begin{array}{l} -288\beta^{2}b_{2}b_{3}d_{2}d_{4}-144\beta^{2}b_{2}b_{4}d_{1}d_{4}-216\beta^{2}b_{2}b_{4}d_{2}d_{3}-108\beta^{2}b_{2}b_{5}d_{1}d_{3}-72\beta^{2}b_{2}b_{6}d_{1}d_{2}\\ -108\beta^{2}b_{3}b_{4}d_{1}d_{3}-72\beta^{2}b_{3}b_{5}d_{1}d_{2}-576\beta b_{0}b_{1}d_{1}d_{8}-1008\beta b_{0}b_{1}d_{2}d_{7}-1296\beta b_{0}b_{1}d_{3}d_{6}\\ -504\beta b_{0}b_{2}d_{1}d_{7}-864\beta b_{0}b_{2}d_{2}d_{6}-1080\beta b_{0}b_{2}d_{3}d_{5}-324\beta^{2}b_{0}b_{1}d_{1}d_{9}-72\beta b_{3}^{2}d_{2}^{2}-18\beta b_{4}^{2}d_{1}^{2}\\ +81P^{2}d_{2}d_{9}+108P^{2}d_{3}d_{8}+126P^{2}d_{4}d_{7}+135P^{2}d_{5}d_{6}-144b_{0}^{2}d_{1}d_{8}-252b_{0}^{2}d_{2}d_{7}-324b_{0}^{2}d_{3}d_{6}\\ -360b_{0}^{2}d_{4}d_{5}-288b_{0}b_{1}d_{4}^{2}-162b_{0}b_{3}d_{3}^{2}-72b_{0}b_{5}d_{2}^{2}-18b_{0}b_{7}d_{1}^{2}-108b_{1}^{2}d_{1}d_{6}-180b_{1}^{2}d_{2}d_{5}\\ -162b_{1}b_{2}d_{3}^{2}-72b_{1}b_{4}d_{2}^{2}-18b_{1}b_{6}d_{1}^{2}-72b_{2}^{2}d_{1}d_{4}-108b_{2}^{2}d_{2}d_{3}-72b_{2}b_{3}d_{2}^{2}-18b_{2}b_{5}d_{1}^{2}-36b_{3}^{2}d_{1}d_{2}\\ -18b_{3}b_{4}d_{1}^{2}-450\beta b_{0}^{2}d_{5}^{2}-288\beta b_{1}^{2}d_{4}^{2}-162\beta b_{2}^{2}d_{3}^{2}-18\beta^{2}b_{3}b_{6}d_{1}^{2}-36\beta^{2}b_{4}^{2}d_{1}d_{2}-18\beta^{2}b_{4}b_{5}d_{1}^{2}\\ -324\beta b_{0}^{2}d_{1}d_{9}-576\beta b_{0}^{2}d_{2}d_{8}-756\beta b_{0}^{2}d_{3}d_{7}-864\beta b_{0}^{2}d_{4}d_{6}-576\beta b_{0}b_{2}d_{4}^{2}-324\beta b_{0}b_{4}d_{3}^{2}\\ -144\beta b_{0}b_{6}d_{2}^{2}-18\beta b_{0}b_{8}d_{1}^{2}-252\beta b_{1}^{2}d_{1}d_{7}-432\beta b_{1}^{2}d_{2}d_{6}-540\beta b_{1}^{2}d_{3}d_{5}-324\beta b_{1}b_{3}d_{3}^{2}\\ -144\beta b_{1}b_{5}d_{2}^{2}-36\beta b_{1}b_{7}d_{1}^{2}-180\beta b_{2}^{2}d_{1}d_{5}-288\beta b_{2}^{2}d_{2}d_{4}-144\beta b_{2}b_{4}d_{2}^{2}-36\beta b_{2}b_{6}d_{1}^{2}\\ -36\beta b_{3}b_{5}d_{1}^{2}-252b_{0}b_{1}d_{1}d_{7}-432b_{0}b_{1}d_{2}d_{6}-540b_{0}b_{1}d_{3}d_{5}-216b_{0}b_{2}d_{1}d_{6}-360b_{0}b_{2}d_{2}d_{5}\\ -180b_{0}b_{3}d_{1}d_{5}-288b_{0}b_{3}d_{2}d_{4}-144b_{0}b_{4}d_{1}d_{4}-216b_{0}b_{4}d_{2}d_{3}-108b_{0}b_{5}d_{1}d_{3}-72b_{0}b_{6}d_{1}d_{2}\\ -288b_{1}b_{2}d_{2}d_{4}-144b_{1}b_{3}d_{1}d_{4}-216b_{1}b_{3}d_{2}d_{3}-108b_{1}b_{4}d_{1}d_{3}-72b_{1}b_{5}d_{1}d_{2}-108b_{2}b_{3}d_{1}d_{3}\\ -324\beta^{2}b_{0}^{2}d_{2}d_{9}-432\beta^{2}b_{0}^{2}d_{3}d_{8}-504\beta^{2}b_{0}^{2}d_{4}d_{7}-540\beta^{2}b_{0}^{2}d_{5}d_{6}-450\beta^{2}b_{0}b_{1}d_{5}^{2}-288\beta^{2}b_{0}b_{3}d_{4}^{2}\\ -162\beta^{2}b_{0}b_{5}d_{3}^{2}-72\beta^{2}b_{0}b_{7}d_{2}^{2}-144\beta^{2}b_{1}^{2}d_{1}d_{8}-252\beta^{2}b_{1}^{2}d_{2}d_{7}-324\beta^{2}b_{1}^{2}d_{3}d_{6}-360\beta^{2}b_{1}^{2}d_{4}d_{5}\\ -288\beta^{2}b_{1}b_{2}d_{4}^{2}-162\beta^{2}b_{1}b_{4}d_{3}^{2}-72\beta^{2}b_{1}b_{6}d_{2}^{2}-18\beta^{2}b_{1}b_{8}d_{1}^{2}-108\beta^{2}b_{2}^{2}d_{1}d_{6}-180\beta^{2}b_{2}^{2}d_{2}d_{5}\\ -216\beta^{2}b_{2}^{2}d_{3}d_{4}-162\beta^{2}b_{2}b_{3}d_{3}^{2}-72\beta^{2}b_{2}b_{5}d_{2}^{2}-18\beta^{2}b_{2}b_{7}d_{1}^{2}-72\beta^{2}b_{3}^{2}d_{1}d_{4}-108\beta^{2}b_{3}^{2}d_{2}d_{3}\\ -72\beta^{2}b_{3}b_{4}d_{2}^{2}-360\beta^{2}b_{0}b_{4}d_{1}^{2}d_{3}d_{4}-192\beta^{2}b_{0}b_{5}d_{1}^{2}d_{2}d_{4}-288\beta^{2}b_{1}b_{4}d_{1}^{2}d_{2}d_{4}-216\beta^{2}b_{1}b_{5}d_{1}^{2}d_{2}d_{3}\\ -288\beta^{2}b_{2}b_{3}d_{1}^{2}d_{2}d_{4}-216\beta^{2}b_{2}b_{4}d_{1}^{2}d_{2}d_{3}-1092\beta b_{0}b_{1}d_{1}^{2}d_{2}d_{7}-1404\beta b_{0}b_{1}d_{1}^{2}d_{3}d_{6}-432b_{0}b_{2}d_{3}d_{4}\\ -864\beta b_{0}b_{2}d_{1}^{2}d_{2}d_{6}-1080\beta b_{0}b_{2}d_{1}^{2}d_{3}d_{5}-660\beta b_{0}b_{3}d_{1}^{2}d_{2}d_{5}-792\beta b_{0}b_{3}d_{1}^{2}d_{3}d_{4}-480\beta b_{0}b_{4}d_{1}^{2}d_{2}d_{4}\\ -324\beta b_{0}b_{5}d_{1}^{2}d_{2}d_{3}-720\beta b_{1}b_{2}d_{1}^{2}d_{2}d_{5}-864\beta b_{1}b_{2}d_{1}^{2}d_{3}d_{4}-576\beta b_{1}b_{3}d_{1}^{2}d_{2}d_{4}-432\beta b_{1}b_{4}d_{1}^{2}d_{2}d_{3}\\ -432\beta b_{2}b_{3}d_{1}^{2}d_{2}d_{3}-80\beta b_{0}d_{1}^{2}c_{3}d_{2}d_{5}-120\beta b_{0}d_{1}^{2}c_{2}d_{3}d_{5}-96\beta b_{0}d_{1}^{2}c_{3}d_{3}d_{4}-192\beta b_{0}d_{1}^{2}c_{3}d_{2}^{3}d_{3}\\ -432\beta b_{0}d_{1}^{2}c_{2}d_{2}^{2}d_{3}^{2}-256\beta b_{0}d_{1}^{2}c_{2}d_{2}^{3}d_{4}-160\beta b_{0}d_{1}^{2}c_{1}d_{4}d_{5}-320\beta b_{0}d_{1}^{2}c_{1}d_{2}^{3}d_{5}-432\beta b_{0}d_{1}^{2}c_{1}d_{2}d_{3}^{3}\\ -768\beta b_{0}d_{1}^{2}c_{0}d_{2}^{2}d_{4}^{2}-384\beta b_{0}d_{1}^{2}c_{0}d_{2}^{3}d_{6}-768\beta^{2}b_{0}b_{1}d_{1}^{2}d_{2}d_{8}-1008\beta^{2}b_{0}b_{1}d_{1}^{2}d_{3}d_{7}-72b_{2}b_{4}d_{1}d_{2}\\ -588\beta^{2}b_{0}b_{2}d_{1}^{2}d_{2}d_{7}-756\beta^{2}b_{0}b_{2}d_{1}^{2}d_{3}d_{6}-840\beta^{2}b_{0}b_{2}d_{1}^{2}d_{4}d_{5}-432\beta^{2}b_{0}b_{3}d_{1}^{2}d_{2}d_{6}-180b_{1}b_{2}d_{1}d_{5}\\ -300\beta^{2}b_{0}b_{4}d_{1}^{2}d_{2}d_{5}-336\beta b_{0}d_{1}^{4}c_{1}d_{2}d_{7}-1152\beta b_{0}d_{1}^{3}c_{0}d_{3}d_{4}^{2}-1080\beta b_{0}d_{1}^{3}c_{0}d_{3}^{2}d_{5}-48\beta b_{0}d_{1}^{2}c_{5}d_{2}d_{3}\\ -64\beta b_{0}d_{1}^{2}c_{4}d_{2}d_{4}-128\beta b_{0}d_{1}^{2}c_{0}d_{2}d_{8}-192\beta b_{0}d_{1}^{2}c_{0}d_{4}d_{6}-112\beta b_{0}d_{1}^{2}c_{1}d_{2}d_{7}-96\beta b_{0}d_{1}^{2}c_{2}d_{2}d_{6}\\ -144\beta b_{0}d_{1}^{2}c_{1}d_{3}d_{6}-168\beta b_{0}d_{1}^{2}c_{0}d_{3}d_{7}-144\beta b_{0}d_{1}^{4}c_{5}d_{2}d_{3}-288\beta b_{0}d_{1}^{3}c_{4}d_{2}^{2}d_{3}-192\beta b_{0}d_{1}^{4}c_{4}d_{2}d_{4}\\ -432\beta b_{0}d_{1}^{3}c_{3}d_{2}d_{3}^{2}-384\beta b_{0}d_{1}^{3}c_{3}d_{2}^{2}d_{4}-288\beta b_{0}d_{1}^{4}c_{3}d_{3}d_{4}-672\beta b_{0}d_{1}^{3}c_{0}d_{2}^{2}d_{7}-576\beta b_{0}d_{1}^{4}c_{0}d_{4}d_{6}\\ -504\beta b_{0}d_{1}^{4}c_{0}d_{3}d_{7}-384\beta b_{0}d_{1}^{4}c_{0}d_{2}d_{8}-240\beta b_{0}d_{1}^{4}c_{3}d_{2}d_{5}-480\beta b_{0}d_{1}^{3}c_{2}d_{2}^{2}d_{5}-360\beta b_{0}d_{1}^{4}c_{2}d_{3}d_{5}\\ -288\beta b_{0}d_{1}^{4}c_{2}d_{2}d_{6}-864\beta b_{0}d_{1}^{3}c_{1}d_{3}^{2}d_{4}-768\beta b_{0}d_{1}^{3}c_{1}d_{2}d_{4}^{2}-576\beta b_{0}d_{1}^{3}c_{1}d_{2}^{2}d_{6}-480\beta b_{0}d_{1}^{4}c_{1}d_{4}d_{5}\\ -432\beta b_{0}d_{1}^{4}c_{1}d_{3}d_{6}-108\beta^{2}b_{0}b_{6}d_{1}^{2}d_{2}d_{3}-432\beta^{2}b_{1}b_{2}d_{1}^{2}d_{2}d_{6}-540\beta^{2}b_{1}b_{2}d_{1}^{2}d_{3}d_{5}\\ -432\beta^{2}b_{1}b_{3}d_{1}^{2}d_{3}d_{4}-1440\beta b_{0}d_{1}^{3}c_{1}d_{2}d_{3}d_{5}-1920\beta b_{0}d_{1}^{3}c_{0}d_{2}d_{4}d_{5}-1728\beta b_{0}d_{1}^{3}c_{0}d_{2}d_{3}d_{6}\\ -360\beta^{2}b_{1}b_{3}d_{1}^{2}d_{2}d_{5}-540\beta^{2}b_{0}b_{3}d_{1}^{2}d_{3}d_{5}-1152\beta^{2}b_{0}b_{1}d_{1}^{2}d_{4}d_{6}-1560\beta b_{0}b_{1}d_{1}^{2}d_{4}d_{5}\\ -1152\beta b_{0}d_{1}^{3}c_{2}d_{2}d_{3}d_{4}-1152\beta b_{0}d_{1}^{2}c_{1}d_{2}^{2}d_{3}d_{4}-1440\beta b_{0}d_{1}^{2}c_{0}d_{2}^{2}d_{3}d_{5}-1728\beta b_{0}d_{1}^{2}c_{0}d_{2}d_{3}^{2}d_{4}) \end{array}$$

$$\begin{array}{l} c_{1}=-\frac{1}{(-4\beta^{2}b_{0}^{2}+P^{2})\beta }(4\beta^{2}vb_{0}^{2}c_{0}-4\beta^{2}b_{0}^{2}c_{0}-P^{2}vc_{0}-4\beta^{2}b_{0}^{2}+P^{2}c_{0}+P^{2}+(16\beta^{4}v^{2}b_{0}^{4}c_{0}^{2}\\ -32\beta^{4}vb_{0}^{5}c_{0}-8P^{2}\beta^{2}v^{2}b_{0}^{2}c_{0}^{2}-32\beta^{4}vb_{0}^{4}c_{0}+16\beta^{4}b_{0}^{6}+16P^{2}\beta^{2}vb_{0}^{3}c_{0}+32\beta^{4}b_{0}^{5}+P^{4}v^{2}c_{0}^{2}\\ +16P^{2}\beta^{2}vb_{0}^{2}c_{0}-8P^{2}\beta^{2}b_{0}^{4}+16\beta^{4}b_{0}^{4}-2P^{4}vb_{0}c_{0}-16P^{2}\beta^{2}b_{0}^{3}-2P^{4}vc_{0}+P^{4}b_{0}^{2}-12P^{2}\beta^{2}b_{0}^{2}\\ +2P^{4}b_{0}+2P^{4}{)}^{\frac{1}{2}}) \end{array}$$

$$\begin{array}{l} c_{2}=\frac{1}{2\beta (\beta vb_{1}-\beta c_{1}+vb_{0}-c_{0}-1)}(2\beta^{2}v^{2}b_{1}b_{2}-2\beta^{2}vb_{2}c_{1}+2\beta v^{2}b_{0}b_{2}+2\beta v^{2}b_{1}^{2}-4\beta vb_{1}c_{1}\\ -2\beta vb_{2}c_{0}+2v^{2}b_{0}b_{1}-v^{2}c_{0}c_{1}-2\beta vb_{2}+2\beta c_{1}^{2}-vb_{0}c_{1}-vb_{1}c_{0}-2vb_{1}+vc_{1}-b_{0}b_{1}+2c_{0}c_{1}\\ +2d_{1}d_{2}-b_{1}+2c_{1}) \end{array}$$

$$\begin{array}{l} c_{3}=\frac{1}{6\beta (\beta vb_{1}-\beta c_{1}+vb_{0}-c_{0}-1)}(6\beta^{2}v^{2}b_{1}b_{3}+4\beta^{2}v^{2}b_{2}^{2}-8\beta^{2}vb_{2}c_{2}-6\beta^{2}vb_{3}c_{1}+6\beta v^{2}b_{0}b_{3}\\ +14\beta v^{2}b_{1}b_{2}+4\beta^{2}c_{2}^{2}-14\beta vb_{1}c_{2}-14\beta vb_{2}c_{1}-6\beta vb_{3}c_{0}+6v^{2}b_{0}b_{2}+4v^{2}b_{1}^{2}-2v^{2}c_{0}c_{2}-v^{2}c_{1}^{2}\\ -6\beta vb_{3}+14\beta c_{1}c_{2}-4vb_{0}c_{2}-6vb_{1}c_{1}-4vb_{2}c_{0}-6vb_{2}+2vc_{2}-2b_{0}b_{2}-b_{1}^{2}+6c_{0}c_{2}+4c_{1}^{2}+6d_{1}d_{3}\\ +4d_{2}^{2}-2b_{2}+6c_{2}) \end{array}$$

$$\begin{array}{l} c_{4}=\frac{1}{4\beta (\beta vb_{1}-\beta c_{1}+vb_{0}-c_{0}-1)}(4\beta^{2}v^{2}b_{1}b_{4}+6\beta^{2}v^{2}b_{2}b_{3}-6\beta^{2}vb_{2}c_{3}-6\beta^{2}vb_{3}c_{2}-4\beta^{2}vb_{4}c_{1}\\ +4\beta v^{2}b_{0}b_{4}+10\beta v^{2}b_{1}b_{3}+6\beta v^{2}b_{2}^{2}+6\beta^{2}c_{2}c_{3}-10\beta vb_{1}c_{3}-12\beta vb_{2}c_{2}-10\beta vb_{3}c_{1}-4\beta vb_{4}c_{0}\\ +4v^{2}b_{0}b_{3}+6v^{2}b_{1}b_{2}-v^{2}c_{0}c_{3}-v^{2}c_{1}c_{2}-4\beta vb_{4}+10\beta c_{1}c_{3}+6\beta c_{2}^{2}-3vb_{0}c_{3}-5vb_{1}c_{2}-5vb_{2}c_{1}\\ -3vb_{3}c_{0}-4vb_{3}+vc_{3}-b_{0}b_{3}-b_{1}b_{2}+4c_{0}c_{3}+6c_{1}c_{2}+4d_{1}d_{4}+6d_{2}d_{3}-b_{3}+4c_{3}) \end{array}$$

$$\begin{array}{l} c_{5}=\frac{1}{10\beta (\beta vb_{1}-\beta c_{1}+vb_{0}-c_{0}-1)}(10\beta^{2}v^{2}b_{1}b_{5}+16\beta^{2}v^{2}b_{2}b_{4}+9\beta^{2}v^{2}b_{3}^{2}-16\beta^{2}vb_{2}c_{4}\\ -18\beta^{2}vb_{3}c_{3}-16\beta^{2}vb_{4}c_{2}-10\beta^{2}vb_{5}c_{1}+10\beta v^{2}b_{0}b_{5}+26\beta v^{2}b_{1}b_{4}+34\beta v^{2}b_{2}b_{3}+16\beta^{2}c_{2}c_{4}\\ +9\beta^{2}c_{3}^{2}-26\beta vb_{1}c_{4}-34\beta vb_{2}c_{3}-34\beta vb_{3}c_{2}-26\beta vb_{4}c_{1}-10\beta vb_{5}c_{0}+10v^{2}b_{0}b_{4}+16v^{2}b_{1}b_{3}\\ +9v^{2}b_{2}^{2}-2v^{2}c_{0}c_{4}-2v^{2}c_{1}c_{3}-v^{2}c_{2}^{2}-10\beta vb_{5}+26\beta c_{1}c_{4}+34\beta c_{2}c_{3}-8vb_{0}c_{4}-14vb_{1}c_{3}-16vb_{2}c_{2}\\ -14vb_{3}c_{1}-8vb_{4}c_{0}-10vb_{4}+2vc_{4}-2b_{0}b_{4}-2b_{1}b_{3}-b_{2}^{2}+10c_{0}c_{4}+16c_{1}c_{3}+9c_{2}^{2}+10d_{1}d_{5}\\ +16d_{2}d_{4}+9d_{3}^{2}-2b_{4}+10c_{4}) \end{array}$$

$$\begin{array}{l} c_{6}=\frac{1}{6\beta (\beta vb_{1}-\beta c_{1}+vb_{0}-c_{0}-1)}(6\beta^{2}v^{2}b_{1}b_{6}+10\beta^{2}v^{2}b_{2}b_{5}+12\beta^{2}v^{2}b_{3}b_{4}-10\beta^{2}vb_{2}c_{5}\\ -12\beta^{2}vb_{3}c_{4}-12\beta^{2}vb_{4}c_{3}-10\beta^{2}vb_{5}c_{2}-6\beta^{2}vb_{6}c_{1}+6\beta v^{2}b_{0}b_{6}+16\beta v^{2}b_{1}b_{5}+22\beta v^{2}b_{2}b_{4}\\ +12\beta v^{2}b_{3}^{2}+10\beta^{2}c_{2}c_{5}+12\beta^{2}c_{3}c_{4}-16\beta vb_{1}c_{5}-22\beta vb_{2}c_{4}-24\beta vb_{3}c_{3}-22\beta vb_{4}c_{2}-16\beta vb_{5}c_{1}\\ -6\beta vb_{6}c_{0}+6v^{2}b_{0}b_{5}+10v^{2}b_{1}b_{4}+12v^{2}b_{2}b_{3}-v^{2}c_{0}c_{5}-v^{2}c_{1}c_{4}-v^{2}c_{2}c_{3}-6\beta vb_{6}+16\beta c_{1}c_{5}\\ +22\beta c_{2}c_{4}+12\beta c_{3}^{2}-5vb_{0}c_{5}-9vb_{1}c_{4}-11vb_{2}c_{3}-11vb_{3}c_{2}-9vb_{4}c_{1}-5vb_{5}c_{0}-6vb_{5}+vc_{5}\\ -b_{0}b_{5}-b_{1}b_{4}-b_{2}b_{3}+6c_{0}c_{5}+10c_{1}c_{4}+12c_{2}c_{3}+6d_{1}d_{6}+10d_{2}d_{5}+12d_{3}d_{4}-b_{5}+6c_{5}) \end{array}$$

$$\begin{array}{l} c_{7}=\frac{1}{14\beta (\beta vb_{1}-\beta c_{1}+vb_{0}-c_{0}-1)}(14\beta^{2}v^{2}b_{1}b_{7}+24\beta^{2}v^{2}b_{2}b_{6}+30\beta^{2}v^{2}b_{3}b_{5}+16\beta^{2}v^{2}b_{4}^{2}\\ -24\beta^{2}vb_{2}c_{6}-30\beta^{2}vb_{3}c_{5}-32\beta^{2}vb_{4}c_{4}-30\beta^{2}vb_{5}c_{3}-24\beta^{2}vb_{6}c_{2}-14\beta^{2}vb_{7}c_{1}+14\beta v^{2}b_{0}b_{7}\\ +38\beta v^{2}b_{1}b_{6}+54\beta v^{2}b_{2}b_{5}+62\beta v^{2}b_{3}b_{4}+24\beta^{2}c_{2}c_{6}+30\beta^{2}c_{3}c_{5}+16\beta^{2}c_{4}^{2}-38\beta vb_{1}c_{6}\\ -54\beta vb_{2}c_{5}-62\beta vb_{3}c_{4}-62\beta vb_{4}c_{3}-54\beta vb_{5}c_{2}-38\beta vb_{6}c_{1}-14\beta vb_{7}c_{0}+14v^{2}b_{0}b_{6}+24v^{2}b_{1}b_{5}\\ +30v^{2}b_{2}b_{4}+16v^{2}b_{3}^{2}-2v^{2}c_{0}c_{6}-2v^{2}c_{1}c_{5}-2v^{2}c_{2}c_{4}-v^{2}c_{3}^{2}-14\beta vb_{7}+38\beta c_{1}c_{6}+54\beta c_{2}c_{5}\\ +62\beta c_{3}c_{4}-12vb_{0}c_{6}-22vb_{1}c_{5}-28vb_{2}c_{4}-30vb_{3}c_{3}-28vb_{4}c_{2}-22vb_{5}c_{1}-12vb_{6}c_{0}-14vb_{6}\\ +2vc_{6}-2b_{0}b_{6}-2b_{1}b_{5}-2b_{2}b_{4}-b_{3}^{2}+14c_{0}c_{6}+24c_{1}c_{5}+30c_{2}c_{4}+16c_{3}^{2}+14d_{1}d_{7}+24d_{2}d_{6}\\ +30d_{3}d_{5}+16d_{4}^{2}-2b_{6}+14c_{6}) \end{array}$$

$$\begin{array}{l} c_{8}=\frac{1}{8\beta (\beta vb_{1}-\beta c_{1}+vb_{0}-c_{0}-1)}(8\beta^{2}v^{2}b_{1}b_{8}+14\beta^{2}v^{2}b_{2}b_{7}+18\beta^{2}v^{2}b_{3}b_{6}+20\beta^{2}v^{2}b_{4}b_{5}\\ -14\beta^{2}vb_{2}c_{7}-18\beta^{2}vb_{3}c_{6}-20\beta^{2}vb_{4}c_{5}-20\beta^{2}vb_{5}c_{4}-18\beta^{2}vb_{6}c_{3}-14\beta^{2}vb_{7}c_{2}-8\beta^{2}vb_{8}c_{1}\\ +8\beta v^{2}b_{0}b_{8}+22\beta v^{2}b_{1}b_{7}+32\beta v^{2}b_{2}b_{6}+38\beta v^{2}b_{3}b_{5}+20\beta v^{2}b_{4}^{2}+14\beta^{2}c_{2}c_{7}+18\beta^{2}c_{3}c_{6}\\ +20\beta^{2}c_{4}c_{5}-22\beta vb_{1}c_{7}-32\beta vb_{2}c_{6}-38\beta vb_{3}c_{5}-40\beta vb_{4}c_{4}-38\beta vb_{5}c_{3}-32\beta vb_{6}c_{2}-22\beta vb_{7}c_{1}\\ -8\beta vb_{8}c_{0}+8v^{2}b_{0}b_{7}+14v^{2}b_{1}b_{6}+18v^{2}b_{2}b_{5}+20v^{2}b_{3}b_{4}-v^{2}c_{0}c_{7}-v^{2}c_{1}c_{6}-v^{2}c_{2}c_{5}-v^{2}c_{3}c_{4} \end{array}$$

$$\begin{array}{l} -8\beta vb_{8}+22\beta c_{1}c_{7}+32\beta c_{2}c_{6}+38\beta c_{3}c_{5}+20\beta c_{4}^{2}-7vb_{0}c_{7}-13vb_{1}c_{6}-17vb_{2}c_{5}-19vb_{3}c_{4}\\ -19vb_{4}c_{3}-17vb_{5}c_{2}-13vb_{6}c_{1}-7vb_{7}c_{0}-8vb_{7}+vc_{7}-b_{0}b_{7}-b_{1}b_{6}-b_{2}b_{5}-b_{3}b_{4}+8c_{0}c_{7}+14c_{1}c_{6}\\ +18c_{2}c_{5}+20c_{3}c_{4}+8d_{1}d_{8}+14d_{2}d_{7}+18d_{3}d_{6}+20d_{4}d_{5}-b_{7}+8c_{7}) \end{array}$$

$$\begin{array}{l} c_{9}=\frac{1}{18\beta (\beta vb_{1}-\beta c_{1}+vb_{0}-c_{0}-1)}(18\beta^{2}v^{2}b_{1}b_{9}+32\beta^{2}v^{2}b_{2}b_{8}+42\beta^{2}v^{2}b_{3}b_{7}+48\beta^{2}v^{2}b_{4}b_{6}\\ +25\beta^{2}v^{2}b_{5}^{2}-32\beta^{2}vb_{2}c_{8}-42\beta^{2}vb_{3}c_{7}-48\beta^{2}vb_{4}c_{6}-50\beta^{2}vb_{5}c_{5}-48\beta^{2}vb_{6}c_{4}-42\beta^{2}vb_{7}c_{3}\\ -32\beta^{2}vb_{8}c_{2}-18\beta^{2}vb_{9}c_{1}+18\beta v^{2}b_{0}b_{9}+50\beta v^{2}b_{1}b_{8}+74\beta v^{2}b_{2}b_{7}+90\beta v^{2}b_{3}b_{6}+98\beta v^{2}b_{4}b_{5}\\ +32\beta^{2}c_{2}c_{8}+42\beta^{2}c_{3}c_{7}+48\beta^{2}c_{4}c_{6}+25\beta^{2}c_{5}^{2}-50\beta vb_{1}c_{8}-74\beta vb_{2}c_{7}-90\beta vb_{3}c_{6}-98\beta vb_{4}c_{5}\\ -98\beta vb_{5}c_{4}-90\beta vb_{6}c_{3}-74\beta vb_{7}c_{2}-50\beta vb_{8}c_{1}-18\beta vb_{9}c_{0}+18v^{2}b_{0}b_{8}+32v^{2}b_{1}b_{7}+42v^{2}b_{2}b_{6}\\ +48v^{2}b_{3}b_{5}+25v^{2}b_{4}^{2}-2v^{2}c_{0}c_{8}-2v^{2}c_{1}c_{7}-2v^{2}c_{2}c_{6}-2v^{2}c_{3}c_{5}-v^{2}c_{4}^{2}-18\beta vb_{9}+50\beta c_{1}c_{8}\\ +74\beta c_{2}c_{7}+90\beta c_{3}c_{6}+98\beta c_{4}c_{5}-16vb_{0}c_{8}-30vb_{1}c_{7}-40vb_{2}c_{6}-46vb_{3}c_{5}-48vb_{4}c_{4}-46vb_{5}c_{3}\\ -40vb_{6}c_{2}-30vb_{7}c_{1}-16vb_{8}c_{0}-18vb_{8}+2vc_{8}-2b_{0}b_{8}-2b_{1}b_{7}-2b_{2}b_{6}-2b_{3}b_{5}-b_{4}^{2}+18c_{0}c_{8}\\ +32c_{1}c_{7}+42c_{2}c_{6}+48c_{3}c_{5}+25c_{4}^{2}+18d_{1}d_{9}+32d_{2}d_{8}+42d_{3}d_{7}+48d_{4}d_{6}+25d_{5}^{2}-2b_{8}+18c_{8}) \end{array}.$$

$$\begin{array}{l} c_{10}=\frac{1}{10\beta (\beta vb_{1}-\beta c_{1}+vb_{0}-c_{0}-1)}(10c_{0}c_{9}+10c_{9}+10d_{1}d_{10}+18d_{2}d_{9}+24d_{3}d_{8}+28d_{4}d_{7}\\ +30d_{5}d_{6}+30\beta v^{2}b_{5}^{2}+10v^{2}b_{0}b_{9}+18v^{2}b_{1}b_{8}+24v^{2}b_{2}b_{7}+28v^{2}b_{3}b_{6}+30v^{2}b_{4}b_{5}-v^{2}c_{0}c_{9}-v^{2}c_{1}c_{8}\\ -v^{2}c_{2}c_{7}-v^{2}c_{3}c_{6}-v^{2}c_{4}c_{5}-10\beta vb_{10}-9vb_{0}c_{9}-17vb_{1}c_{8}-23vb_{2}c_{7}-27vb_{3}c_{6}-29vb_{4}c_{5}\\ -29vb_{5}c_{4}-27vb_{6}c_{3}-23vb_{7}c_{2}-17vb_{8}c_{1}-9vb_{9}c_{0}+18\beta^{2}c_{2}c_{9}+24\beta^{2}c_{3}c_{8}+28\beta^{2}c_{4}c_{7}\\ +30\beta^{2}c_{5}c_{6}+28\beta c_{1}c_{9}+42\beta c_{2}c_{8}+52\beta c_{3}c_{7}+58\beta c_{4}c_{6}-10vb_{9}+vc_{9}+30\beta c_{5}^{2}-b_{9}-b_{0}b_{9}\\ -b_{1}b_{8}-b_{2}b_{7}-b_{3}b_{6}-b_{4}b_{5}+28c_{3}c_{6}+30c_{4}c_{5}+18c_{1}c_{8}+24c_{2}c_{7}+10\beta^{2}v^{2}b_{1}b_{10}+18\beta^{2}v^{2}b_{2}b_{9}\\ +24\beta^{2}v^{2}b_{3}b_{8}+28\beta^{2}v^{2}b_{4}b_{7}+30\beta^{2}v^{2}b_{5}b_{6}+10\beta v^{2}b_{0}b_{10}+28\beta v^{2}b_{1}b_{9}+42\beta v^{2}b_{2}b_{8}+52\beta v^{2}b_{3}b_{7}\\ +58\beta v^{2}b_{4}b_{6}-18\beta^{2}vb_{2}c_{9}-24\beta^{2}vb_{3}c_{8}-28\beta^{2}vb_{4}c_{7}-30\beta^{2}vb_{5}c_{6}-30\beta^{2}vb_{6}c_{5}-28\beta^{2}vb_{7}c_{4}\\ -24\beta^{2}vb_{8}c_{3}-18\beta^{2}vb_{9}c_{2}-10\beta^{2}vb_{10}c_{1}-28\beta vb_{1}c_{9}-42\beta vb_{2}c_{8}-52\beta vb_{3}c_{7}-58\beta vb_{4}c_{6}\\ -60\beta vb_{5}c_{5}-58\beta vb_{6}c_{4}-52\beta vb_{7}c_{3}-42\beta vb_{8}c_{2}-28\beta vb_{9}c_{1}-10\beta vb_{10}c_{0}) \end{array}$$

$$b_{1}=\frac{-b_{0}+c_{0}}{\beta }$$

$$\begin{array}{l} b_{2}=-\frac{1}{2\beta (-8\beta^{2}b_{0}^{2}d_{1}^{2}+P^{2}d_{1}^{2}-4\beta^{2}b_{0}^{2}+P^{2})}(32\beta^{2}b_{0}^{2}c_{0}d_{1}^{3}d_{2}+4\beta^{2}b_{0}^{2}c_{1}d_{1}^{4}-40\beta^{3}b_{0}^{2}b_{1}d_{1}d_{2}\\ -4\beta^{3}b_{0}b_{1}^{2}d_{1}^{2}-8P^{2}c_{0}d_{1}^{3}d_{2}-P^{2}c_{1}d_{1}^{4}-40\beta^{2}b_{0}^{3}d_{1}d_{2}-24\beta^{2}b_{0}^{2}b_{1}d_{1}^{2}+32\beta^{2}b_{0}^{2}c_{0}d_{1}d_{2}+8\beta^{2}b_{0}^{2}c_{1}d_{1}^{2}\\ +2P^{2}\beta b_{1}d_{1}d_{2}-4\beta b_{0}^{3}d_{1}^{2}+2P^{2}b_{0}d_{1}d_{2}+2P^{2}b_{1}d_{1}^{2}-8P^{2}c_{0}d_{1}d_{2}-2P^{2}c_{1}d_{1}^{2}-8\beta^{2}b_{0}^{2}b_{1}+4\beta^{2}b_{0}^{2}c_{1}\\ +2P^{2}b_{1}-P^{2}c_{1}) \end{array}$$

$$\begin{array}{l} b_{3}=-\frac{1}{3\beta (-8\beta^{2}b_{0}^{2}d_{1}^{2}+P^{2}d_{1}^{2}-4\beta^{2}b_{0}^{2}+P^{2})}(48\beta^{2}b_{0}^{2}c_{0}d_{1}^{3}d_{3}+96\beta^{2}b_{0}^{2}c_{0}d_{1}^{2}d_{2}^{2}+32\beta^{2}b_{0}^{2}c_{1}d_{1}^{3}d_{2}\\ +4\beta^{2}b_{0}^{2}c_{2}d_{1}^{4}-72\beta^{3}b_{0}^{2}b_{1}d_{1}d_{3}-48\beta^{3}b_{0}^{2}b_{1}d_{2}^{2}-72\beta^{3}b_{0}^{2}b_{2}d_{1}d_{2}-24\beta^{3}b_{0}b_{1}^{2}d_{1}d_{2}-12\beta^{3}b_{0}b_{1}b_{2}d_{1}^{2}\\ -12P^{2}c_{0}d_{1}^{3}d_{3}-24P^{2}c_{0}d_{1}^{2}d_{2}^{2}-8P^{2}c_{1}d_{1}^{3}d_{2}-P^{2}c_{2}d_{1}^{4}-72\beta^{2}b_{0}^{3}d_{1}d_{3}-48\beta^{2}b_{0}^{3}d_{2}^{2}-120\beta^{2}b_{0}^{2}b_{1}d_{1}d_{2}\\ -36\beta^{2}b_{0}^{2}b_{2}d_{1}^{2}+48\beta^{2}b_{0}^{2}c_{0}d_{1}d_{3}+32\beta^{2}b_{0}^{2}c_{0}d_{2}^{2}+32\beta^{2}b_{0}^{2}c_{1}d_{1}d_{2}+8\beta^{2}b_{0}^{2}c_{2}d_{1}^{2}-12\beta^{2}b_{0}b_{1}^{2}d_{1}^{2}\\ +6P^{2}\beta b_{2}d_{1}d_{2}-24\beta b_{0}^{3}d_{1}d_{2}-12\beta b_{0}^{2}b_{1}d_{1}^{2}+6P^{2}b_{1}d_{1}d_{2}+3P^{2}b_{2}d_{1}^{2}-12P^{2}c_{0}d_{1}d_{3}-8P^{2}c_{0}d_{2}^{2}\\ -8P^{2}c_{1}d_{1}d_{2}-2P^{2}c_{2}d_{1}^{2}-12\beta^{2}b_{0}^{2}b_{2}+4\beta^{2}b_{0}^{2}c_{2}+3P^{2}b_{2}-P^{2}c_{2}) \end{array}$$

$$\begin{array}{l} b_{4}=\frac{1}{4\beta (-8\beta^{2}b_{0}^{2}d_{1}^{2}+P^{2}d_{1}^{2}-4\beta^{2}b_{0}^{2}+P^{2})}(-64\beta^{2}b_{0}^{2}c_{0}d_{1}^{3}d_{4}-288\beta^{2}b_{0}^{2}c_{0}d_{1}^{2}d_{2}d_{3}-128\beta^{2}b_{0}^{2}c_{0}d_{1}d_{2}^{3}\\ -48\beta^{2}b_{0}^{2}c_{1}d_{1}^{3}d_{3}-96\beta^{2}b_{0}^{2}c_{1}d_{1}^{2}d_{2}^{2}-32\beta^{2}b_{0}^{2}c_{2}d_{1}^{3}d_{2}-4\beta^{2}b_{0}^{2}c_{3}d_{1}^{4}+112\beta^{3}b_{0}^{2}b_{1}d_{1}d_{4}+168\beta^{3}b_{0}^{2}b_{1}d_{2}d_{3}\\ +120\beta^{3}b_{0}^{2}b_{2}d_{1}d_{3}+80\beta^{3}b_{0}^{2}b_{2}d_{2}^{2}+104\beta^{3}b_{0}^{2}b_{3}d_{1}d_{2}+48\beta^{3}b_{0}b_{1}^{2}d_{1}d_{3}+32\beta^{3}b_{0}b_{1}^{2}d_{2}^{2}+64\beta^{3}b_{0}b_{1}b_{2}d_{1}d_{2} \end{array}$$

$$\begin{array}{l} +16\beta^{3}b_{0}b_{1}b_{3}d_{1}^{2}+8\beta^{3}b_{0}b_{2}^{2}d_{1}^{2}+16P^{2}c_{0}d_{1}^{3}d_{4}+72P^{2}c_{0}d_{1}^{2}d_{2}d_{3}+32P^{2}c_{0}d_{1}d_{2}^{3}+12P^{2}c_{1}d_{1}^{3}d_{3}\\ +24P^{2}c_{1}d_{1}^{2}d_{2}^{2}+8P^{2}c_{2}d_{1}^{3}d_{2}+P^{2}c_{3}d_{1}^{4}+112\beta^{2}b_{0}^{3}d_{1}d_{4}+168\beta^{2}b_{0}^{3}d_{2}d_{3}216\beta^{2}b_{0}^{2}b_{1}d_{1}d_{3}+144\beta^{2}b_{0}^{2}b_{1}d_{2}^{2}\\ +168\beta^{2}b_{0}^{2}b_{2}d_{1}d_{2}+48\beta^{2}b_{0}^{2}b_{3}d_{1}^{2}-64\beta^{2}b_{0}^{2}c_{0}d_{1}d_{4}-96\beta^{2}b_{0}^{2}c_{0}d_{2}d_{3}-48\beta^{2}b_{0}^{2}c_{1}d_{1}d_{3}-32\beta^{2}b_{0}^{2}c_{1}d_{2}^{2}\\ -32\beta^{2}b_{0}^{2}c_{2}d_{1}d_{2}-8\beta^{2}b_{0}^{2}c_{3}d_{1}^{2}+64\beta^{2}b_{0}b_{1}^{2}d_{1}d_{2}+32\beta^{2}b_{0}b_{1}b_{2}d_{1}^{2}+4P^{2}\beta b_{1}d_{1}d_{4}+6P^{2}\beta b_{1}d_{2}d_{3}\\ -6P^{2}\beta b_{2}d_{1}d_{3}-4P^{2}\beta b_{2}d_{2}^{2}-10P^{2}\beta b_{3}d_{1}d_{2}+48\beta b_{0}^{3}d_{1}d_{3}+32\beta b_{0}^{3}d_{2}^{2}+64\beta b_{0}^{2}b_{1}d_{1}d_{2}+16\beta b_{0}^{2}b_{2}d_{1}^{2}\\ +8\beta b_{0}b_{1}^{2}d_{1}^{2}+4P^{2}b_{0}d_{1}d_{4}+6P^{2}b_{0}d_{2}d_{3}-6P^{2}b_{1}d_{1}d_{3}-4P^{2}b_{1}d_{2}^{2}-10P^{2}b_{2}d_{1}d_{2}-4P^{2}b_{3}d_{1}^{2}\\ +16P^{2}c_{0}d_{1}d_{4}+24P^{2}c_{0}d_{2}d_{3}+12P^{2}c_{1}d_{1}d_{3}+8P^{2}c_{1}d_{2}^{2}+8P^{2}c_{2}d_{1}d_{2}+2P^{2}c_{3}d_{1}^{2}+16\beta^{2}b_{0}^{2}b_{3}-4\beta^{2}b_{0}^{2}c_{3}\\ -4P^{2}b_{3}+P^{2}c_{3}) \end{array}$$

$$\begin{array}{l} b_{5}=\frac{1}{5\beta (-8\beta^{2}b_{0}^{2}d_{1}^{2}+P^{2}d_{1}^{2}-4\beta^{2}b_{0}^{2}+P^{2})}(-80\beta^{2}b_{0}^{2}c_{0}d_{1}^{3}d_{5}-384\beta^{2}b_{0}^{2}c_{0}d_{1}^{2}d_{2}d_{4}-216\beta^{2}b_{0}^{2}c_{0}d_{1}^{2}d_{3}^{2}\\ -576\beta^{2}b_{0}^{2}c_{0}d_{1}d_{2}^{2}d_{3}-64\beta^{2}b_{0}^{2}c_{0}d_{2}^{4}-64\beta^{2}b_{0}^{2}c_{1}d_{1}^{3}d_{4}-288\beta^{2}b_{0}^{2}c_{1}d_{1}^{2}d_{2}d_{3}-128\beta^{2}b_{0}^{2}c_{1}d_{1}d_{2}^{3}\\ -48\beta^{2}b_{0}^{2}c_{2}d_{1}^{3}d_{3}-96\beta^{2}b_{0}^{2}c_{2}d_{1}^{2}d_{2}^{2}-32\beta^{2}b_{0}^{2}c_{3}d_{1}^{3}d_{2}-4\beta^{2}b_{0}^{2}c_{4}d_{1}^{4}+160\beta^{3}b_{0}^{2}b_{1}d_{1}d_{5}+256\beta^{3}b_{0}^{2}b_{1}d_{2}d_{4}\\ +144\beta^{3}b_{0}^{2}b_{1}d_{3}^{2}+176\beta^{3}b_{0}^{2}b_{2}d_{1}d_{4}+264\beta^{3}b_{0}^{2}b_{2}d_{2}d_{3}+168\beta^{3}b_{0}^{2}b_{3}d_{1}d_{3}+112\beta^{3}b_{0}^{2}b_{3}d_{2}^{2}\\ +136\beta^{3}b_{0}^{2}b_{4}d_{1}d_{2}+80\beta^{3}b_{0}b_{1}^{2}d_{1}d_{4}+120\beta^{3}b_{0}b_{1}^{2}d_{2}d_{3}+120\beta^{3}b_{0}b_{1}b_{2}d_{1}d_{3}+80\beta^{3}b_{0}b_{1}b_{2}d_{2}^{2}\\ -80\beta^{3}b_{0}b_{1}b_{3}d_{1}d_{2}+20\beta^{3}b_{0}b_{1}b_{4}d_{1}^{2}+40\beta^{3}b_{0}b_{2}^{2}d_{1}d_{2}+20\beta^{3}b_{0}b_{2}b_{3}d_{1}^{2}+20P^{2}c_{0}d_{1}^{3}d_{5}+96P^{2}c_{0}d_{1}^{2}d_{2}d_{4}\\ +54P^{2}c_{0}d_{1}^{2}d_{3}^{2}+144P^{2}c_{0}d_{1}d_{2}^{2}d_{3}+16P^{2}c_{0}d_{2}^{4}+16P^{2}c_{1}d_{1}^{3}d_{4}+72P^{2}c_{1}d_{1}^{2}d_{2}d_{3}+32P^{2}c_{1}d_{1}d_{2}^{3}\\ +12P^{2}c_{2}d_{1}^{3}d_{3}+24P^{2}c_{2}d_{1}^{2}d_{2}^{2}+8P^{2}c_{3}d_{1}^{3}d_{2}+P^{2}c_{4}d_{1}^{4}+160\beta^{2}b_{0}^{3}d_{1}d_{5}+256\beta^{2}b_{0}^{3}d_{2}d_{4}+144\beta^{2}b_{0}^{3}d_{3}^{2}\\ +336\beta^{2}b_{0}^{2}b_{1}d_{1}d_{4}+504\beta^{2}b_{0}^{2}b_{1}d_{2}d_{3}+288\beta^{2}b_{0}^{2}b_{2}d_{1}d_{3}+192\beta^{2}b_{0}^{2}b_{2}d_{2}^{2}+216\beta^{2}b_{0}^{2}b_{3}d_{1}d_{2}\\ +60\beta^{2}b_{0}^{2}b_{4}d_{1}^{2}-80\beta^{2}b_{0}^{2}c_{0}d_{1}d_{5}-128\beta^{2}b_{0}^{2}c_{0}d_{2}d_{4}-72\beta^{2}b_{0}^{2}c_{0}d_{3}^{2}-64\beta^{2}b_{0}^{2}c_{1}d_{1}d_{4}96\beta^{2}b_{0}^{2}c_{1}d_{2}d_{3}\\ -48\beta^{2}b_{0}^{2}c_{2}d_{1}d_{3}-32\beta^{2}b_{0}^{2}c_{2}d_{2}^{2}-32\beta^{2}b_{0}^{2}c_{3}d_{1}d_{2}-8\beta^{2}b_{0}^{2}c_{4}d_{1}^{2}+120\beta^{2}b_{0}b_{1}^{2}d_{1}d_{3}+80\beta^{2}b_{0}b_{1}^{2}d_{2}^{2}\\ +160\beta^{2}b_{0}b_{1}b_{2}d_{1}d_{2}+40\beta^{2}b_{0}b_{1}b_{3}d_{1}^{2}+20\beta^{2}b_{0}b_{2}^{2}d_{1}^{2}+10P^{2}\beta b_{1}d_{1}d_{5}+16P^{2}\beta b_{1}d_{2}d_{4}+9P^{2}\beta b_{1}d_{3}^{2}\\ -4P^{2}\beta b_{2}d_{1}d_{4}-6P^{2}\beta b_{2}d_{2}d_{3}-12P^{2}\beta b_{3}d_{1}d_{3}-8P^{2}\beta b_{3}d_{2}^{2}-14P^{2}\beta b_{4}d_{1}d_{2}+80\beta b_{0}^{3}d_{1}d_{4}\\ +120\beta b_{0}^{3}d_{2}d_{3}+120\beta b_{0}^{2}b_{1}d_{1}d_{3}+80\beta b_{0}^{2}b_{1}d_{2}^{2}+80\beta b_{0}^{2}b_{2}d_{1}d_{2}+20\beta b_{0}^{2}b_{3}d_{1}^{2}+40\beta b_{0}b_{1}^{2}d_{1}d_{2}\\ +20\beta b_{0}b_{1}b_{2}d_{1}^{2}+10P^{2}b_{0}d_{1}d_{5}+16P^{2}b_{0}d_{2}d_{4}9P^{2}b_{0}d_{3}^{2}-4P^{2}b_{1}d_{1}d_{4}-6P^{2}b_{1}d_{2}d_{3}-12P^{2}b_{2}d_{1}d_{3}\\ -8P^{2}b_{2}d_{2}^{2}-14P^{2}b_{3}d_{1}d_{2}-5P^{2}b_{4}d_{1}^{2}+20P^{2}c_{0}d_{1}d_{5}+32P^{2}c_{0}d_{2}d_{4}+18P^{2}c_{0}d_{3}^{2}+16P^{2}c_{1}d_{1}d_{4}\\ +24P^{2}c_{1}d_{2}d_{3}+12P^{2}c_{2}d_{1}d_{3}+8P^{2}c_{2}d_{2}^{2}+8P^{2}c_{3}d_{1}d_{2}+2P^{2}c_{4}d_{1}^{2}+20\beta^{2}b_{0}^{2}b_{4}-4\beta^{2}b_{0}^{2}c_{4}-5P^{2}b_{4}\\ +P^{2}c_{4}) \end{array}$$

$$\begin{array}{l} b_{6}=\frac{1}{6\beta (-8\beta^{2}b_{0}^{2}d_{1}^{2}+P^{2}d_{1}^{2}-4\beta^{2}b_{0}^{2}+P^{2})}(-96\beta^{2}b_{0}^{2}c_{0}d_{1}^{3}d_{6}-480\beta^{2}b_{0}^{2}c_{0}d_{1}^{2}d_{2}d_{5}\\ -576\beta^{2}b_{0}^{2}c_{0}d_{1}^{2}d_{3}d_{4}-768\beta^{2}b_{0}^{2}c_{0}d_{1}d_{2}^{2}d_{4}-864\beta^{2}b_{0}^{2}c_{0}d_{1}d_{2}d_{3}^{2}-384\beta^{2}b_{0}^{2}c_{0}d_{2}^{3}d_{3}-80\beta^{2}b_{0}^{2}c_{1}d_{1}^{3}d_{5}\\ -384\beta^{2}b_{0}^{2}c_{1}d_{1}^{2}d_{2}d_{4}-216\beta^{2}b_{0}^{2}c_{1}d_{1}^{2}d_{3}^{2}-576\beta^{2}b_{0}^{2}c_{1}d_{1}d_{2}^{2}d_{3}-64\beta^{2}b_{0}^{2}c_{1}d_{2}^{4}-64\beta^{2}b_{0}^{2}c_{2}d_{1}^{3}d_{4}\\ -288\beta^{2}b_{0}^{2}c_{2}d_{1}^{2}d_{2}d_{3}-128\beta^{2}b_{0}^{2}c_{2}d_{1}d_{2}^{3}-48\beta^{2}b_{0}^{2}c_{3}d_{1}^{3}d_{3}-96\beta^{2}b_{0}^{2}c_{3}d_{1}^{2}d_{2}^{2}-32\beta^{2}b_{0}^{2}c_{4}d_{1}^{3}d_{2}\\ -4\beta^{2}b_{0}^{2}c_{5}d_{1}^{4}+216\beta^{3}b_{0}^{2}b_{1}d_{1}d_{6}+360\beta^{3}b_{0}^{2}b_{1}d_{2}d_{5}+432\beta^{3}b_{0}^{2}b_{1}d_{3}d_{4}+240\beta^{3}b_{0}^{2}b_{2}d_{1}d_{5}\\ +384\beta^{3}b_{0}^{2}b_{2}d_{2}d_{4}+216\beta^{3}b_{0}^{2}b_{2}d_{3}^{2}+240\beta^{3}b_{0}^{2}b_{3}d_{1}d_{4}+360\beta^{3}b_{0}^{2}b_{3}d_{2}d_{3}+216\beta^{3}b_{0}^{2}b_{4}d_{1}d_{3}\\ +144\beta^{3}b_{0}^{2}b_{4}d_{2}^{2}+168\beta^{3}b_{0}^{2}b_{5}d_{1}d_{2}+120\beta^{3}b_{0}b_{1}^{2}d_{1}d_{5}+192\beta^{3}b_{0}b_{1}^{2}d_{2}d_{4}+108\beta^{3}b_{0}b_{1}^{2}d_{3}^{2}\\ +192\beta^{3}b_{0}b_{1}b_{2}d_{1}d_{4}+288\beta^{3}b_{0}b_{1}b_{2}d_{2}d_{3}+144\beta^{3}b_{0}b_{1}b_{3}d_{1}d_{3}+96\beta^{3}b_{0}b_{1}b_{3}d_{2}^{2}+96\beta^{3}b_{0}b_{1}b_{4}d_{1}d_{2}\\ +24\beta^{3}b_{0}b_{1}b_{5}d_{1}^{2}+72\beta^{3}b_{0}b_{2}^{2}d_{1}d_{3}+48\beta^{3}b_{0}b_{2}^{2}d_{2}^{2}+96\beta^{3}b_{0}b_{2}b_{3}d_{1}d_{2}+24\beta^{3}b_{0}b_{2}b_{4}d_{1}^{2}+12\beta^{3}b_{0}b_{3}^{2}d_{1}^{2}\\ +24P^{2}c_{0}d_{1}^{3}d_{6}+120P^{2}c_{0}d_{1}^{2}d_{2}d_{5}+144P^{2}c_{0}d_{1}^{2}d_{3}d_{4}+192P^{2}c_{0}d_{1}d_{2}^{2}d_{4}+216P^{2}c_{0}d_{1}d_{2}d_{3}^{2}\\ +20P^{2}c_{1}d_{1}^{3}d_{5}+96P^{2}c_{1}d_{1}^{2}d_{2}d_{4}+54P^{2}c_{1}d_{1}^{2}d_{3}^{2}+144P^{2}c_{1}d_{1}d_{2}^{2}d_{3}+16P^{2}c_{1}d_{2}^{4}+16P^{2}c_{2}d_{1}^{3}d_{4}\\ +72P^{2}c_{2}d_{1}^{2}d_{2}d_{3}+32P^{2}c_{2}d_{1}d_{2}^{3}+12P^{2}c_{3}d_{1}^{3}d_{3}+24P^{2}c_{3}d_{1}^{2}d_{2}^{2}+8P^{2}c_{4}d_{1}^{3}d_{2}+P^{2}c_{5}d_{1}^{4}+216\beta^{2}b_{0}^{3}d_{1}d_{6}\\ +360\beta^{2}b_{0}^{3}d_{2}d_{5}+432\beta^{2}b_{0}^{3}d_{3}d_{4}+480\beta^{2}b_{0}^{2}b_{1}d_{1}d_{5}+768\beta^{2}b_{0}^{2}b_{1}d_{2}d_{4}+432\beta^{2}b_{0}^{2}b_{1}d_{3}^{2}\\ +432\beta^{2}b_{0}^{2}b_{2}d_{1}d_{4}+648\beta^{2}b_{0}^{2}b_{2}d_{2}d_{3}+360\beta^{2}b_{0}^{2}b_{3}d_{1}d_{3}+240\beta^{2}b_{0}^{2}b_{3}d_{2}^{2}+264\beta^{2}b_{0}^{2}b_{4}d_{1}d_{2} \end{array}$$

$$\begin{array}{l} +72\beta^{2}b_{0}^{2}b_{5}d_{1}^{2}-96\beta^{2}b_{0}^{2}c_{0}d_{1}d_{6}-160\beta^{2}b_{0}^{2}c_{0}d_{2}d_{5}-192\beta^{2}b_{0}^{2}c_{0}d_{3}d_{4}-80\beta^{2}b_{0}^{2}c_{1}d_{1}d_{5}\\ -72\beta^{2}b_{0}^{2}c_{1}d_{3}^{2}-64\beta^{2}b_{0}^{2}c_{2}d_{1}d_{4}-96\beta^{2}b_{0}^{2}c_{2}d_{2}d_{3}-48\beta^{2}b_{0}^{2}c_{3}d_{1}d_{3}-32\beta^{2}b_{0}^{2}c_{3}d_{2}^{2}-32\beta^{2}b_{0}^{2}c_{4}d_{1}d_{2}\\ -8\beta^{2}b_{0}^{2}c_{5}d_{1}^{2}+192\beta^{2}b_{0}b_{1}^{2}d_{1}d_{4}+288\beta^{2}b_{0}b_{1}^{2}d_{2}d_{3}+288\beta^{2}b_{0}b_{1}b_{2}d_{1}d_{3}+192\beta^{2}b_{0}b_{1}b_{2}d_{2}^{2}\\ +192\beta^{2}b_{0}b_{1}b_{3}d_{1}d_{2}+48\beta^{2}b_{0}b_{1}b_{4}d_{1}^{2}+96\beta^{2}b_{0}b_{2}^{2}d_{1}d_{2}+48\beta^{2}b_{0}b_{2}b_{3}d_{1}^{2}+18P^{2}\beta b_{1}d_{1}d_{6}\\ +36P^{2}\beta b_{1}d_{3}d_{4}-12P^{2}\beta b_{3}d_{1}d_{4}-18P^{2}\beta b_{3}d_{2}d_{3}-18P^{2}\beta b_{4}d_{1}d_{3}-12P^{2}\beta b_{4}d_{2}^{2}-18P^{2}\beta b_{5}d_{1}d_{2}\\ +120\beta b_{0}^{3}d_{1}d_{5}+192\beta b_{0}^{3}d_{2}d_{4}+108\beta b_{0}^{3}d_{3}^{2}+192\beta b_{0}^{2}b_{1}d_{1}d_{4}+288\beta b_{0}^{2}b_{1}d_{2}d_{3}+144\beta b_{0}^{2}b_{2}d_{1}d_{3}\\ +96\beta b_{0}^{2}b_{2}d_{2}^{2}+96\beta b_{0}^{2}b_{3}d_{1}d_{2}+24\beta b_{0}^{2}b_{4}d_{1}^{2}+72\beta b_{0}b_{1}^{2}d_{1}d_{3}+48\beta b_{0}b_{1}^{2}d_{2}^{2}+96\beta b_{0}b_{1}b_{2}d_{1}d_{2}\\ +24\beta b_{0}b_{1}b_{3}d_{1}^{2}+12\beta b_{0}b_{2}^{2}d_{1}^{2}+18P^{2}b_{0}d_{1}d_{6}+30P^{2}b_{0}d_{2}d_{5}+36P^{2}b_{0}d_{3}d_{4}-12P^{2}b_{2}d_{1}d_{4}\\ -18P^{2}b_{3}d_{1}d_{3}-12P^{2}b_{3}d_{2}^{2}-18P^{2}b_{4}d_{1}d_{2}-6P^{2}b_{5}d_{1}^{2}+24P^{2}c_{0}d_{1}d_{6}+40P^{2}c_{0}d_{2}d_{5}+48P^{2}c_{0}d_{3}d_{4}\\ +20P^{2}c_{1}d_{1}d_{5}+32P^{2}c_{1}d_{2}d_{4}+18P^{2}c_{1}d_{3}^{2}+16P^{2}c_{2}d_{1}d_{4}+24P^{2}c_{2}d_{2}d_{3}+12P^{2}c_{3}d_{1}d_{3}+8P^{2}c_{3}d_{2}^{2}\\ +8P^{2}c_{4}d_{1}d_{2}+2P^{2}c_{5}d_{1}^{2}+24\beta^{2}b_{0}^{2}b_{5}-4\beta^{2}b_{0}^{2}c_{5}-6P^{2}b_{5}+P^{2}c_{5}-18P^{2}b_{2}d_{2}d_{3}+30P^{2}\beta b_{1}d_{2}d_{5}\\ -128\beta^{2}b_{0}^{2}c_{1}d_{2}d_{4}+96P^{2}c_{0}d_{2}^{3}d_{3}) \end{array}$$

$$\begin{array}{l} b_{7}=\frac{1}{7\beta (-8\beta^{2}b_{0}^{2}d_{1}^{2}+P^{2}d_{1}^{2}-4\beta^{2}b_{0}^{2}+P^{2})}(-48\beta^{2}b_{0}^{2}c_{4}d_{1}d_{3}-32\beta^{2}b_{0}^{2}c_{5}d_{1}d_{2}-64\beta^{2}b_{0}^{2}c_{3}d_{1}d_{4}\\ +96P^{2}c_{2}d_{1}^{2}d_{2}d_{4}-128\beta^{2}b_{0}^{2}c_{2}d_{2}d_{4}+72P^{2}c_{3}d_{1}^{2}d_{2}d_{3}-96\beta^{2}b_{0}^{2}c_{3}d_{2}d_{3}-80\beta^{2}b_{0}^{2}c_{2}d_{1}d_{5}\\ +180P^{2}c_{0}d_{1}^{2}d_{3}d_{5}-240\beta^{2}b_{0}^{2}c_{0}d_{3}d_{5}+144P^{2}c_{1}d_{1}^{2}d_{3}d_{4}-192\beta^{2}b_{0}^{2}c_{1}d_{3}d_{4}-112\beta^{2}b_{0}^{2}c_{0}d_{1}d_{7}\\ +144P^{2}c_{0}d_{1}^{2}d_{2}d_{6}-192\beta^{2}b_{0}^{2}c_{0}d_{2}d_{6}-96\beta^{2}b_{0}^{2}c_{1}d_{1}d_{6}+120P^{2}c_{1}d_{1}^{2}d_{2}d_{5}-160\beta^{2}b_{0}^{2}c_{1}d_{2}d_{5}\\ -128\beta^{2}b_{0}^{2}c_{3}d_{1}d_{2}^{3}+168\beta^{3}b_{0}b_{1}^{2}d_{1}d_{6}+280\beta^{3}b_{0}b_{1}^{2}d_{2}d_{5}+336\beta^{3}b_{0}b_{1}^{2}d_{3}d_{4}+252\beta^{3}b_{0}b_{1}b_{2}d_{3}^{2}\\ +112\beta^{3}b_{0}b_{1}b_{4}d_{2}^{2}+28\beta^{3}b_{0}b_{1}b_{6}d_{1}^{2}+112\beta^{3}b_{0}b_{2}^{2}d_{1}d_{4}+168\beta^{3}b_{0}b_{2}^{2}d_{2}d_{3}+112\beta^{3}b_{0}b_{2}b_{3}d_{2}^{2}\\ +28\beta^{3}b_{0}b_{2}b_{5}d_{1}^{2}+56\beta^{3}b_{0}b_{3}^{2}d_{1}d_{2}+28\beta^{3}b_{0}b_{3}b_{4}d_{1}^{2}+280\beta^{2}b_{0}b_{1}^{2}d_{1}d_{5}+448\beta^{2}b_{0}b_{1}^{2}d_{2}d_{4}\\ +224\beta^{2}b_{0}b_{1}b_{3}d_{2}^{2}+56\beta^{2}b_{0}b_{1}b_{5}d_{1}^{2}-7P^{2}b_{6}+P^{2}c_{6}+336\beta^{2}b_{0}b_{1}b_{3}d_{1}d_{3}+224\beta^{2}b_{0}b_{1}b_{4}d_{1}d_{2}\\ +224\beta^{2}b_{0}b_{2}b_{3}d_{1}d_{2}+168\beta b_{0}b_{1}b_{2}d_{1}d_{3}+112\beta b_{0}b_{1}b_{3}d_{1}d_{2}+576P^{2}c_{0}d_{1}d_{2}d_{3}d_{4}-960\beta^{2}b_{0}^{2}c_{0}d_{1}d_{2}^{2}d_{5}\\ -576\beta^{2}b_{0}^{2}c_{2}d_{1}d_{2}^{2}d_{3}-864\beta^{2}b_{0}^{2}c_{1}d_{1}d_{2}d_{3}^{2}-768\beta^{2}b_{0}^{2}c_{1}d_{1}d_{2}^{2}d_{4}-384\beta^{2}b_{0}^{2}d_{1}^{2}c_{2}d_{2}d_{4}\\ -720\beta^{2}b_{0}^{2}d_{1}^{2}c_{0}d_{3}d_{5}-576\beta^{2}b_{0}^{2}d_{1}^{2}c_{1}d_{3}d_{4}-576\beta^{2}b_{0}^{2}d_{1}^{2}c_{0}d_{2}d_{6}-480\beta^{2}b_{0}^{2}d_{1}^{2}c_{1}d_{2}d_{5}\\ +448\beta^{3}b_{0}b_{1}b_{2}d_{2}d_{4}+224\beta^{3}b_{0}b_{1}b_{3}d_{1}d_{4}+336\beta^{3}b_{0}b_{1}b_{3}d_{2}d_{3}+168\beta^{3}b_{0}b_{1}b_{4}d_{1}d_{3}+112\beta^{3}b_{0}b_{1}b_{5}d_{1}d_{2}\\ +168\beta^{3}b_{0}b_{2}b_{3}d_{1}d_{3}+112\beta^{3}b_{0}b_{2}b_{4}d_{1}d_{2}+448\beta^{2}b_{0}b_{1}b_{2}d_{1}d_{4}+672\beta^{2}b_{0}b_{1}b_{2}d_{2}d_{3}+16P^{2}c_{2}d_{2}^{4}\\ +32P^{2}c_{0}d_{4}^{2}-2304\beta^{2}b_{0}^{2}c_{0}d_{1}d_{2}d_{3}d_{4}+168\beta^{2}b_{0}b_{2}^{2}d_{1}d_{3}+56\beta^{2}b_{0}b_{2}b_{4}d_{1}^{2}+280\beta b_{0}^{2}b_{1}d_{1}d_{5}\\ +448\beta b_{0}^{2}b_{1}d_{2}d_{4}+224\beta b_{0}^{2}b_{2}d_{1}d_{4}+336\beta b_{0}^{2}b_{2}d_{2}d_{3}+168\beta b_{0}^{2}b_{3}d_{1}d_{3}+112\beta b_{0}^{2}b_{4}d_{1}d_{2}\\ -216\beta^{2}b_{0}^{2}d_{1}^{2}c_{2}d_{3}^{2}-96\beta^{2}b_{0}^{2}d_{1}^{2}c_{4}d_{2}^{2}+280\beta^{3}b_{0}^{2}b_{1}d_{1}d_{7}+480\beta^{3}b_{0}^{2}b_{1}d_{2}d_{6}+600\beta^{3}b_{0}^{2}b_{1}d_{3}d_{5}\\ +312\beta^{3}b_{0}^{2}b_{2}d_{1}d_{6}+520\beta^{3}b_{0}^{2}b_{2}d_{2}d_{5}+624\beta^{3}b_{0}^{2}b_{2}d_{3}d_{4}+320\beta^{3}b_{0}^{2}b_{3}d_{1}d_{5}+512\beta^{3}b_{0}^{2}b_{3}d_{2}d_{4}\\ +304\beta^{3}b_{0}^{2}b_{4}d_{1}d_{4}+456\beta^{3}b_{0}^{2}b_{4}d_{2}d_{3}+264\beta^{3}b_{0}^{2}b_{5}d_{1}d_{3}+200\beta^{3}b_{0}^{2}b_{6}d_{1}d_{2}+648\beta^{2}b_{0}^{2}b_{1}d_{1}d_{6}\\ +1080\beta^{2}b_{0}^{2}b_{1}d_{2}d_{5}+1296\beta^{2}b_{0}^{2}b_{1}d_{3}d_{4}+600\beta^{2}b_{0}^{2}b_{2}d_{1}d_{5}+960\beta^{2}b_{0}^{2}b_{2}d_{2}d_{4}+528\beta^{2}b_{0}^{2}b_{3}d_{1}d_{4}\\ +792\beta^{2}b_{0}^{2}b_{3}d_{2}d_{3}+432\beta^{2}b_{0}^{2}b_{4}d_{1}d_{3}+312\beta^{2}b_{0}^{2}b_{5}d_{1}d_{2}-384\beta^{2}b_{0}^{2}c_{1}d_{2}^{3}d_{3}-864\beta^{2}b_{0}^{2}c_{0}d_{2}^{2}d_{3}^{2}\\ -512\beta^{2}b_{0}^{2}c_{0}d_{2}^{3}d_{4}-432\beta^{2}b_{0}^{2}c_{0}d_{1}d_{3}^{3}+240P^{2}c_{0}d_{1}d_{2}^{2}d_{5}-24P^{2}\beta b_{5}d_{1}d_{3}-30P^{2}\beta b_{4}d_{2}d_{3}\\ -22P^{2}\beta b_{6}d_{1}d_{2}-20P^{2}\beta b_{4}d_{1}d_{4}-16P^{2}\beta b_{3}d_{2}d_{4}-10P^{2}\beta b_{3}d_{1}d_{5}+12P^{2}\beta b_{2}d_{3}d_{4}+10P^{2}\beta b_{2}d_{2}d_{5}\\ +6P^{2}\beta b_{2}d_{1}d_{6}+60P^{2}\beta b_{1}d_{3}d_{5}+48P^{2}\beta b_{1}d_{2}d_{6}+28P^{2}\beta b_{1}d_{1}d_{7}+144P^{2}c_{2}d_{1}d_{2}^{2}d_{3}+216P^{2}c_{1}d_{1}d_{2}d_{3}^{2}\\ +192P^{2}c_{1}d_{1}d_{2}^{2}d_{4}+112\beta b_{0}b_{1}^{2}d_{1}d_{4}+168\beta b_{0}b_{1}^{2}d_{2}d_{3}+112\beta b_{0}b_{1}b_{2}d_{2}^{2}+28\beta b_{0}b_{1}b_{4}d_{1}^{2}+56\beta b_{0}b_{2}^{2}d_{1}d_{2}\\ +28\beta b_{0}b_{2}b_{3}d_{1}^{2}-384\beta^{2}b_{0}^{2}d_{1}^{2}c_{0}d_{4}^{2}-48\beta^{2}b_{0}^{2}d_{1}^{3}c_{4}d_{3}-32\beta^{2}b_{0}^{2}d_{1}^{3}c_{5}d_{2}-64\beta^{2}b_{0}^{2}d_{1}^{3}c_{3}d_{4}\\ -80\beta^{2}b_{0}^{2}d_{1}^{3}c_{2}d_{5}-112\beta^{2}b_{0}^{2}d_{1}^{3}c_{0}d_{7}-96\beta^{2}b_{0}^{2}d_{1}^{3}c_{1}d_{6}-24P^{2}b_{4}d_{1}d_{3}-22P^{2}b_{5}d_{1}d_{2}+96P^{2}c_{1}d_{2}^{3}d_{3}\\ +216P^{2}c_{0}d_{2}^{2}d_{3}^{2}-9P^{2}\beta b_{3}d_{3}^{2}-16P^{2}\beta b_{5}d_{2}^{2}+128P^{2}c_{0}d_{2}^{3}d_{4}-10P^{2}b_{2}d_{1}d_{5}+10P^{2}b_{1}d_{2}d_{5}\\ +6P^{2}b_{1}d_{1}d_{6}+12P^{2}c_{4}d_{1}^{3}d_{3}+12P^{2}c_{4}d_{1}d_{3}+8P^{2}c_{5}d_{1}^{3}d_{2}+8P^{2}c_{5}d_{1}d_{2}+48P^{2}b_{0}d_{2}d_{6}+16P^{2}c_{3}d_{1}^{3}d_{4}\\ +16P^{2}c_{3}d_{1}d_{4}+32P^{2}c_{2}d_{2}d_{4}+24P^{2}c_{3}d_{2}d_{3}+20P^{2}c_{2}d_{1}^{3}d_{5}+20P^{2}c_{2}d_{1}d_{5}60P^{2}c_{0}d_{3}d_{5}+48P^{2}c_{1}d_{3}d_{4}\\ +28P^{2}c_{0}d_{1}^{3}d_{7}+28P^{2}c_{0}d_{1}d_{7}+48P^{2}c_{0}d_{2}d_{6}+252\beta^{2}b_{0}b_{1}^{2}d_{3}^{2}+112\beta^{2}b_{0}b_{2}^{2}d_{2}^{2}+28\beta^{2}b_{0}b_{3}^{2}d_{1}^{2} \end{array}$$

$$\begin{array}{l} +168\beta b_{0}^{3}d_{1}d_{6}+280\beta b_{0}^{3}d_{2}d_{5}+336\beta b_{0}^{3}d_{3}d_{4}+252\beta b_{0}^{2}b_{1}d_{3}^{2}+112\beta b_{0}^{2}b_{3}d_{2}^{2}+28\beta b_{0}^{2}b_{5}d_{1}^{2}\\ +320\beta^{3}b_{0}^{2}b_{1}d_{4}^{2}+288\beta^{3}b_{0}^{2}b_{3}d_{3}^{2}+176\beta^{3}b_{0}^{2}b_{5}d_{2}^{2}+280\beta^{2}b_{0}^{3}d_{1}d_{7}+480\beta^{2}b_{0}^{3}d_{2}d_{6}+600\beta^{2}b_{0}^{3}d_{3}d_{5}\\ +540\beta^{2}b_{0}^{2}b_{2}d_{3}^{2}+288\beta^{2}b_{0}^{2}b_{4}d_{2}^{2}+84\beta^{2}b_{0}^{2}b_{6}d_{1}^{2}-4\beta^{2}b_{0}^{2}d_{1}^{4}c_{6}-8\beta^{2}b_{0}^{2}d_{1}^{2}c_{6}+96P^{2}c_{0}d_{1}^{2}d_{4}^{2}\\ -72\beta^{2}b_{0}^{2}c_{2}d_{3}^{2}-32\beta^{2}b_{0}^{2}c_{4}d_{2}^{2}-64\beta^{2}b_{0}^{2}c_{2}d_{2}^{4}-128\beta^{2}b_{0}^{2}c_{0}d_{4}^{2}+108P^{2}c_{0}d_{1}d_{3}^{3}+12P^{2}b_{1}d_{3}d_{4}\\ +24P^{2}c_{1}d_{1}^{3}d_{6}+24P^{2}c_{1}d_{1}d_{6}+40P^{2}c_{1}d_{2}d_{5}+54P^{2}c_{2}d_{1}^{2}d_{3}^{2}+32P^{2}c_{3}d_{1}d_{2}^{3}+24P^{2}c_{4}d_{1}^{2}d_{2}^{2}\\ +32P^{2}\beta b_{1}d_{4}^{2}-16P^{2}b_{2}d_{2}d_{4}-30P^{2}b_{3}d_{2}d_{3}-20P^{2}b_{3}d_{1}d_{4}+320\beta^{2}b_{0}^{3}d_{4}^{2}-7P^{2}b_{6}d_{1}^{2}+28\beta^{2}b_{0}^{2}b_{6}\\ +2P^{2}c_{6}d_{1}^{2}-4\beta^{2}b_{0}^{2}c_{6}+32P^{2}b_{0}d_{4}^{2}-9P^{2}b_{2}d_{3}^{2}-16P^{2}b_{4}d_{2}^{2}+18P^{2}c_{2}d_{3}^{2}+8P^{2}c_{4}d_{2}^{2}+P^{2}c_{6}d_{1}^{4}\\ +28P^{2}b_{0}d_{1}d_{7}+60P^{2}b_{0}d_{3}d_{5}+280\beta^{3}b_{0}b_{1}b_{2}d_{1}d_{5}-288\beta^{2}b_{0}^{2}d_{1}^{2}c_{3}d_{2}d_{3}) \end{array}$$

$$\begin{array}{l} b_{8}=\frac{1}{8\beta (-8\beta^{2}b_{0}^{2}d_{1}^{2}+P^{2}d_{1}^{2}-4\beta^{2}b_{0}^{2}+P^{2})}(54P^{2}c_{3}d_{1}^{2}d_{3}^{2}-72\beta^{2}b_{0}^{2}c_{3}d_{3}^{2}+24P^{2}c_{5}d_{1}^{2}d_{2}^{2}\\ -32\beta^{2}b_{0}^{2}c_{5}d_{2}^{2}-64\beta^{2}b_{0}^{2}c_{3}d_{2}^{4}+96P^{2}c_{1}d_{1}^{2}d_{4}^{2}-128\beta^{2}b_{0}^{2}c_{1}d_{4}^{2}+40P^{2}c_{2}d_{2}d_{5}+72P^{2}c_{0}d_{3}d_{6}\\ +80P^{2}c_{0}d_{4}d_{5}+16P^{2}\beta b_{2}d_{4}^{2}+8P^{2}c_{6}d_{1}^{3}d_{2}+8P^{2}c_{6}d_{1}d_{2}-18P^{2}\beta b_{4}d_{3}^{2}-20P^{2}\beta b_{6}d_{2}^{2}+48P^{2}c_{2}d_{3}d_{4}\\ +16P^{2}c_{4}d_{1}^{3}d_{4}+16P^{2}c_{4}d_{1}d_{4}+20P^{2}c_{3}d_{1}^{3}d_{5}+20P^{2}c_{3}d_{1}d_{5}+14P^{2}b_{1}d_{1}d_{7}+90P^{2}b_{0}d_{3}d_{6}\\ +70P^{2}b_{0}d_{2}d_{7}+40P^{2}b_{0}d_{1}d_{8}+24P^{2}c_{2}d_{1}^{3}d_{6}+24P^{2}c_{2}d_{1}d_{6}+96P^{2}c_{2}d_{2}^{3}d_{3}+216P^{2}c_{1}d_{2}^{2}d_{3}^{2}\\ +108P^{2}c_{1}d_{1}d_{3}^{3}+128P^{2}c_{1}d_{2}^{3}d_{4}+216P^{2}c_{0}d_{2}d_{3}^{3}+160P^{2}c_{0}d_{2}^{3}d_{5}+32P^{2}c_{0}d_{1}^{3}d_{8}+32P^{2}c_{0}d_{1}d_{8}\\ +60P^{2}c_{1}d_{3}d_{5}+12P^{2}c_{5}d_{1}^{3}d_{3}+12P^{2}c_{5}d_{1}d_{3}+56P^{2}c_{0}d_{2}d_{7}+48P^{2}c_{1}d_{2}d_{6}+28P^{2}c_{1}d_{1}^{3}d_{7}\\ +24P^{2}c_{4}d_{2}d_{3}+32P^{2}c_{3}d_{2}d_{4}-26P^{2}b_{6}d_{1}d_{2}-30P^{2}b_{5}d_{1}d_{3}-42P^{2}b_{4}d_{2}d_{3}+P^{2}c_{7}\\ -2880\beta^{2}b_{0}^{2}c_{0}d_{1}d_{2}d_{3}d_{5}-2304\beta^{2}b_{0}^{2}c_{1}d_{1}d_{2}d_{3}d_{4}+384P^{2}c_{0}d_{1}d_{2}d_{4}^{2}+288P^{2}c_{0}d_{1}d_{2}^{2}d_{6}\\ +216P^{2}c_{0}d_{1}^{2}d_{3}d_{6}-160\beta^{2}b_{0}^{2}c_{2}d_{2}d_{5}-288\beta^{2}b_{0}^{2}c_{0}d_{3}d_{6}-320\beta^{2}b_{0}^{2}c_{0}d_{4}d_{5}-32\beta^{2}b_{0}^{2}c_{6}d_{1}d_{2}\\ -192\beta^{2}b_{0}^{2}c_{2}d_{3}d_{4}-64\beta^{2}b_{0}^{2}c_{4}d_{1}d_{4}-80\beta^{2}b_{0}^{2}c_{3}d_{1}d_{5}-96\beta^{2}b_{0}^{2}c_{2}d_{1}d_{6}-128\beta^{2}b_{0}^{2}c_{0}d_{1}d_{8}\\ -240\beta^{2}b_{0}^{2}c_{1}d_{3}d_{5}-48\beta^{2}b_{0}^{2}c_{5}d_{1}d_{3}-224\beta^{2}b_{0}^{2}c_{0}d_{2}d_{7}-192\beta^{2}b_{0}^{2}c_{1}d_{2}d_{6}-112\beta^{2}b_{0}^{2}c_{1}d_{1}d_{7}\\ -96\beta^{2}b_{0}^{2}c_{4}d_{2}d_{3}-128\beta^{2}b_{0}^{2}c_{3}d_{2}d_{4}-128\beta^{2}b_{0}^{2}c_{4}d_{1}d_{2}^{3}-384\beta^{2}b_{0}^{2}c_{2}d_{2}^{3}d_{3}+352\beta^{3}b_{0}^{2}b_{1}d_{1}d_{8}\\ +616\beta^{3}b_{0}^{2}b_{1}d_{2}d_{7}+792\beta^{3}b_{0}^{2}b_{1}d_{3}d_{6}+880\beta^{3}b_{0}^{2}b_{1}d_{4}d_{5}+392\beta^{3}b_{0}^{2}b_{2}d_{1}d_{7}+672\beta^{3}b_{0}^{2}b_{2}d_{2}d_{6}\\ +840\beta^{3}b_{0}^{2}b_{2}d_{3}d_{5}+408\beta^{3}b_{0}^{2}b_{3}d_{1}d_{6}+680\beta^{3}b_{0}^{2}b_{3}d_{2}d_{5}+816\beta^{3}b_{0}^{2}b_{3}d_{3}d_{4}+400\beta^{3}b_{0}^{2}b_{4}d_{1}d_{5}\\ +640\beta^{3}b_{0}^{2}b_{4}d_{2}d_{4}+368\beta^{3}b_{0}^{2}b_{5}d_{1}d_{4}+552\beta^{3}b_{0}^{2}b_{5}d_{2}d_{3}+312\beta^{3}b_{0}^{2}b_{6}d_{1}d_{3}+232\beta^{3}b_{0}^{2}b_{7}d_{1}d_{2}\\ +840\beta^{2}b_{0}^{2}b_{1}d_{1}d_{7}+1440\beta^{2}b_{0}^{2}b_{1}d_{2}d_{6}+1800\beta^{2}b_{0}^{2}b_{1}d_{3}d_{5}+792\beta^{2}b_{0}^{2}b_{2}d_{1}d_{6}+1320\beta^{2}b_{0}^{2}b_{2}d_{2}d_{5}\\ +1584\beta^{2}b_{0}^{2}b_{2}d_{3}d_{4}+720\beta^{2}b_{0}^{2}b_{3}d_{1}d_{5}+1152\beta^{2}b_{0}^{2}b_{3}d_{2}d_{4}+624\beta^{2}b_{0}^{2}b_{4}d_{1}d_{4}+936\beta^{2}b_{0}^{2}b_{4}d_{2}d_{3}\\ +504\beta^{2}b_{0}^{2}b_{5}d_{1}d_{3}+360\beta^{2}b_{0}^{2}b_{6}d_{1}d_{2}-32\beta^{2}b_{0}^{2}d_{1}^{3}c_{6}d_{2}-64\beta^{2}b_{0}^{2}d_{1}^{3}c_{4}d_{4}-80\beta^{2}b_{0}^{2}d_{1}^{3}c_{3}d_{5}\\ -96\beta^{2}b_{0}^{2}d_{1}^{3}c_{2}d_{6}-128\beta^{2}b_{0}^{2}d_{1}^{3}c_{0}d_{8}-48\beta^{2}b_{0}^{2}d_{1}^{3}c_{5}d_{3}-112\beta^{2}b_{0}^{2}d_{1}^{3}c_{1}d_{7}-216\beta^{2}b_{0}^{2}d_{1}^{2}c_{3}d_{3}^{2}\\ -96\beta^{2}b_{0}^{2}d_{1}^{2}c_{5}d_{2}^{2}-384\beta^{2}b_{0}^{2}d_{1}^{2}c_{1}d_{4}^{2}+160\beta b_{0}b_{1}^{2}d_{1}d_{5}+256\beta b_{0}b_{1}^{2}d_{2}d_{4}+128\beta b_{0}b_{1}b_{3}d_{2}^{2}\\ +96\beta b_{0}b_{2}^{2}d_{1}d_{3}+32\beta b_{0}b_{2}b_{4}d_{1}^{2}+224\beta^{3}b_{0}b_{1}^{2}d_{1}d_{7}+384\beta^{3}b_{0}b_{1}^{2}d_{2}d_{6}+480\beta^{3}b_{0}b_{1}^{2}d_{3}d_{5}\\ +288\beta^{3}b_{0}b_{1}b_{3}d_{3}^{2}+128\beta^{3}b_{0}b_{1}b_{5}d_{2}^{2}+32\beta^{3}b_{0}b_{1}b_{7}d_{1}^{2}+160\beta^{3}b_{0}b_{2}^{2}d_{1}d_{5}+256\beta^{3}b_{0}b_{2}^{2}d_{2}d_{4}\\ +128\beta^{3}b_{0}b_{2}b_{4}d_{2}^{2}+32\beta^{3}b_{0}b_{2}b_{6}d_{1}^{2}+96\beta^{3}b_{0}b_{3}^{2}d_{1}d_{3}+32\beta^{3}b_{0}b_{3}b_{5}d_{1}^{2}+384\beta^{2}b_{0}b_{1}^{2}d_{1}d_{6}\\ +640\beta^{2}b_{0}b_{1}^{2}d_{2}d_{5}+768\beta^{2}b_{0}b_{1}^{2}d_{3}d_{4}+576\beta^{2}b_{0}b_{1}b_{2}d_{3}^{2}+256\beta^{2}b_{0}b_{1}b_{4}d_{2}^{2}+64\beta^{2}b_{0}b_{1}b_{6}d_{1}^{2}\\ +256\beta^{2}b_{0}b_{2}^{2}d_{1}d_{4}+384\beta^{2}b_{0}b_{2}^{2}d_{2}d_{3}+256\beta^{2}b_{0}b_{2}b_{3}d_{2}^{2}+64\beta^{2}b_{0}b_{2}b_{5}d_{1}^{2}+128\beta^{2}b_{0}b_{3}^{2}d_{1}d_{2}\\ +64\beta^{2}b_{0}b_{3}b_{4}d_{1}^{2}+384\beta b_{0}^{2}b_{1}d_{1}d_{6}+640\beta b_{0}^{2}b_{1}d_{2}d_{5}+768\beta b_{0}^{2}b_{1}d_{3}d_{4}+320\beta b_{0}^{2}b_{2}d_{1}d_{5}\\ +256\beta b_{0}^{2}b_{3}d_{1}d_{4}+384\beta b_{0}^{2}b_{3}d_{2}d_{3}+192\beta b_{0}^{2}b_{4}d_{1}d_{3}+128\beta b_{0}^{2}b_{5}d_{1}d_{2}+168P^{2}c_{0}d_{1}^{2}d_{2}d_{7}\\ -30P^{2}\beta b_{6}d_{1}d_{3}-12P^{2}\beta b_{3}d_{3}d_{4}-10P^{2}\beta b_{3}d_{2}d_{5}-6P^{2}\beta b_{3}d_{1}d_{6}+90P^{2}\beta b_{1}d_{3}d_{6}-32P^{2}\beta b_{4}d_{2}d_{4}\\ +40P^{2}\beta b_{1}d_{1}d_{8}+70P^{2}\beta b_{1}d_{2}d_{7}+30P^{2}\beta b_{2}d_{3}d_{5}-28P^{2}\beta b_{5}d_{1}d_{4}-42P^{2}\beta b_{5}d_{2}d_{3}+100P^{2}\beta b_{1}d_{4}d_{5}\\ -26P^{2}\beta b_{7}d_{1}d_{2}+14P^{2}\beta b_{2}d_{1}d_{7}+24P^{2}\beta b_{2}d_{2}d_{6}+216P^{2}c_{2}d_{1}d_{2}d_{3}^{2}+96P^{2}c_{3}d_{1}^{2}d_{2}d_{4}\\ +72P^{2}c_{4}d_{1}^{2}d_{2}d_{3}+192P^{2}c_{2}d_{1}d_{2}^{2}d_{4}+144P^{2}c_{2}d_{1}^{2}d_{3}d_{4}+120P^{2}c_{2}d_{1}^{2}d_{2}d_{5}+240P^{2}c_{1}d_{1}d_{2}^{2}d_{5}-8P^{2}b_{7}\\ +192\beta^{3}b_{0}b_{2}b_{4}d_{1}d_{3}+128\beta^{3}b_{0}b_{2}b_{5}d_{1}d_{2}+128\beta^{3}b_{0}b_{3}b_{4}d_{1}d_{2}+640\beta^{2}b_{0}b_{1}b_{2}d_{1}d_{5}+1024\beta^{2}b_{0}b_{1}b_{2}d_{2}d_{4}\\ +512\beta^{2}b_{0}b_{1}b_{3}d_{1}d_{4}+768\beta^{2}b_{0}b_{1}b_{3}d_{2}d_{3}+384\beta^{2}b_{0}b_{1}b_{4}d_{1}d_{3}+256\beta^{2}b_{0}b_{1}b_{5}d_{1}d_{2}+384\beta^{2}b_{0}b_{2}b_{3}d_{1}d_{3} \end{array}$$

$$\begin{array}{l} +256\beta^{2}b_{0}b_{2}b_{4}d_{1}d_{2}-480\beta^{2}b_{0}^{2}d_{1}^{2}c_{2}d_{2}d_{5}-864\beta^{2}b_{0}^{2}d_{1}^{2}c_{0}d_{3}d_{6}-960\beta^{2}b_{0}^{2}d_{1}^{2}c_{0}d_{4}d_{5}\\ -720\beta^{2}b_{0}^{2}d_{1}^{2}c_{1}d_{3}d_{5}-672\beta^{2}b_{0}^{2}d_{1}^{2}c_{0}d_{2}d_{7}-576\beta^{2}b_{0}^{2}d_{1}^{2}c_{1}d_{2}d_{6}-288\beta^{2}b_{0}^{2}d_{1}^{2}c_{4}d_{2}d_{3}\\ +256\beta b_{0}b_{1}b_{2}d_{1}d_{4}+384\beta b_{0}b_{1}b_{2}d_{2}d_{3}+192\beta b_{0}b_{1}b_{3}d_{1}d_{3}+128\beta b_{0}b_{1}b_{4}d_{1}d_{2}+128\beta b_{0}b_{2}b_{3}d_{1}d_{2}\\ -864\beta^{2}b_{0}^{2}c_{2}d_{1}d_{2}d_{3}^{2}-576\beta^{2}b_{0}^{2}c_{3}d_{1}d_{2}^{2}d_{3}-768\beta^{2}b_{0}^{2}c_{2}d_{1}d_{2}^{2}d_{4}-960\beta^{2}b_{0}^{2}c_{1}d_{1}d_{2}^{2}d_{5}\\ -1728\beta^{2}b_{0}^{2}c_{0}d_{1}d_{3}^{2}d_{4}-1536\beta^{2}b_{0}^{2}c_{0}d_{1}d_{2}d_{4}^{2}-1152\beta^{2}b_{0}^{2}c_{0}d_{1}d_{2}^{2}d_{6}+720P^{2}c_{0}d_{1}d_{2}d_{3}d_{5}\\ -20P^{2}\beta b_{4}d_{1}d_{5}-576\beta^{2}b_{0}^{2}d_{1}^{2}c_{2}d_{3}d_{4}-384\beta^{2}b_{0}^{2}d_{1}^{2}c_{3}d_{2}d_{4}-2304\beta^{2}b_{0}^{2}c_{0}d_{2}^{2}d_{3}d_{4}+576P^{2}c_{1}d_{1}d_{2}d_{3}d_{4}\\ +512\beta b_{0}^{2}b_{2}d_{2}d_{4}+32\beta b_{0}b_{1}b_{5}d_{1}^{2}+240P^{2}c_{0}d_{1}^{2}d_{4}d_{5}+28P^{2}c_{1}d_{1}d_{7}+100P^{2}b_{0}d_{4}d_{5}+144P^{2}c_{3}d_{1}d_{2}^{2}d_{3}\\ +384\beta^{3}b_{0}b_{1}b_{2}d_{1}d_{6}+640\beta^{3}b_{0}b_{1}b_{2}d_{2}d_{5}+768\beta^{3}b_{0}b_{1}b_{2}d_{3}d_{4}+320\beta^{3}b_{0}b_{1}b_{3}d_{1}d_{5}+512\beta^{3}b_{0}b_{1}b_{3}d_{2}d_{4}\\ +256\beta^{3}b_{0}b_{1}b_{4}d_{1}d_{4}+384\beta^{3}b_{0}b_{1}b_{4}d_{2}d_{3}+192\beta^{3}b_{0}b_{1}b_{5}d_{1}d_{3}+128\beta^{3}b_{0}b_{1}b_{6}d_{1}d_{2}+256\beta^{3}b_{0}b_{2}b_{3}d_{1}d_{4}\\ +384\beta^{3}b_{0}b_{2}b_{3}d_{2}d_{3}-864\beta^{2}b_{0}^{2}c_{1}d_{2}^{2}d_{3}^{2}-432\beta^{2}b_{0}^{2}c_{1}d_{1}d_{3}^{3}-512\beta^{2}b_{0}^{2}c_{1}d_{2}^{3}d_{4}-864\beta^{2}b_{0}^{2}c_{0}d_{2}d_{3}^{3}\\ -640\beta^{2}b_{0}^{2}c_{0}d_{2}^{3}d_{5}+180P^{2}c_{1}d_{1}^{2}d_{3}d_{5}+144P^{2}c_{1}d_{1}^{2}d_{2}d_{6}+576P^{2}c_{0}d_{2}^{2}d_{3}d_{4}+432P^{2}c_{0}d_{1}d_{3}^{2}d_{4}\\ +256\beta b_{0}^{3}d_{4}^{2}-8P^{2}b_{7}d_{1}^{2}+32\beta^{2}b_{0}^{2}b_{7}+2P^{2}c_{7}d_{1}^{2}-4\beta^{2}b_{0}^{2}c_{7}-18P^{2}b_{3}d_{3}^{2}-20P^{2}b_{5}d_{2}^{2}+16P^{2}b_{1}d_{4}^{2}\\ +18P^{2}c_{3}d_{3}^{2}+8P^{2}c_{5}d_{2}^{2}+P^{2}c_{7}d_{1}^{4}+16P^{2}c_{3}d_{2}^{4}+32P^{2}c_{1}d_{4}^{2}-28P^{2}b_{4}d_{1}d_{4}-32P^{2}b_{3}d_{2}d_{4}-20P^{2}b_{3}d_{1}d_{5}\\ -12P^{2}b_{2}d_{3}d_{4}-10P^{2}b_{2}d_{2}d_{5}-6P^{2}b_{2}d_{1}d_{6}+30P^{2}b_{1}d_{3}d_{5}+24P^{2}b_{1}d_{2}d_{6}+32P^{2}c_{4}d_{1}d_{2}^{3}+256\beta^{3}b_{0}b_{1}^{2}d_{4}^{2}\\ +144\beta^{3}b_{0}b_{2}^{2}d_{3}^{2}+64\beta^{3}b_{0}b_{3}^{2}d_{2}^{2}+16\beta^{3}b_{0}b_{4}^{2}d_{1}^{2}+224\beta b_{0}^{3}d_{1}d_{7}+384\beta b_{0}^{3}d_{2}d_{6}+480\beta b_{0}^{3}d_{3}d_{5}\\ +288\beta b_{0}^{2}b_{2}d_{3}^{2}+128\beta b_{0}^{2}b_{4}d_{2}^{2}+32\beta b_{0}^{2}b_{6}d_{1}^{2}-8\beta^{2}b_{0}^{2}d_{1}^{2}c_{7}-4\beta^{2}b_{0}^{2}d_{1}^{4}c_{7}+144\beta b_{0}b_{1}^{2}d_{3}^{2}+64\beta b_{0}b_{2}^{2}d_{2}^{2}\\ +16\beta b_{0}b_{3}^{2}d_{1}^{2}+448\beta^{3}b_{0}^{2}b_{2}d_{4}^{2}+360\beta^{3}b_{0}^{2}b_{4}d_{3}^{2}+208\beta^{3}b_{0}^{2}b_{6}d_{2}^{2}+352\beta^{2}b_{0}^{3}d_{1}d_{8}+616\beta^{2}b_{0}^{3}d_{2}d_{7}\\ +792\beta^{2}b_{0}^{3}d_{3}d_{6}+880\beta^{2}b_{0}^{3}d_{4}d_{5}+960\beta^{2}b_{0}^{2}b_{1}d_{4}^{2}+648\beta^{2}b_{0}^{2}b_{3}d_{3}^{2}+336\beta^{2}b_{0}^{2}b_{5}d_{2}^{2}+96\beta^{2}b_{0}^{2}b_{7}d_{1}^{2}) \end{array}$$

$$\begin{array}{l} b_{9}=\frac{1}{9\beta (-8\beta^{2}b_{0}^{2}d_{1}^{2}+P^{2}d_{1}^{2}-4\beta^{2}b_{0}^{2}+P^{2})}(96P^{2}c_{2}d_{1}^{2}d_{4}^{2}-128\beta^{2}b_{0}^{2}c_{2}d_{4}^{2}+54P^{2}c_{4}d_{1}^{2}d_{3}^{2}\\ +24P^{2}c_{6}d_{1}^{2}d_{2}^{2}-32\beta^{2}b_{0}^{2}c_{6}d_{2}^{2}-64\beta^{2}b_{0}^{2}c_{4}d_{2}^{4}+150P^{2}c_{0}d_{1}^{2}d_{5}^{2}-200\beta^{2}b_{0}^{2}c_{0}d_{5}^{2}-324\beta^{2}b_{0}^{2}c_{0}d_{3}^{4}\\ +576\beta^{2}b_{0}b_{1}^{2}d_{4}^{2}+324\beta^{2}b_{0}b_{2}^{2}d_{3}^{2}+144\beta^{2}b_{0}b_{3}^{2}d_{2}^{2}+36\beta^{2}b_{0}b_{4}^{2}d_{1}^{2}+288\beta b_{0}^{3}d_{1}d_{8}+504\beta b_{0}^{3}d_{2}d_{7}\\ +648\beta b_{0}^{3}d_{3}d_{6}+720\beta b_{0}^{3}d_{4}d_{5}+576\beta b_{0}^{2}b_{1}d_{4}^{2}+324\beta b_{0}^{2}b_{3}d_{3}^{2}+144\beta b_{0}^{2}b_{5}d_{2}^{2}+36\beta b_{0}^{2}b_{7}d_{1}^{2}\\ +432\beta^{2}b_{0}^{3}d_{1}d_{9}+768\beta^{2}b_{0}^{3}d_{2}d_{8}+1008\beta^{2}b_{0}^{3}d_{3}d_{7}+1152\beta^{2}b_{0}^{3}d_{4}d_{6}+1152\beta^{2}b_{0}^{2}b_{2}d_{4}^{2}+756\beta^{2}b_{0}^{2}b_{4}d_{3}^{2}\\ +384\beta^{2}b_{0}^{2}b_{6}d_{2}^{2}+108\beta^{2}b_{0}^{2}b_{8}d_{1}^{2}+600\beta^{3}b_{0}^{2}b_{1}d_{5}^{2}+576\beta^{3}b_{0}^{2}b_{3}d_{4}^{2}+432\beta^{3}b_{0}^{2}b_{5}d_{3}^{2}+240\beta^{3}b_{0}^{2}b_{7}d_{2}^{2}\\ -8\beta^{2}b_{0}^{2}d_{1}^{2}c_{8}-4\beta^{2}b_{0}^{2}d_{1}^{4}c_{8}+32P^{2}c_{5}d_{1}d_{2}^{3}+108P^{2}c_{2}d_{1}d_{3}^{3}+54P^{2}b_{0}d_{1}d_{9}+96P^{2}b_{0}d_{2}d_{8}\\ +144P^{2}b_{0}d_{4}d_{6}+24P^{2}b_{1}d_{1}d_{8}+40P^{2}c_{3}d_{2}d_{5}+24P^{2}c_{3}d_{1}^{3}d_{6}+24P^{2}c_{3}d_{1}d_{6}+60P^{2}c_{2}d_{3}d_{5}\\ +42P^{2}b_{1}d_{2}d_{7}+54P^{2}b_{1}d_{3}d_{6}+60P^{2}b_{1}d_{4}d_{5}-18P^{2}b_{3}d_{1}d_{6}-30P^{2}b_{3}d_{2}d_{5}+20P^{2}c_{4}d_{1}^{3}d_{5}\\ +96P^{2}c_{3}d_{2}^{3}d_{3}+216P^{2}c_{2}d_{2}^{2}d_{3}^{2}+128P^{2}c_{2}d_{2}^{3}d_{4}+80P^{2}c_{1}d_{4}d_{5}-30P^{2}b_{7}d_{1}d_{2}-36P^{2}b_{6}d_{1}d_{3}\\ -54P^{2}b_{5}d_{2}d_{3}-36P^{2}b_{5}d_{1}d_{4}-48P^{2}b_{4}d_{2}d_{4}-30P^{2}b_{4}d_{1}d_{5}-36P^{2}b_{3}d_{3}d_{4}+8P^{2}c_{7}d_{1}^{3}d_{2}+8P^{2}c_{7}d_{1}d_{2}\\ +12P^{2}c_{6}d_{1}^{3}d_{3}+12P^{2}c_{6}d_{1}d_{3}+24P^{2}c_{5}d_{2}d_{3}+36P^{2}c_{0}d_{1}d_{9}+32P^{2}c_{4}d_{2}d_{4}+16P^{2}c_{5}d_{1}^{3}d_{4}+16P^{2}c_{5}d_{1}d_{4}\\ +64P^{2}c_{0}d_{2}d_{8}+32P^{2}c_{1}d_{1}d_{8}+96P^{2}c_{0}d_{4}d_{6}+56P^{2}c_{1}d_{2}d_{7}+48P^{2}c_{2}d_{2}d_{6}+28P^{2}c_{2}d_{1}^{3}d_{7}+28P^{2}c_{2}d_{1}d_{7}\\ +72P^{2}c_{1}d_{3}d_{6}+84P^{2}c_{0}d_{3}d_{7}+75P^{2}\beta b_{1}d_{5}^{2}-27P^{2}\beta b_{5}d_{3}^{2}-24P^{2}\beta b_{7}d_{2}^{2}+160P^{2}c_{1}d_{2}^{3}d_{5}\\ +32P^{2}c_{1}d_{1}^{3}d_{8}+384P^{2}c_{0}d_{2}^{2}d_{4}^{2}+192P^{2}c_{0}d_{2}^{3}d_{6}+36P^{2}c_{0}d_{1}^{3}d_{9}-32\beta^{2}b_{0}^{2}c_{7}d_{1}d_{2}-48\beta^{2}b_{0}^{2}c_{6}d_{1}d_{3}\\ -96\beta^{2}b_{0}^{2}c_{5}d_{2}d_{3}-144\beta^{2}b_{0}^{2}c_{0}d_{1}d_{9}-128\beta^{2}b_{0}^{2}c_{4}d_{2}d_{4}-64\beta^{2}b_{0}^{2}c_{5}d_{1}d_{4}-256\beta^{2}b_{0}^{2}c_{0}d_{2}d_{8}\\ -384\beta^{2}b_{0}^{2}c_{0}d_{4}d_{6}-224\beta^{2}b_{0}^{2}c_{1}d_{2}d_{7}-192\beta^{2}b_{0}^{2}c_{2}d_{2}d_{6}-112\beta^{2}b_{0}^{2}c_{2}d_{1}d_{7}-288\beta^{2}b_{0}^{2}c_{1}d_{3}d_{6}\\ -336\beta^{2}b_{0}^{2}c_{0}d_{3}d_{7}-160\beta^{2}b_{0}^{2}c_{3}d_{2}d_{5}-96\beta^{2}b_{0}^{2}c_{3}d_{1}d_{6}-240\beta^{2}b_{0}^{2}c_{2}d_{3}d_{5}-192\beta^{2}b_{0}^{2}c_{3}d_{3}d_{4}\\ -80\beta^{2}b_{0}^{2}c_{4}d_{1}d_{5}-384\beta^{2}b_{0}^{2}c_{3}d_{2}^{3}d_{3}-864\beta^{2}b_{0}^{2}c_{2}d_{2}^{2}d_{3}^{2}-512\beta^{2}b_{0}^{2}c_{2}d_{2}^{3}d_{4}-320\beta^{2}b_{0}^{2}c_{1}d_{4}d_{5}\\ +128\beta^{2}b_{0}^{2}c_{5}d_{1}d_{2}^{3}-432\beta^{2}b_{0}^{2}c_{2}d_{1}d_{3}^{3}+54P^{2}\beta b_{1}d_{1}d_{9}+72P^{2}c_{5}d_{1}^{2}d_{2}d_{3}+144P^{2}c_{4}d_{1}d_{2}^{2}d_{3}\\ +216P^{2}c_{3}d_{1}d_{2}d_{3}^{2}+192P^{2}c_{3}d_{1}d_{2}^{2}d_{4}+144P^{2}c_{3}d_{1}^{2}d_{3}d_{4}+336P^{2}c_{0}d_{1}d_{2}^{2}d_{7}+288P^{2}c_{0}d_{1}^{2}d_{4}d_{6}\\ +252P^{2}c_{0}d_{1}^{2}d_{3}d_{7}+192P^{2}c_{0}d_{1}^{2}d_{2}d_{8}+120P^{2}c_{3}d_{1}^{2}d_{2}d_{5}+240P^{2}c_{2}d_{1}d_{2}^{2}d_{5}+180P^{2}c_{2}d_{1}^{2}d_{3}d_{5}\\ +144P^{2}c_{2}d_{1}^{2}d_{2}d_{6}+576P^{2}c_{1}d_{2}^{2}d_{3}d_{4}+432P^{2}c_{1}d_{1}d_{3}^{2}d_{4}+384P^{2}c_{1}d_{1}d_{2}d_{4}^{2}+288P^{2}c_{1}d_{1}d_{2}^{2}d_{6}\\ +240P^{2}c_{1}d_{1}^{2}d_{4}d_{5}-640\beta^{2}b_{0}^{2}c_{1}d_{2}^{3}d_{5}-864\beta^{2}b_{0}^{2}c_{1}d_{2}d_{3}^{3}-1536\beta^{2}b_{0}^{2}c_{0}d_{2}^{2}d_{4}^{2}-768\beta^{2}b_{0}^{2}c_{0}d_{2}^{3}d_{6} \end{array}$$

$$\begin{array}{l} +72\beta^{2}b_{0}b_{3}b_{5}d_{1}^{2}+504\beta b_{0}^{2}b_{1}d_{1}d_{7}+864\beta b_{0}^{2}b_{1}d_{2}d_{6}+1080\beta b_{0}^{2}b_{1}d_{3}d_{5}+432\beta b_{0}^{2}b_{2}d_{1}d_{6}\\ +864\beta b_{0}^{2}b_{2}d_{3}d_{4}+360\beta b_{0}^{2}b_{3}d_{1}d_{5}+576\beta b_{0}^{2}b_{3}d_{2}d_{4}+288\beta b_{0}^{2}b_{4}d_{1}d_{4}+432\beta b_{0}^{2}b_{4}d_{2}d_{3}\\ +144\beta b_{0}^{2}b_{6}d_{1}d_{2}+216P^{2}c_{1}d_{1}^{2}d_{3}d_{6}+168P^{2}c_{1}d_{1}^{2}d_{2}d_{7}+720P^{2}c_{0}d_{2}^{2}d_{3}d_{5}+864P^{2}c_{0}d_{2}d_{3}^{2}d_{4}\\ +576P^{2}c_{0}d_{1}d_{3}d_{4}^{2}+540P^{2}c_{0}d_{1}d_{3}^{2}d_{5}-30P^{2}\beta b_{8}d_{1}d_{2}-36P^{2}\beta b_{7}d_{1}d_{3}-54P^{2}\beta b_{6}d_{2}d_{3}\\ -48P^{2}\beta b_{5}d_{2}d_{4}-30P^{2}\beta b_{5}d_{1}d_{5}-36P^{2}\beta b_{4}d_{3}d_{4}-30P^{2}\beta b_{4}d_{2}d_{5}-18P^{2}\beta b_{4}d_{1}d_{6}\\ +54P^{2}\beta b_{2}d_{3}d_{6}+42P^{2}\beta b_{2}d_{2}d_{7}+24P^{2}\beta b_{2}d_{1}d_{8}+144P^{2}\beta b_{1}d_{4}d_{6}+126P^{2}\beta b_{1}d_{3}d_{7}\\ +288\beta^{3}b_{0}b_{1}^{2}d_{1}d_{8}+504\beta^{3}b_{0}b_{1}^{2}d_{2}d_{7}+648\beta^{3}b_{0}b_{1}^{2}d_{3}d_{6}+720\beta^{3}b_{0}b_{1}^{2}d_{4}d_{5}+576\beta^{3}b_{0}b_{1}b_{2}d_{4}^{2}\\ +324\beta^{3}b_{0}b_{1}b_{4}d_{3}^{2}+144\beta^{3}b_{0}b_{1}b_{6}d_{2}^{2}+36\beta^{3}b_{0}b_{1}b_{8}d_{1}^{2}+216\beta^{3}b_{0}b_{2}^{2}d_{1}d_{6}+360\beta^{3}b_{0}b_{2}^{2}d_{2}d_{5}\\ +432\beta^{3}b_{0}b_{2}^{2}d_{3}d_{4}+324\beta^{3}b_{0}b_{2}b_{3}d_{3}^{2}+144\beta^{3}b_{0}b_{2}b_{5}d_{2}^{2}+36\beta^{3}b_{0}b_{2}b_{7}d_{1}^{2}+144\beta^{3}b_{0}b_{3}^{2}d_{1}d_{4}\\ +216\beta^{3}b_{0}b_{3}^{2}d_{2}d_{3}+144\beta^{3}b_{0}b_{3}b_{4}d_{2}^{2}+36\beta^{3}b_{0}b_{3}b_{6}d_{1}^{2}+72\beta^{3}b_{0}b_{4}^{2}d_{1}d_{2}+36\beta^{3}b_{0}b_{4}b_{5}d_{1}^{2}\\ +864\beta^{2}b_{0}b_{1}^{2}d_{2}d_{6}+1080\beta^{2}b_{0}b_{1}^{2}d_{3}d_{5}+648\beta^{2}b_{0}b_{1}b_{3}d_{3}^{2}+288\beta^{2}b_{0}b_{1}b_{5}d_{2}^{2}+72\beta^{2}b_{0}b_{1}b_{7}d_{1}^{2}\\ +360\beta^{2}b_{0}b_{2}^{2}d_{1}d_{5}+576\beta^{2}b_{0}b_{2}^{2}d_{2}d_{4}+288\beta^{2}b_{0}b_{2}b_{4}d_{2}^{2}+72\beta^{2}b_{0}b_{2}b_{6}d_{1}^{2}+216\beta^{2}b_{0}b_{3}^{2}d_{1}d_{3}\\ +1080\beta^{3}b_{0}^{2}b_{3}d_{3}d_{5}+504\beta^{3}b_{0}^{2}b_{4}d_{1}d_{6}+840\beta^{3}b_{0}^{2}b_{4}d_{2}d_{5}+1008\beta^{3}b_{0}^{2}b_{4}d_{3}d_{4}+480\beta^{3}b_{0}^{2}b_{5}d_{1}d_{5}\\ +768\beta^{3}b_{0}^{2}b_{5}d_{2}d_{4}+432\beta^{3}b_{0}^{2}b_{6}d_{1}d_{4}+648\beta^{3}b_{0}^{2}b_{6}d_{2}d_{3}+360\beta^{3}b_{0}^{2}b_{7}d_{1}d_{3}+264\beta^{3}b_{0}^{2}b_{8}d_{1}d_{2}\\ -72\beta^{2}b_{0}^{2}c_{4}d_{3}^{2}+48P^{2}c_{3}d_{3}d_{4}+720\beta b_{0}^{2}b_{2}d_{2}d_{5}+96P^{2}c_{4}d_{1}^{2}d_{2}d_{4}+216P^{2}c_{1}d_{2}d_{3}^{3}-128\beta^{2}b_{0}^{2}c_{1}d_{1}d_{8}\\ +504\beta^{2}b_{0}b_{1}^{2}d_{1}d_{7}+216\beta b_{0}^{2}b_{5}d_{1}d_{3}+288\beta^{2}b_{0}b_{2}b_{5}d_{1}d_{2}-672\beta^{2}b_{0}^{2}d_{1}^{2}c_{1}d_{2}d_{7}+960P^{2}c_{0}d_{1}d_{2}d_{4}d_{5}\\ +60P^{2}\beta b_{2}d_{4}d_{5}+96P^{2}\beta b_{1}d_{2}d_{8}-36P^{2}\beta b_{6}d_{1}d_{4}+20P^{2}c_{4}d_{1}d_{5}+126P^{2}b_{0}d_{3}d_{7}\\ +216\beta b_{0}b_{1}^{2}d_{1}d_{6}+360\beta b_{0}b_{1}^{2}d_{2}d_{5}+432\beta b_{0}b_{1}^{2}d_{3}d_{4}+324\beta b_{0}b_{1}b_{2}d_{3}^{2}+144\beta b_{0}b_{1}b_{4}d_{2}^{2}+36\beta b_{0}b_{1}b_{6}d_{1}^{2}\\ +144\beta b_{0}b_{2}^{2}d_{1}d_{4}+216\beta b_{0}b_{2}^{2}d_{2}d_{3}+144\beta b_{0}b_{2}b_{3}d_{2}^{2}+36\beta b_{0}b_{2}b_{5}d_{1}^{2}+72\beta b_{0}b_{3}^{2}d_{1}d_{2}+36\beta b_{0}b_{3}b_{4}d_{1}^{2}\\ +1056\beta^{2}b_{0}^{2}b_{1}d_{1}d_{8}+1848\beta^{2}b_{0}^{2}b_{1}d_{2}d_{7}+2376\beta^{2}b_{0}^{2}b_{1}d_{3}d_{6}+2640\beta^{2}b_{0}^{2}b_{1}d_{4}d_{5}+1008\beta^{2}b_{0}^{2}b_{2}d_{1}d_{7}\\ +1728\beta^{2}b_{0}^{2}b_{2}d_{2}d_{6}+2160\beta^{2}b_{0}^{2}b_{2}d_{3}d_{5}+936\beta^{2}b_{0}^{2}b_{3}d_{1}d_{6}+1560\beta^{2}b_{0}^{2}b_{3}d_{2}d_{5}+1872\beta^{2}b_{0}^{2}b_{3}d_{3}d_{4}\\ +840\beta^{2}b_{0}^{2}b_{4}d_{1}d_{5}+1344\beta^{2}b_{0}^{2}b_{4}d_{2}d_{4}+720\beta^{2}b_{0}^{2}b_{5}d_{1}d_{4}+1080\beta^{2}b_{0}^{2}b_{5}d_{2}d_{3}+576\beta^{2}b_{0}^{2}b_{6}d_{1}d_{3}\\ +408\beta^{2}b_{0}^{2}b_{7}d_{1}d_{2}+432\beta^{3}b_{0}^{2}b_{1}d_{1}d_{9}+768\beta^{3}b_{0}^{2}b_{1}d_{2}d_{8}+1008\beta^{3}b_{0}^{2}b_{1}d_{3}d_{7}+1152\beta^{3}b_{0}^{2}b_{1}d_{4}d_{6}\\ +480\beta^{3}b_{0}^{2}b_{2}d_{1}d_{8}+840\beta^{3}b_{0}^{2}b_{2}d_{2}d_{7}+1080\beta^{3}b_{0}^{2}b_{2}d_{3}d_{6}+1200\beta^{3}b_{0}^{2}b_{2}d_{4}d_{5}+504\beta^{3}b_{0}^{2}b_{3}d_{1}d_{7}\\ +864\beta^{3}b_{0}^{2}b_{3}d_{2}d_{6}-32\beta^{2}b_{0}^{2}d_{1}^{3}c_{7}d_{2}-48\beta^{2}b_{0}^{2}d_{1}^{3}c_{6}d_{3}-144\beta^{2}b_{0}^{2}d_{1}^{3}c_{0}d_{9}-64\beta^{2}b_{0}^{2}d_{1}^{3}c_{5}d_{4}\\ -128\beta^{2}b_{0}^{2}d_{1}^{3}c_{1}d_{8}-112\beta^{2}b_{0}^{2}d_{1}^{3}c_{2}d_{7}-384\beta^{2}b_{0}^{2}d_{1}^{2}c_{2}d_{4}^{2}-216\beta^{2}b_{0}^{2}d_{1}^{2}c_{4}d_{3}^{2}-96\beta^{2}b_{0}^{2}d_{1}^{2}c_{6}d_{2}^{2}\\ -600\beta^{2}b_{0}^{2}d_{1}^{2}c_{0}d_{5}^{2}-96\beta^{2}b_{0}^{2}d_{1}^{3}c_{3}d_{6}-80\beta^{2}b_{0}^{2}d_{1}^{3}c_{4}d_{5}+81P^{2}c_{0}d_{3}^{4}+75P^{2}b_{0}d_{5}^{2}+600\beta^{2}b_{0}^{3}d_{5}^{2}\\ -9P^{2}b_{8}d_{1}^{2}+36\beta^{2}b_{0}^{2}b_{8}+2P^{2}c_{8}d_{1}^{2}-4\beta^{2}b_{0}^{2}c_{8}+P^{2}c_{8}d_{1}^{4}+32P^{2}c_{2}d_{4}^{2}+18P^{2}c_{4}d_{3}^{2}+8P^{2}c_{6}d_{2}^{2}\\ +16P^{2}c_{4}d_{2}^{4}+50P^{2}c_{0}d_{5}^{2}-27P^{2}b_{4}d_{3}^{2}-24P^{2}b_{6}d_{2}^{2}-2880\beta^{2}b_{0}^{2}c_{0}d_{2}^{2}d_{3}d_{5}-3456\beta^{2}b_{0}^{2}c_{0}d_{2}d_{3}^{2}d_{4}\\ -2304\beta^{2}b_{0}^{2}c_{0}d_{1}d_{3}d_{4}^{2}-2160\beta^{2}b_{0}^{2}c_{0}d_{1}d_{3}^{2}d_{5}-576\beta^{2}b_{0}^{2}c_{4}d_{1}d_{2}^{2}d_{3}-864\beta^{2}b_{0}^{2}c_{3}d_{1}d_{2}d_{3}^{2}\\ -768\beta^{2}b_{0}^{2}c_{3}d_{1}d_{2}^{2}d_{4}-1344\beta^{2}b_{0}^{2}c_{0}d_{1}d_{2}^{2}d_{7}-960\beta^{2}b_{0}^{2}c_{2}d_{1}d_{2}^{2}d_{5}-2304\beta^{2}b_{0}^{2}c_{1}d_{2}^{2}d_{3}d_{4}\\ -1728\beta^{2}b_{0}^{2}c_{1}d_{1}d_{3}^{2}d_{4}-1536\beta^{2}b_{0}^{2}c_{1}d_{1}d_{2}d_{4}^{2}-1152\beta^{2}b_{0}^{2}c_{1}d_{1}d_{2}^{2}d_{6}-480\beta^{2}b_{0}^{2}d_{1}^{2}c_{3}d_{2}d_{5}\\ -720\beta^{2}b_{0}^{2}d_{1}^{2}c_{2}d_{3}d_{5}-576\beta^{2}b_{0}^{2}d_{1}^{2}c_{3}d_{3}d_{4}-960\beta^{2}b_{0}^{2}d_{1}^{2}c_{1}d_{4}d_{5}+720P^{2}c_{1}d_{1}d_{2}d_{3}d_{5}\\ +864P^{2}c_{0}d_{1}d_{2}d_{3}d_{6}+576P^{2}c_{2}d_{1}d_{2}d_{3}d_{4}+432\beta b_{0}b_{1}b_{3}d_{2}d_{3}+216\beta b_{0}b_{1}b_{4}d_{1}d_{3}+144\beta b_{0}b_{1}b_{5}d_{1}d_{2}\\ +216\beta b_{0}b_{2}b_{3}d_{1}d_{3}+144\beta b_{0}b_{2}b_{4}d_{1}d_{2}+360\beta b_{0}b_{1}b_{2}d_{1}d_{5}+576\beta b_{0}b_{1}b_{2}d_{2}d_{4}+288\beta b_{0}b_{1}b_{3}d_{1}d_{4}\\ -288\beta^{2}b_{0}^{2}d_{1}^{2}c_{5}d_{2}d_{3}-384\beta^{2}b_{0}^{2}d_{1}^{2}c_{4}d_{2}d_{4}-768\beta^{2}b_{0}^{2}d_{1}^{2}c_{0}d_{2}d_{8}-1152\beta^{2}b_{0}^{2}d_{1}^{2}c_{0}d_{4}d_{6}\\ -576\beta^{2}b_{0}^{2}d_{1}^{2}c_{2}d_{2}d_{6}-864\beta^{2}b_{0}^{2}d_{1}^{2}c_{1}d_{3}d_{6}-1008\beta^{2}b_{0}^{2}d_{1}^{2}c_{0}d_{3}d_{7}+432\beta^{2}b_{0}b_{2}b_{4}d_{1}d_{3}\\ +288\beta^{2}b_{0}b_{3}b_{4}d_{1}d_{2}+504\beta^{3}b_{0}b_{1}b_{2}d_{1}d_{7}+864\beta^{3}b_{0}b_{1}b_{2}d_{2}d_{6}+1080\beta^{3}b_{0}b_{1}b_{2}d_{3}d_{5}+432\beta^{3}b_{0}b_{1}b_{3}d_{1}d_{6}\\ +720\beta^{3}b_{0}b_{1}b_{3}d_{2}d_{5}+864\beta^{3}b_{0}b_{1}b_{3}d_{3}d_{4}+360\beta^{3}b_{0}b_{1}b_{4}d_{1}d_{5}+576\beta^{3}b_{0}b_{1}b_{4}d_{2}d_{4}+288\beta^{3}b_{0}b_{1}b_{5}d_{1}d_{4}\\ +432\beta^{3}b_{0}b_{1}b_{5}d_{2}d_{3}+216\beta^{3}b_{0}b_{1}b_{6}d_{1}d_{3}+144\beta^{3}b_{0}b_{1}b_{7}d_{1}d_{2}+360\beta^{3}b_{0}b_{2}b_{3}d_{1}d_{5}+576\beta^{3}b_{0}b_{2}b_{3}d_{2}d_{4}\\ +288\beta^{3}b_{0}b_{2}b_{4}d_{1}d_{4}+432\beta^{3}b_{0}b_{2}b_{4}d_{2}d_{3}+216\beta^{3}b_{0}b_{2}b_{5}d_{1}d_{3}+144\beta^{3}b_{0}b_{2}b_{6}d_{1}d_{2}+216\beta^{3}b_{0}b_{3}b_{4}d_{1}d_{3}\\ +144\beta^{3}b_{0}b_{3}b_{5}d_{1}d_{2}+864\beta^{2}b_{0}b_{1}b_{2}d_{1}d_{6}+1440\beta^{2}b_{0}b_{1}b_{2}d_{2}d_{5}+1728\beta^{2}b_{0}b_{1}b_{2}d_{3}d_{4}+720\beta^{2}b_{0}b_{1}b_{3}d_{1}d_{5} \end{array}$$

$$\begin{array}{l} +1152\beta^{2}b_{0}b_{1}b_{3}d_{2}d_{4}+576\beta^{2}b_{0}b_{1}b_{4}d_{1}d_{4}+864\beta^{2}b_{0}b_{1}b_{4}d_{2}d_{3}+432\beta^{2}b_{0}b_{1}b_{5}d_{1}d_{3}+288\beta^{2}b_{0}b_{1}b_{6}d_{1}d_{2}\\ +576\beta^{2}b_{0}b_{2}b_{3}d_{1}d_{4}+864\beta^{2}b_{0}b_{2}b_{3}d_{2}d_{3}-2880\beta^{2}b_{0}^{2}c_{1}d_{1}d_{2}d_{3}d_{5}-3840\beta^{2}b_{0}^{2}c_{0}d_{1}d_{2}d_{4}d_{5}\\ -3456\beta^{2}b_{0}^{2}c_{0}d_{1}d_{2}d_{3}d_{6}-2304\beta^{2}b_{0}^{2}c_{2}d_{1}d_{2}d_{3}d_{4}+P^{2}c_{8}-9P^{2}b_{8}) \end{array}$$

$$\begin{array}{l} b_{10}=\frac{1}{10\beta (-8\beta^{2}b_{0}^{2}d_{1}^{2}+P^{2}d_{1}^{2}-4\beta^{2}b_{0}^{2}+P^{2})}(-2880\beta^{2}b_{0}^{2}c_{1}d_{2}^{2}d_{3}d_{5}-2304\beta^{2}b_{0}^{2}c_{1}d_{1}d_{3}d_{4}^{2}\\ -2160\beta^{2}b_{0}^{2}c_{1}d_{1}d_{3}^{2}d_{5}-1344\beta^{2}b_{0}^{2}c_{1}d_{1}d_{2}^{2}d_{7}-4608\beta^{2}b_{0}^{2}c_{0}d_{2}d_{3}d_{4}^{2}-4320\beta^{2}b_{0}^{2}c_{0}d_{2}d_{3}^{2}d_{5}+500\beta b_{0}^{3}d_{5}^{2}\\ -10P^{2}b_{9}d_{1}^{2}+40\beta^{2}b_{0}^{2}b_{9}+2P^{2}c_{9}d_{1}^{2}-4\beta^{2}b_{0}^{2}c_{9}+8P^{2}c_{7}d_{2}^{2}+P^{2}c_{9}d_{1}^{4}+50P^{2}c_{1}d_{5}^{2}+32P^{2}c_{3}d_{4}^{2}\\ +18P^{2}c_{5}d_{3}^{2}-16P^{2}b_{3}d_{4}^{2}-36P^{2}b_{5}d_{3}^{2}-28P^{2}b_{7}d_{2}^{2}+81P^{2}c_{1}d_{3}^{4}+16P^{2}c_{5}d_{2}^{4}+50P^{2}b_{1}d_{5}^{2}\\ -32\beta^{2}b_{0}^{2}c_{7}d_{2}^{2}+150P^{2}c_{1}d_{1}^{2}d_{5}^{2}-200\beta^{2}b_{0}^{2}c_{1}d_{5}^{2}+96P^{2}c_{3}d_{1}^{2}d_{4}^{2}-128\beta^{2}b_{0}^{2}c_{3}d_{4}^{2}+54P^{2}c_{5}d_{1}^{2}d_{3}^{2}\\ -72\beta^{2}b_{0}^{2}c_{5}d_{3}^{2}-324\beta^{2}b_{0}^{2}c_{1}d_{3}^{4}-64\beta^{2}b_{0}^{2}c_{5}d_{2}^{4}+96P^{2}c_{0}d_{3}d_{8}+72P^{2}c_{0}d_{2}d_{9}+40P^{2}c_{0}d_{1}^{3}d_{10}\\ +40P^{2}c_{0}d_{1}d_{10}+8P^{2}c_{8}d_{1}^{3}d_{2}+12P^{2}c_{7}d_{1}^{3}d_{3}+32P^{2}c_{6}d_{1}d_{2}^{3}+16P^{2}c_{6}d_{1}^{3}d_{4}+20P^{2}c_{5}d_{1}^{3}d_{5}\\ +24P^{2}c_{4}d_{1}^{3}d_{6}+216P^{2}c_{3}d_{2}^{2}d_{3}^{2}+128P^{2}c_{3}d_{2}^{3}d_{4}+108P^{2}c_{3}d_{1}d_{3}^{3}+28P^{2}c_{3}d_{1}^{3}d_{7}+216P^{2}c_{2}d_{2}d_{3}^{3}\\ +160P^{2}c_{2}d_{2}^{3}d_{5}+32P^{2}c_{2}d_{1}^{3}d_{8}+384P^{2}c_{1}d_{2}^{2}d_{4}^{2}+192P^{2}c_{1}d_{2}^{3}d_{6}+36P^{2}c_{1}d_{1}^{3}d_{9}+432P^{2}c_{0}d_{3}^{3}d_{4}\\ +224P^{2}c_{0}d_{2}^{3}d_{7}+256P^{2}c_{0}d_{1}d_{4}^{3}+210P^{2}b_{0}d_{5}d_{6}+36P^{2}b_{1}d_{1}d_{9}+50P^{2}\beta b_{2}d_{5}^{2}-16P^{2}\beta b_{4}d_{4}^{2}\\ +70P^{2}b_{0}d_{1}d_{10}-36P^{2}\beta b_{6}d_{3}^{2}-28P^{2}\beta b_{8}d_{2}^{2}-34P^{2}b_{8}d_{1}d_{2}-42P^{2}b_{7}d_{1}d_{3}-40P^{2}b_{5}d_{1}d_{5}\\ -44P^{2}b_{6}d_{1}d_{4}-66P^{2}b_{6}d_{2}d_{3}-24P^{2}b_{3}d_{2}d_{6}-60P^{2}b_{4}d_{3}d_{4}-30P^{2}b_{4}d_{1}d_{6}-50P^{2}b_{4}d_{2}d_{5}\\ -14P^{2}b_{3}d_{1}d_{7}+20P^{2}b_{2}d_{4}d_{5}+126P^{2}b_{0}d_{2}d_{9}+64P^{2}b_{1}d_{2}d_{8}+196P^{2}b_{0}d_{4}d_{7}+168P^{2}b_{0}d_{3}d_{8}\\ +18P^{2}b_{2}d_{3}d_{6}+8P^{2}b_{2}d_{1}d_{8}+14P^{2}b_{2}d_{2}d_{7}+84P^{2}b_{1}d_{3}d_{7}+96P^{2}b_{1}d_{4}d_{6}+8P^{2}c_{8}d_{1}d_{2}+12P^{2}c_{7}d_{1}d_{3}\\ +24P^{2}c_{6}d_{2}d_{3}+16P^{2}c_{6}d_{1}d_{4}+32P^{2}c_{5}d_{2}d_{4}+20P^{2}c_{5}d_{1}d_{5}+48P^{2}c_{4}d_{3}d_{4}+40P^{2}c_{4}d_{2}d_{5}\\ +60P^{2}c_{3}d_{3}d_{5}+48P^{2}c_{3}d_{2}d_{6}+28P^{2}c_{3}d_{1}d_{7}+80P^{2}c_{2}d_{4}d_{5}+72P^{2}c_{2}d_{3}d_{6}+56P^{2}c_{2}d_{2}d_{7}\\ +96P^{2}c_{1}d_{4}d_{6}+84P^{2}c_{1}d_{3}d_{7}+64P^{2}c_{1}d_{2}d_{8}+36P^{2}c_{1}d_{1}d_{9}+120P^{2}c_{0}d_{5}d_{6}+112P^{2}c_{0}d_{4}d_{7}\\ +800\beta^{3}b_{0}^{2}b_{2}d_{5}^{2}+704\beta^{3}b_{0}^{2}b_{4}d_{4}^{2}+504\beta^{3}b_{0}^{2}b_{6}d_{3}^{2}+272\beta^{3}b_{0}^{2}b_{8}d_{2}^{2}+520\beta^{2}b_{0}^{3}d_{1}d_{10}+936\beta^{2}b_{0}^{3}d_{2}d_{9}\\ +1248\beta^{2}b_{0}^{3}d_{3}d_{8}+1456\beta^{2}b_{0}^{3}d_{4}d_{7}+1560\beta^{2}b_{0}^{3}d_{5}d_{6}+1800\beta^{2}b_{0}^{2}b_{1}d_{5}^{2}+1344\beta^{2}b_{0}^{2}b_{3}d_{4}^{2}\\ +432\beta^{2}b_{0}^{2}b_{7}d_{2}^{2}+120\beta^{2}b_{0}^{2}b_{9}d_{1}^{2}+320\beta b_{0}b_{1}^{2}d_{4}^{2}+180\beta b_{0}b_{2}^{2}d_{3}^{2}+80\beta b_{0}b_{3}^{2}d_{2}^{2}+20\beta b_{0}b_{4}^{2}d_{1}^{2}\\ +500\beta^{3}b_{0}b_{1}^{2}d_{5}^{2}+320\beta^{3}b_{0}b_{2}^{2}d_{4}^{2}+180\beta^{3}b_{0}b_{3}^{2}d_{3}^{2}+80\beta^{3}b_{0}b_{4}^{2}d_{2}^{2}+20\beta^{3}b_{0}b_{5}^{2}d_{1}^{2}+360\beta b_{0}^{3}d_{1}d_{9}\\ +640\beta b_{0}^{3}d_{2}d_{8}+840\beta b_{0}^{3}d_{3}d_{7}+960\beta b_{0}^{3}d_{4}d_{6}+640\beta b_{0}^{2}b_{2}d_{4}^{2}+360\beta b_{0}^{2}b_{4}d_{3}^{2}+160\beta b_{0}^{2}b_{6}d_{2}^{2}\\ +40\beta b_{0}^{2}b_{8}d_{1}^{2}-8\beta^{2}b_{0}^{2}d_{1}^{2}c_{9}-4\beta^{2}b_{0}^{2}d_{1}^{4}c_{9}+P^{2}c_{9}-10P^{2}b_{9}+320\beta^{2}b_{0}b_{3}^{2}d_{1}d_{4}+480\beta^{2}b_{0}b_{3}^{2}d_{2}d_{3}\\ +320\beta^{2}b_{0}b_{3}b_{4}d_{2}^{2}+80\beta^{2}b_{0}b_{3}b_{6}d_{1}^{2}+160\beta^{2}b_{0}b_{4}^{2}d_{1}d_{2}+80\beta^{2}b_{0}b_{4}b_{5}d_{1}^{2}+640\beta b_{0}^{2}b_{1}d_{1}d_{8}\\ +1440\beta b_{0}^{2}b_{1}d_{3}d_{6}+1600\beta b_{0}^{2}b_{1}d_{4}d_{5}+560\beta b_{0}^{2}b_{2}d_{1}d_{7}+960\beta b_{0}^{2}b_{2}d_{2}d_{6}+1200\beta b_{0}^{2}b_{2}d_{3}d_{5}\\ +480\beta b_{0}^{2}b_{3}d_{1}d_{6}+800\beta b_{0}^{2}b_{3}d_{2}d_{5}+960\beta b_{0}^{2}b_{3}d_{3}d_{4}+400\beta b_{0}^{2}b_{4}d_{1}d_{5}+640\beta b_{0}^{2}b_{4}d_{2}d_{4}\\ +480\beta b_{0}^{2}b_{5}d_{2}d_{3}+240\beta b_{0}^{2}b_{6}d_{1}d_{3}+160\beta b_{0}^{2}b_{7}d_{1}d_{2}+640\beta^{3}b_{0}b_{1}^{2}d_{2}d_{8}+840\beta^{3}b_{0}b_{1}^{2}d_{3}d_{7}\\ +960\beta^{3}b_{0}b_{1}^{2}d_{4}d_{6}+640\beta^{3}b_{0}b_{1}b_{3}d_{4}^{2}+360\beta^{3}b_{0}b_{1}b_{5}d_{3}^{2}+160\beta^{3}b_{0}b_{1}b_{7}d_{2}^{2}+40\beta^{3}b_{0}b_{1}b_{9}d_{1}^{2}\\ +280\beta^{3}b_{0}b_{2}^{2}d_{1}d_{7}+480\beta^{3}b_{0}b_{2}^{2}d_{2}d_{6}+600\beta^{3}b_{0}b_{2}^{2}d_{3}d_{5}+360\beta^{3}b_{0}b_{2}b_{4}d_{3}^{2}+160\beta^{3}b_{0}b_{2}b_{6}d_{2}^{2}\\ +40\beta^{3}b_{0}b_{2}b_{8}d_{1}^{2}+200\beta^{3}b_{0}b_{3}^{2}d_{1}d_{5}+320\beta^{3}b_{0}b_{3}^{2}d_{2}d_{4}+160\beta^{3}b_{0}b_{3}b_{5}d_{2}^{2}+40\beta^{3}b_{0}b_{3}b_{7}d_{1}^{2}\\ +120\beta^{3}b_{0}b_{4}^{2}d_{1}d_{3}+40\beta^{3}b_{0}b_{4}b_{6}d_{1}^{2}+640\beta^{2}b_{0}b_{1}^{2}d_{1}d_{8}+1120\beta^{2}b_{0}b_{1}^{2}d_{2}d_{7}+1440\beta^{2}b_{0}b_{1}^{2}d_{3}d_{6}\\ +1600\beta^{2}b_{0}b_{1}^{2}d_{4}d_{5}+1280\beta^{2}b_{0}b_{1}b_{2}d_{4}^{2}+720\beta^{2}b_{0}b_{1}b_{4}d_{3}^{2}+320\beta^{2}b_{0}b_{1}b_{6}d_{2}^{2}+80\beta^{2}b_{0}b_{1}b_{8}d_{1}^{2}\\ +480\beta^{2}b_{0}b_{2}^{2}d_{1}d_{6}+800\beta^{2}b_{0}b_{2}^{2}d_{2}d_{5}+960\beta^{2}b_{0}b_{2}^{2}d_{3}d_{4}+720\beta^{2}b_{0}b_{2}b_{3}d_{3}^{2}+320\beta^{2}b_{0}b_{2}b_{5}d_{2}^{2}\\ +80\beta^{2}b_{0}b_{2}b_{7}d_{1}^{2}+1296\beta^{2}b_{0}^{2}b_{1}d_{1}d_{9}+2304\beta^{2}b_{0}^{2}b_{1}d_{2}d_{8}+3024\beta^{2}b_{0}^{2}b_{1}d_{3}d_{7}+3456\beta^{2}b_{0}^{2}b_{1}d_{4}d_{6}\\ +1248\beta^{2}b_{0}^{2}b_{2}d_{1}d_{8}+2184\beta^{2}b_{0}^{2}b_{2}d_{2}d_{7}+2808\beta^{2}b_{0}^{2}b_{2}d_{3}d_{6}+3120\beta^{2}b_{0}^{2}b_{2}d_{4}d_{5}+1176\beta^{2}b_{0}^{2}b_{3}d_{1}d_{7}\\ +2016\beta^{2}b_{0}^{2}b_{3}d_{2}d_{6}+2520\beta^{2}b_{0}^{2}b_{3}d_{3}d_{5}+1080\beta^{2}b_{0}^{2}b_{4}d_{1}d_{6}+1800\beta^{2}b_{0}^{2}b_{4}d_{2}d_{5}+2160\beta^{2}b_{0}^{2}b_{4}d_{3}d_{4}\\ +960\beta^{2}b_{0}^{2}b_{5}d_{1}d_{5}+1536\beta^{2}b_{0}^{2}b_{5}d_{2}d_{4}+816\beta^{2}b_{0}^{2}b_{6}d_{1}d_{4}+1224\beta^{2}b_{0}^{2}b_{6}d_{2}d_{3}+648\beta^{2}b_{0}^{2}b_{7}d_{1}d_{3}\\ +456\beta^{2}b_{0}^{2}b_{8}d_{1}d_{2}+360\beta^{3}b_{0}b_{1}^{2}d_{1}d_{9}-128\beta^{2}b_{0}^{2}d_{1}^{3}c_{2}d_{8}-144\beta^{2}b_{0}^{2}d_{1}^{3}c_{1}d_{9}+280\beta b_{0}b_{1}^{2}d_{1}d_{7} \end{array}$$

$$\begin{array}{l} +320\beta b_{0}^{2}b_{5}d_{1}d_{4}+1120\beta b_{0}^{2}b_{1}d_{2}d_{7}+864\beta^{2}b_{0}^{2}b_{5}d_{3}^{2}+216P^{2}c_{4}d_{1}d_{2}d_{3}^{2}+144P^{2}c_{3}d_{1}^{2}d_{2}d_{6}\\ +32P^{2}c_{2}d_{1}d_{8}+24P^{2}c_{4}d_{1}d_{6}-30P^{2}b_{3}d_{3}d_{5}-64P^{2}b_{5}d_{2}d_{4}+96P^{2}c_{4}d_{2}^{3}d_{3}+24P^{2}c_{7}d_{1}^{2}d_{2}^{2}\\ +480\beta b_{0}b_{1}^{2}d_{2}d_{6}+600\beta b_{0}b_{1}^{2}d_{3}d_{5}+360\beta b_{0}b_{1}b_{3}d_{3}^{2}+160\beta b_{0}b_{1}b_{5}d_{2}^{2}+40\beta b_{0}b_{1}b_{7}d_{1}^{2}\\ +320\beta b_{0}b_{2}^{2}d_{2}d_{4}+160\beta b_{0}b_{2}b_{4}d_{2}^{2}+40\beta b_{0}b_{2}b_{6}d_{1}^{2}+120\beta b_{0}b_{3}^{2}d_{1}d_{3}+40\beta b_{0}b_{3}b_{5}d_{1}^{2}\\ -160\beta^{2}b_{0}^{2}d_{1}^{3}c_{0}d_{10}-32\beta^{2}b_{0}^{2}d_{1}^{3}c_{8}d_{2}-48\beta^{2}b_{0}^{2}d_{1}^{3}c_{7}d_{3}-64\beta^{2}b_{0}^{2}d_{1}^{3}c_{6}d_{4}-80\beta^{2}b_{0}^{2}d_{1}^{3}c_{5}d_{5}\\ -112\beta^{2}b_{0}^{2}d_{1}^{3}c_{3}d_{7}-96\beta^{2}b_{0}^{2}d_{1}^{2}c_{7}d_{2}^{2}-600\beta^{2}b_{0}^{2}d_{1}^{2}c_{1}d_{5}^{2}-384\beta^{2}b_{0}^{2}d_{1}^{2}c_{3}d_{4}^{2}+520\beta^{3}b_{0}^{2}b_{1}d_{1}d_{10}\\ +936\beta^{3}b_{0}^{2}b_{1}d_{2}d_{9}+1248\beta^{3}b_{0}^{2}b_{1}d_{3}d_{8}+1456\beta^{3}b_{0}^{2}b_{1}d_{4}d_{7}+1560\beta^{3}b_{0}^{2}b_{1}d_{5}d_{6}+576\beta^{3}b_{0}^{2}b_{2}d_{1}d_{9}\\ +1024\beta^{3}b_{0}^{2}b_{2}d_{2}d_{8}+1344\beta^{3}b_{0}^{2}b_{2}d_{3}d_{7}+1536\beta^{3}b_{0}^{2}b_{2}d_{4}d_{6}+608\beta^{3}b_{0}^{2}b_{3}d_{1}d_{8}+1064\beta^{3}b_{0}^{2}b_{3}d_{2}d_{7}\\ +1368\beta^{3}b_{0}^{2}b_{3}d_{3}d_{6}+1520\beta^{3}b_{0}^{2}b_{3}d_{4}d_{5}+616\beta^{3}b_{0}^{2}b_{4}d_{1}d_{7}+1056\beta^{3}b_{0}^{2}b_{4}d_{2}d_{6}+1320\beta^{3}b_{0}^{2}b_{4}d_{3}d_{5}\\ +600\beta^{3}b_{0}^{2}b_{5}d_{1}d_{6}+1000\beta^{3}b_{0}^{2}b_{5}d_{2}d_{5}+1200\beta^{3}b_{0}^{2}b_{5}d_{3}d_{4}+560\beta^{3}b_{0}^{2}b_{6}d_{1}d_{5}+896\beta^{3}b_{0}^{2}b_{6}d_{2}d_{4}\\ +496\beta^{3}b_{0}^{2}b_{7}d_{1}d_{4}+744\beta^{3}b_{0}^{2}b_{7}d_{2}d_{3}+408\beta^{3}b_{0}^{2}b_{8}d_{1}d_{3}+296\beta^{3}b_{0}^{2}b_{9}d_{1}d_{2}+648P^{2}c_{0}d_{1}d_{3}^{2}d_{6}\\ +600P^{2}c_{0}d_{1}d_{2}d_{5}^{2}+384P^{2}c_{0}d_{1}d_{2}^{2}d_{8}+360P^{2}c_{0}d_{1}^{2}d_{5}d_{6}+336P^{2}c_{0}d_{1}^{2}d_{4}d_{7}+288P^{2}c_{0}d_{1}^{2}d_{3}d_{8}\\ +216P^{2}c_{0}d_{1}^{2}d_{2}d_{9}-24P^{2}\beta b_{4}d_{2}d_{6}-14P^{2}\beta b_{4}d_{1}d_{7}+20P^{2}\beta b_{3}d_{4}d_{5}-34P^{2}\beta b_{9}d_{1}d_{2}\\ -66P^{2}\beta b_{7}d_{2}d_{3}-44P^{2}\beta b_{7}d_{1}d_{4}-64P^{2}\beta b_{6}d_{2}d_{4}-40P^{2}\beta b_{6}d_{1}d_{5}+72P^{2}c_{6}d_{1}^{2}d_{2}d_{3}\\ +240P^{2}c_{3}d_{1}d_{2}^{2}d_{5}+70P^{2}\beta b_{1}d_{1}d_{10}+180P^{2}c_{3}d_{1}^{2}d_{3}d_{5}+84P^{2}\beta b_{2}d_{3}d_{7}+36P^{2}\beta b_{2}d_{1}d_{9}\\ +576P^{2}c_{2}d_{2}^{2}d_{3}d_{4}+432P^{2}c_{2}d_{1}d_{3}^{2}d_{4}+384P^{2}c_{2}d_{1}d_{2}d_{4}^{2}-50P^{2}\beta b_{5}d_{2}d_{5}-30P^{2}\beta b_{5}d_{1}d_{6}\\ +192P^{2}c_{4}d_{1}d_{2}^{2}d_{4}+144P^{2}c_{4}d_{1}^{2}d_{3}d_{4}-30P^{2}\beta b_{4}d_{3}d_{5}+18P^{2}\beta b_{3}d_{3}d_{6}+14P^{2}\beta b_{3}d_{2}d_{7}\\ +126P^{2}\beta b_{1}d_{2}d_{9}+168P^{2}\beta b_{1}d_{3}d_{8}+196P^{2}\beta b_{1}d_{4}d_{7}+210P^{2}\beta b_{1}d_{5}d_{6}-60P^{2}\beta b_{5}d_{3}d_{4}\\ +96P^{2}c_{5}d_{1}^{2}d_{2}d_{4}-384\beta^{2}b_{0}^{2}c_{0}d_{3}d_{8}-288\beta^{2}b_{0}^{2}c_{0}d_{2}d_{9}-160\beta^{2}b_{0}^{2}c_{0}d_{1}d_{10}-128\beta^{2}b_{0}^{2}c_{6}d_{1}d_{2}^{3}\\ -384\beta^{2}b_{0}^{2}c_{4}d_{2}^{3}d_{3}-864\beta^{2}b_{0}^{2}c_{3}d_{2}^{2}d_{3}^{2}-512\beta^{2}b_{0}^{2}c_{3}d_{2}^{3}d_{4}-432\beta^{2}b_{0}^{2}c_{3}d_{1}d_{3}^{3}-864\beta^{2}b_{0}^{2}c_{2}d_{2}d_{3}^{3}\\ -640\beta^{2}b_{0}^{2}c_{2}d_{2}^{3}d_{5}-1536\beta^{2}b_{0}^{2}c_{1}d_{2}^{2}d_{4}^{2}-768\beta^{2}b_{0}^{2}c_{1}d_{2}^{3}d_{6}-1728\beta^{2}b_{0}^{2}c_{0}d_{3}^{3}d_{4}-896\beta^{2}b_{0}^{2}c_{0}d_{2}^{3}d_{7}\\ -1024\beta^{2}b_{0}^{2}c_{0}d_{1}d_{4}^{3}-32\beta^{2}b_{0}^{2}c_{8}d_{1}d_{2}-48\beta^{2}b_{0}^{2}c_{7}d_{1}d_{3}-96\beta^{2}b_{0}^{2}c_{6}d_{2}d_{3}-64\beta^{2}b_{0}^{2}c_{6}d_{1}d_{4}\\ -80\beta^{2}b_{0}^{2}c_{5}d_{1}d_{5}-192\beta^{2}b_{0}^{2}c_{4}d_{3}d_{4}-160\beta^{2}b_{0}^{2}c_{4}d_{2}d_{5}-96\beta^{2}b_{0}^{2}c_{4}d_{1}d_{6}-240\beta^{2}b_{0}^{2}c_{3}d_{3}d_{5}\\ -112\beta^{2}b_{0}^{2}c_{3}d_{1}d_{7}-320\beta^{2}b_{0}^{2}c_{2}d_{4}d_{5}-288\beta^{2}b_{0}^{2}c_{2}d_{3}d_{6}-224\beta^{2}b_{0}^{2}c_{2}d_{2}d_{7}-128\beta^{2}b_{0}^{2}c_{2}d_{1}d_{8}\\ -384\beta^{2}b_{0}^{2}c_{1}d_{4}d_{6}-336\beta^{2}b_{0}^{2}c_{1}d_{3}d_{7}-256\beta^{2}b_{0}^{2}c_{1}d_{2}d_{8}-144\beta^{2}b_{0}^{2}c_{1}d_{1}d_{9}-480\beta^{2}b_{0}^{2}c_{0}d_{5}d_{6}\\ -448\beta^{2}b_{0}^{2}c_{0}d_{4}d_{7}+8P^{2}\beta b_{3}d_{1}d_{8}+96P^{2}\beta b_{2}d_{4}d_{6}+288P^{2}c_{2}d_{1}d_{2}^{2}d_{6}+240P^{2}c_{2}d_{1}^{2}d_{4}d_{5}\\ +168P^{2}c_{2}d_{1}^{2}d_{2}d_{7}+864P^{2}c_{1}d_{2}d_{3}^{2}d_{4}+720P^{2}c_{1}d_{2}^{2}d_{3}d_{5}+576P^{2}c_{1}d_{1}d_{3}d_{4}^{2}+540P^{2}c_{1}d_{1}d_{3}^{2}d_{5}\\ +336P^{2}c_{1}d_{1}d_{2}^{2}d_{7}+288P^{2}c_{1}d_{1}^{2}d_{4}d_{6}+252P^{2}c_{1}d_{1}^{2}d_{3}d_{7}+192P^{2}c_{1}d_{1}^{2}d_{2}d_{8}+1152P^{2}c_{0}d_{2}d_{3}d_{4}^{2}\\ +1080P^{2}c_{0}d_{2}d_{3}^{2}d_{5}+960P^{2}c_{0}d_{2}^{2}d_{4}d_{5}+864P^{2}c_{0}d_{2}^{2}d_{3}d_{6}-5760\beta^{2}b_{0}^{2}c_{0}d_{1}d_{3}d_{4}d_{5}\\ -4032\beta^{2}b_{0}^{2}c_{0}d_{1}d_{2}d_{3}d_{7}-2304\beta^{2}b_{0}^{2}c_{3}d_{1}d_{2}d_{3}d_{4}-2880\beta^{2}b_{0}^{2}c_{2}d_{1}d_{2}d_{3}d_{5}-3840\beta^{2}b_{0}^{2}c_{1}d_{1}d_{2}d_{4}d_{5}\\ -3456\beta^{2}b_{0}^{2}c_{1}d_{1}d_{2}d_{3}d_{6}-3840\beta^{2}b_{0}^{2}c_{0}d_{2}^{2}d_{4}d_{5}-3456\beta^{2}b_{0}^{2}c_{0}d_{2}^{2}d_{3}d_{6}-2592\beta^{2}b_{0}^{2}c_{0}d_{1}d_{3}^{2}d_{6}\\ -2400\beta^{2}b_{0}^{2}c_{0}d_{1}d_{2}d_{5}^{2}-1536\beta^{2}b_{0}^{2}c_{0}d_{1}d_{2}^{2}d_{8}-576\beta^{2}b_{0}^{2}c_{5}d_{1}d_{2}^{2}d_{3}-960\beta^{2}b_{0}^{2}c_{3}d_{1}d_{2}^{2}d_{5}\\ -2304\beta^{2}b_{0}^{2}c_{2}d_{2}^{2}d_{3}d_{4}-1728\beta^{2}b_{0}^{2}c_{2}d_{1}d_{3}^{2}d_{4}-1536\beta^{2}b_{0}^{2}c_{2}d_{1}d_{2}d_{4}^{2}-864\beta^{2}b_{0}^{2}c_{4}d_{1}d_{2}d_{3}^{2}\\ -768\beta^{2}b_{0}^{2}c_{4}d_{1}d_{2}^{2}d_{4}+480\beta^{3}b_{0}b_{3}b_{4}d_{2}d_{3}+240\beta^{3}b_{0}b_{3}b_{5}d_{1}d_{3}+160\beta^{3}b_{0}b_{3}b_{6}d_{1}d_{2}\\ +1120\beta^{2}b_{0}b_{1}b_{2}d_{1}d_{7}+1920\beta^{2}b_{0}b_{1}b_{2}d_{2}d_{6}+2400\beta^{2}b_{0}b_{1}b_{2}d_{3}d_{5}+960\beta^{2}b_{0}b_{1}b_{3}d_{1}d_{6}\\ +1920\beta^{2}b_{0}b_{1}b_{3}d_{3}d_{4}+800\beta^{2}b_{0}b_{1}b_{4}d_{1}d_{5}+1280\beta^{2}b_{0}b_{1}b_{4}d_{2}d_{4}+640\beta^{2}b_{0}b_{1}b_{5}d_{1}d_{4}\\ +480\beta^{2}b_{0}b_{1}b_{6}d_{1}d_{3}+160\beta^{3}b_{0}b_{1}b_{8}d_{1}d_{2}+480\beta^{3}b_{0}b_{2}b_{3}d_{1}d_{6}+800\beta^{3}b_{0}b_{2}b_{3}d_{2}d_{5}+960\beta^{3}b_{0}b_{2}b_{3}d_{3}d_{4}\\ +400\beta^{3}b_{0}b_{2}b_{4}d_{1}d_{5}+640\beta^{3}b_{0}b_{2}b_{4}d_{2}d_{4}+320\beta^{3}b_{0}b_{2}b_{5}d_{1}d_{4}+480\beta^{3}b_{0}b_{2}b_{5}d_{2}d_{3}\\ +160\beta^{3}b_{0}b_{2}b_{7}d_{1}d_{2}+320\beta^{3}b_{0}b_{3}b_{4}d_{1}d_{4}+480\beta^{3}b_{0}b_{1}b_{6}d_{2}d_{3}+240\beta^{3}b_{0}b_{1}b_{7}d_{1}d_{3}+320\beta^{2}b_{0}b_{1}b_{7}d_{1}d_{2}\\ +800\beta^{2}b_{0}b_{2}b_{3}d_{1}d_{5}+1280\beta^{2}b_{0}b_{2}b_{3}d_{2}d_{4}+640\beta^{2}b_{0}b_{2}b_{4}d_{1}d_{4}+960\beta^{2}b_{0}b_{2}b_{4}d_{2}d_{3}\\ +320\beta^{2}b_{0}b_{2}b_{6}d_{1}d_{2}+480\beta^{2}b_{0}b_{3}b_{4}d_{1}d_{3}+320\beta^{2}b_{0}b_{3}b_{5}d_{1}d_{2}+864P^{2}c_{1}d_{1}d_{2}d_{3}d_{6}\\ -192\beta^{2}b_{0}^{2}c_{3}d_{2}d_{6}-128\beta^{2}b_{0}^{2}c_{5}d_{2}d_{4}+64P^{2}\beta b_{2}d_{2}d_{8}+120P^{2}c_{4}d_{1}^{2}d_{2}d_{5}-96\beta^{2}b_{0}^{2}d_{1}^{3}c_{4}d_{6} \end{array}$$

$$\begin{array}{l} +1600\beta^{2}b_{0}b_{1}b_{3}d_{2}d_{5}-4608\beta^{2}b_{0}^{2}c_{0}d_{1}d_{2}d_{4}d_{6}+216P^{2}c_{2}d_{1}^{2}d_{3}d_{6}-576\beta^{2}b_{0}^{2}d_{1}^{2}c_{3}d_{2}d_{6}\\ +960\beta^{2}b_{0}b_{1}b_{5}d_{2}d_{3}+144P^{2}c_{5}d_{1}d_{2}^{2}d_{3}-42P^{2}\beta b_{8}d_{1}d_{3}-216\beta^{2}b_{0}^{2}d_{1}^{2}c_{5}d_{3}^{2}+200\beta b_{0}b_{2}^{2}d_{1}d_{5}\\ +160\beta^{3}b_{0}b_{4}b_{5}d_{1}d_{2}+240\beta^{3}b_{0}b_{2}b_{6}d_{1}d_{3}+1440P^{2}c_{0}d_{1}d_{3}d_{4}d_{5}+480\beta^{2}b_{0}b_{2}b_{5}d_{1}d_{3}\\ +1152P^{2}c_{0}d_{1}d_{2}d_{4}d_{6}+1008P^{2}c_{0}d_{1}d_{2}d_{3}d_{7}+576P^{2}c_{3}d_{1}d_{2}d_{3}d_{4}+720P^{2}c_{2}d_{1}d_{2}d_{3}d_{5}\\ -1152\beta^{2}b_{0}^{2}c_{2}d_{1}d_{2}^{2}d_{6}-3456\beta^{2}b_{0}^{2}c_{1}d_{2}d_{3}^{2}d_{4}-864\beta^{2}b_{0}^{2}d_{1}^{2}c_{0}d_{2}d_{9}-288\beta^{2}b_{0}^{2}d_{1}^{2}c_{6}d_{2}d_{3}\\ -384\beta^{2}b_{0}^{2}d_{1}^{2}c_{5}d_{2}d_{4}-576\beta^{2}b_{0}^{2}d_{1}^{2}c_{4}d_{3}d_{4}-480\beta^{2}b_{0}^{2}d_{1}^{2}c_{4}d_{2}d_{5}-720\beta^{2}b_{0}^{2}d_{1}^{2}c_{3}d_{3}d_{5}\\ -960\beta^{2}b_{0}^{2}d_{1}^{2}c_{2}d_{4}d_{5}-864\beta^{2}b_{0}^{2}d_{1}^{2}c_{2}d_{3}d_{6}-672\beta^{2}b_{0}^{2}d_{1}^{2}c_{2}d_{2}d_{7}-1152\beta^{2}b_{0}^{2}d_{1}^{2}c_{1}d_{4}d_{6}\\ -1008\beta^{2}b_{0}^{2}d_{1}^{2}c_{1}d_{3}d_{7}-768\beta^{2}b_{0}^{2}d_{1}^{2}c_{1}d_{2}d_{8}-1440\beta^{2}b_{0}^{2}d_{1}^{2}c_{0}d_{5}d_{6}-1344\beta^{2}b_{0}^{2}d_{1}^{2}c_{0}d_{4}d_{7}\\ -1152\beta^{2}b_{0}^{2}d_{1}^{2}c_{0}d_{3}d_{8}+640\beta^{3}b_{0}b_{1}b_{2}d_{1}d_{8}+1120\beta^{3}b_{0}b_{1}b_{2}d_{2}d_{7}+1440\beta^{3}b_{0}b_{1}b_{2}d_{3}d_{6}\\ +560\beta^{3}b_{0}b_{1}b_{3}d_{1}d_{7}+960\beta^{3}b_{0}b_{1}b_{3}d_{2}d_{6}+1200\beta^{3}b_{0}b_{1}b_{3}d_{3}d_{5}+480\beta^{3}b_{0}b_{1}b_{4}d_{1}d_{6}\\ +960\beta^{3}b_{0}b_{1}b_{4}d_{3}d_{4}+400\beta^{3}b_{0}b_{1}b_{5}d_{1}d_{5}+640\beta^{3}b_{0}b_{1}b_{5}d_{2}d_{4}+320\beta^{3}b_{0}b_{1}b_{6}d_{1}d_{4}\\ +800\beta b_{0}b_{1}b_{2}d_{2}d_{5}+960\beta b_{0}b_{1}b_{2}d_{3}d_{4}+400\beta b_{0}b_{1}b_{3}d_{1}d_{5}+640\beta b_{0}b_{1}b_{3}d_{2}d_{4}+320\beta b_{0}b_{1}b_{4}d_{1}d_{4}\\ +480\beta b_{0}b_{1}b_{4}d_{2}d_{3}+240\beta b_{0}b_{1}b_{5}d_{1}d_{3}+160\beta b_{0}b_{1}b_{6}d_{1}d_{2}+320\beta b_{0}b_{2}b_{3}d_{1}d_{4}+480\beta b_{0}b_{2}b_{3}d_{2}d_{3}\\ +240\beta b_{0}b_{2}b_{4}d_{1}d_{3}+160\beta b_{0}b_{2}b_{5}d_{1}d_{2}+160\beta b_{0}b_{3}b_{4}d_{1}d_{2}+1600\beta^{3}b_{0}b_{1}b_{2}d_{4}d_{5}\\ +960P^{2}c_{1}d_{1}d_{2}d_{4}d_{5}+480\beta b_{0}b_{1}b_{2}d_{1}d_{6}+800\beta^{3}b_{0}b_{1}b_{4}d_{2}d_{5}) \end{array}$$
